# Heterogeneous photoredox flow chemistry for the scalable organosynthesis of fine chemicals

**DOI:** 10.1038/s41467-020-14983-w

**Published:** 2020-03-06

**Authors:** Can Yang, Run Li, Kai A. I. Zhang, Wei Lin, Katharina Landfester, Xinchen Wang

**Affiliations:** 1 State Key Laboratory of Photocatalysis on Energy and Environment, College of Chemistry, Fuzhou University, Fuzhou, 350002 P. R. China; 2 Max Planck Institute for Polymer Research, Ackermannweg 10, 55128 Mainz, Germany

**Keywords:** Heterogeneous catalysis, Organocatalysis, Photocatalysis, Chemical engineering

## Abstract

Large-scale photochemical synthesis of high value chemicals under mild conditions is an ideal method of green chemical production. However, a scalable photocatalytic process has been barely reported due to the costly preparation, low stability of photosensitizers and critical reaction conditions required for classical photocatalysts. Here, we report the merging of flow chemistry with heterogeneous photoredox catalysis for the facile production of high value compounds in a continuous flow reactor with visible light at room temperature in air. In the flow reactor system, polymeric carbon nitrides, which are cheap, sustainable and stable heterogeneous photocatalysts, are immobilized onto glass beads and fibers, demonstrating a highly flexible construction possibility for devices of the photocatalytic materials. As an example of the production of high value chemicals, important chemical structures such as cyclobutanes, which are basic building blocks for many pharmaceutical compounds, like magnosalin, are synthesized in flow with high catalytic efficiency and stability.

## Abstract (web summary)

Large-scale photochemical synthesis of high value chemicals is an ideal method of green chemical production. Here, the authors show the merging of heterogeneous carbon nitride photocatalysis with flow chemistry for the scalable organosynthesis of fine chemicals in a continuous flow reactor.

## Introduction

Photochemical synthesis of high valuable chemicals under mild reaction conditions is a green and sustainable manner for organic transformations, which has been actively pursued by chemists^1–3^. Many valuable natural compounds and pharmaceutical products have been synthesized in laboratory using photochemistry protocols^4,5^. However, when scaling up these laboratory-developed photocatalytic reactions in dimension-enlarged reactors for scalable productions, several associated issues came up^6^. One is the decrease in the irradiation efficiency. In principle, the light cannot penetrate deeply into the reaction mixtures with low surface-area-to-volume ratios in traditional batch reactors, leading to the decrease of productivity. In addition, a poor selectivity toward target products is often obtained when prolonging the reaction time to improve the conversion. The development and application of flow reactors is therefore recommended to conduct photochemical reactions, efficiently.

Continuous-flow chemistry with inherent advantages of efficient mass- and heat transfer and easy scale-up production has been extensively used for decades in the chemical industry^6–8^. The special module (e.g., narrow channel) of continuous-flow microreactor enables the uniform irradiation of the entire reaction mixtures, thus ensuring the maximum utilization of irradiation energy to drive photochemical reactions and avoiding the over-irradiation to induce the formation of byproducts. Some homogeneous continuous-flow photochemical systems^6^ have been developed for organic synthesis. Even though significant progress has been achieved in these homogenous flow systems, the issues inherited from homogenous photocatalysts still remain, in particular, the poor catalyst stability and the complicated process for catalyst separation and product purification. On this point, the use of heterogeneous photocatalyst in flow reactors ought to be a promising solution because of their facile separation and cost-effective feature. Nevertheless, the development of heterogeneous flow photochemistry has been greatly hampered by the solid and insoluble nature of heterogeneous photocatalysts, which could induce the blockage of flow systems and the shielding of light absorption. Therefore, a new design of a photoredox flow system is highly demanded, which involves suitable solid photocatalysts, reactors, and their immobilizations, as well as device fabrications in a harmony way.

Metal-free polymeric carbon nitride (PCN) photocatalyst has attracted extensive attentions owing to its outstanding properties such as tunable redox potentials, high photo- and thermal-stability, facile preparation, and easy processibility in various forms^9–11^. In fact, PCN has been frequently employed as a sustainable heterogeneous photocatalyst for various photoredox reactions including water splitting^12–15^, CO_2_ reduction^16,17^, and organic transformations. The ease engineering of PCN organic photocatalyst in band structure and surface functionality makes it particularly promising for various organic transformations^18,19^. For instance, the oxidations of primary benzylic amines^20^, alcohols^21^, and sulfides^22^ were reported under O_2_ atmosphere by PCN with a high conversion and selectivity. The sp^2^ C-H bond on phenyl ring could be activated by a PCN/Fe hybrid material^23^. C-C bond formation such as Suzuki coupling reaction has been achieved with palladium-loaded PCN^24^. Photocatalytic Diels–Alder reaction between electron-rich olefins and dienes was also achieved by PCN^25^, and very recently PCN-based organic photocatalysis has been further expanded to bifunctionalize arenes and heteroarenes^26^. Nonetheless, most of the above PCN photoredox catalysis systems mainly focus on milligram scale organic synthesis^20–26^, the scalable trial such as reaction in a large-scale batch using PCN as photocatalysts has been barely reported^27^. For continuous-flow reactor, carbon nitride solids were adhered on glass beads using abundant silica gel as the binder^28,29^ or transformed to water gel using large among of cross-linker^30^. Therefore, it is highly desired to develop an alternative strategy for carbon nitride-based continuous-flow reaction system in the field of photocatalytic organic synthesis.

Herein, we merge flow chemistry with heterogeneous PCN photocatalysis for the production of cyclobutanes through [2 + 2] cycloadditions with visible light. Cyclobutanes are found in a number of natural products^31,32^, and we chose magnosalin (US$ 150/mg) as our target molecule because it is a potent binder for glucocorticoid receptors in the treatment of inflammatory conditions. Some strategies such as Lewis-acid catalyzed reactions, amine-catalyzed reactions, and transition metal catalyzed reactions have been developed to realize the polarized [2 + 2] cycloadditions for the synthesis of cyclobutanes^33–36^. Yoon et al.^37,38^ reported a homogeneous dual-catalyst system including Ru(bpy)_3_Cl_2_ to achieve the asymmetric [2 + 2] photocycloaddition with visible light, and later some other homogeneous systems^39–43^ have also been proven to achieve this significant reaction with visible light. Also, some transition metal oxides^44,45^ (e.g., TiO_2_, CeO_2_) were designed as heterogeneous catalysts to achieve [2 + 2] photocycloaddition. Note that metal-free heterogeneous-catalyzed [2 + 2] photocycloaddition with high conversion and durability is still rarely achieved. In this paper, we develop a photoredox flow reactor using heterogeneous PCN for the scalable synthesis of high added-value fine chemicals, as exemplified here by cyclobutanes. Three different precursors are used to optimize the PCN photocatalyst, and the conceivable mechanism of symmetric or asymmetric photocycloaddition is investigated. PCN is immobilized on the surface of commercially available glass beads or glass fiber with an effective content of 1 wt.% and 1.2 wt.%, respectively (Supplementary Fig. 1). The functionalized glass beads or fibers can be easily constructed in a continuous-flow photoreactor with high light penetration, and cyclobutanes can be synthesized in gram scale in the continuous-flow photoreactor with a high yield of 81%. The success of this case is expected to open a new avenue for the application of heterogeneous photocatalysis in the green synthesis of added-value fine chemicals in flow.

## Results

### Structural characterization of PCN photocatalysts

**Fig. 1:**
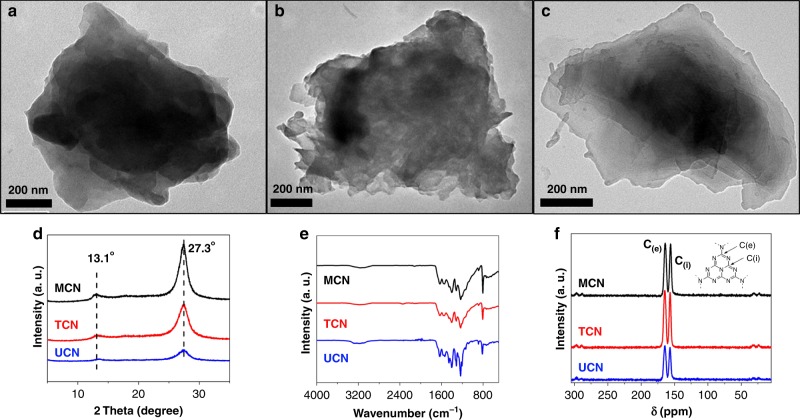
The morphological and structural characterization of PCNs. **a**–**c** TEM images of MCN, TCN, and UCN, respectively. **d** XRD pattern. **e** FT-IR spectrum. **f** Solid-state ^13^C MS-MAS NMR.

First, three different PCN materials were obtained by simply heating cheap and commercially available precursors such as urea, thiourea, and melamine, which were labeled as UCN, TCN, and MCN, respectively. Details on preparation procedures and characterizations are described in the Supplementary Information. The transmission electron spectroscopy (TEM) images showed different morphologies for the three PCN materials (Fig. 1a–c). UCN possessed a layered structure with a thickness of 10 nm–20 nm, whereas TCN and MCN showed a rather denser morphology with a thickness between ~300 nm and ~600 nm measured by atomic force microscope (AFM) displayed in Supplementary Fig. 2. The chemical structure of three PCNs was further confirmed by the powder X-ray diffraction (XRD) pattern, Fourier transform infrared (FT-IR) spectrum and solid-state ^13^C cross-polarization magic-angle-spinning NMR (^13^C CP-MAS NMR), respectively (as shown in Fig. 1d–f). These results are highly similar and consistent with the previous reports^9,10,13^. It was noted that the diffraction peak at ~13.1˚ corresponding to planar atomic structure kept same and the intensity of the (002) peak ~27.3˚ decreased from MCN to UCN, also illustrating the decrease of the thickness of three CNs. The physical and optical properties of the three PCNs including the Brunauer–Emmett–Teller (BET) surface areas, pore sizes, band gap, and conduction band (CB)/valance band (VB) position of these three CNs were listed in Table 1. The BET surface of MCN and TCN were both ~10 m^2^ g^−1^, whereas the BET surface of UCN was larger with ~52 m^2^ g^−1^. The average absorption pore width was obtained by the automatic system calculation from Barret–Joyner–Halenda (BJH) equation, which was showed in Supplementary Fig. 3. From the UV-vis diffuse reflection spectroscopy, optical band gaps in a range from 2.65 to 2.75 eV could be derived for three PCNs (Supplementary Fig. 4). The conduction band (CB) positions could be determined by the electrochemical Mott–Schottky plots (Supplementary Fig. 4c–e), and VB positions were calculated from above two values. All PCNs illuminated light activity properties as demonstrated in photocurrent measurement with clear response for the light-on and light-off events (Supplementary Fig. 5). The highest intensity indicated the most efficient light-induced electronic conductivity, as well as charge separation and charge transfer in UCN among PCNs^46^, which could be further illustrated by electrochemical impedance spectrum (EIS) in Supplementary Fig. 6.

**Table 1:** The physical and optical properties of three CNs.

Photocatalyst	BET surface (m^2^ g^−1^)	Pore diameter (nm)	Band gap (eV)	CB/VB position (V vs NHE)	Conversion in 8 h (%)
MCN	10	32.9	2.65	−1.29/1.36	~35%
TCN	10	33.1	2.67	−1.30/1.37	~83%
UCN	53	21.9	2.74	−1.36/1.38	>99%

### Photocatalytic performance on milligram scale

To test the photocatalytic efficiency of the PCN materials, visible light-induced [2 + 2] cycloaddition between trans-anethole and styrene was chosen as a model reaction. The monitoring experiments indicated that three PCN photocatalysts exhibited the same reaction character of second kinetic order for catalyzing [2 + 2] cycloaddition reaction under the irradiation of a white LED lamp (0.1 W/cm^2^) (Supplementary Fig. 7). However, reaction conversion differed after 8 h with 99%, 83%, and 35% for UCN, TCN, and MCN, respectively (Supplementary Fig. 8), which was in accordance with the results of photocurrent and EIS test. The highest photocatalytic efficiency for UCN probably resulted from reduced exciton-binding energy in its two dimensional structure, which likely promoted the light-induced charge transfer in CN sheets and thereby accelerated the electron transfer between the substrate and photocatalyst.

A series of control experiments with UCN photocatalyst were performed as listed in Supplementary Table 1 to optimize the reaction condition and reveal the possible interaction between the substrate and photocatalyst. Trace conversion was determined in darkness or without UCN, indicating the essential role and light-driven nature of the UCN photocatalyst (Supplementary Table 1, entry 4–5). Nitromethane was found to be the best reaction medium among tested solvents, while no product was obtained in DMSO and toluene (Supplementary Table 1, entry 6–10). In addition, molecular oxygen could accelerate the cycloaddition reaction, as an obvious decrease was found in nitrogen atmosphere (Supplementary Table 1, entry 11). Furthermore, specific electron and hole scavengers were added into the reaction to analyze the specific roles of photo-generated electron/hole pair. With the addition of KI, trace conversion was detected (Supplementary Table 1, entry 12). Although K_2_S_2_O_8_ was added, both conversion and selectivity decreased markedly (Supplementary Table 1, entry 13).

Based on the observations and previous reports^47–51^, we proposed the reaction mechanism with the oxidation of trans-anethole by the photo-generated hole as the initiation step (Supplementary Fig. 9). As the VB of UCN lay at 1.36 V vs. SCE, it was sufficient to oxidize trans-anethole (*E*_oxi._ = 1.17 V vs. SCE)^52^. Then, the generated cationic radical underwent the [2 + 2] cycloaddition with another alkene partner to form a cyclic intermediate, which afterwards got an electron from the photocatalyst UCN, the superoxide radical or the reactive neutral trans-anethole to form the final cyclobutane product. As shown in Supplementary Fig. 10, oxygen could quench the fluorescence of UCN suspension with high efficiency for several cycles, indicating that electron transferred from photocatalyst to oxygen. The electron spin resonance (EPR)-trapping experiment using 5,5-dimethyl-1-pyrroline N-oxide (DMPO) as active oxygen species trapping agent revealed typical EPR patterns for superoxide radical (O_2_^•−^) adducts (DMPO-O_2_^•−^) (Supplementary Fig. 11a). In addition, with N-tert-butyl-α-phenylnitrone (PBN) as radical trapping agent in EPR trapping experiment showed the characteristic patterns of the obtained PBN-radical, which indicated clearly the formation of the cationic radical of anethole (Supplementary Fig. 11b). The region- and steoro-selectivity was attributed to the sterically favored bond formation at β-position of the double bond than that at α position^49^.

**Fig. 2:**
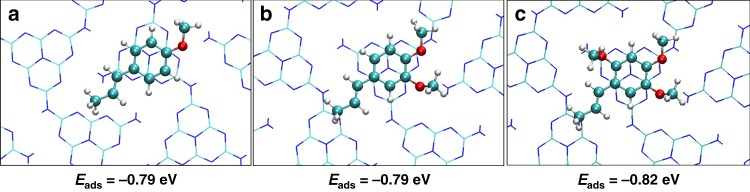
The DFT calculation of absorption model. Optimized structures and adsorption energies of **a** anethole. **b** Methyl isoeugenol. **c** α-asarone on the surface of UCN.

To further understand the adsorption patterns between anethole derivatives and UCN, the possible interaction models were calculated. As shown in Fig. 2, one π–π interaction is formed in all three systems. The π-π interaction between the electron-rich PCN and aromatic molecules is beneficial for activation of the molecules by promoting further charge transfer. The adsorption energies of anethole derivatives on UCN are ~−0.8 eV, which indicates that the main contribution of the adsorption is from the π-π interactions, while the –OCH_3_ group has an ignorable effect.

### Photocatalytic performance on gram scale

As an important example of potential industrial application, magnosalin was prepared in a gram-scale batch condition with UCN photocatalyst. Starting with 4 g *α*-asarone (the moles of substrate increased by factor of 80, 0.1 m), CH_3_NO_2_ (200 mL), and 200 mg UCN (Supplementary Fig. 12), the conversion of *α*-asarone reached 79%, but the selectivity to the product, magnosalin, was 63% (with an isolated yield of 48%) (Table 2, entry 1). The decreased yield was mainly attributed to more oxide side-products formed because of the reaction heat from the amplification system. In order to demonstrate the scalability of our reaction condition and the possibility of carbon nitride as a highly efficient photocatalyst for large-scale preparation of pharmaceutical product, a new type of heterogeneous photoredox flow system was designed and constructed.

**Table 2:** Screening and control experiment of gram-scale [2 + 2] dimerization of *α-*asarone catalyzed by UCN under white light^a^.


Entry	Light	Reactor	Reaction condition variations	Yield^b^
1	+	Batch	UCN powders^c^	48%
2	+	Flow	UCN@glass fibers^d^	70%
3	+	Flow	UCN@glass beads	81%
4	−	Flow	In dark	Trace
5	+	Flow	In acetonitrile	2%^e^
6	+	Batch	UCN@glass beads	53%
7^f^	+	Flow	UCN@glass beads	87%

^a^Standard reaction conditions of flow system: (*α-*asarone) = 0.167 m, *V*_CH3NO2_ = 60 mL, white LED lamp (0.1 W/cm^2^), room temperature, air, irradiation time 48 h.

^b^Isolated yield.

^c^The amount of UCN is 200 mg.

^d^The amount of UCN coated on glass fibers is 80 mg.

^e^Conversion from GC-MS.

^f^Using a photoreactor assembled from six paralleled glass tubes with irradiation time 8  h.

**Fig. 3:**
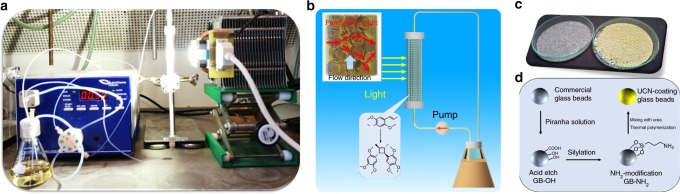
The construction of the fixed-bed photoreactor. **a** Photograph of the flow-continuous photoreactor. **b** Scheme of fixed-bed photoreactor filled with supported carbon nitrides for magnosalin production. **c** Commercial glass beads (left) and UCN-coated glass beads (right). **d** General process for UCN coating on the surface of glass beads.

With the optimized reaction condition on hand, a photochemical continuous-flow setup was performed in order to simulate the general process of industrial production (Fig. 3a, b). Here, the photocatalyst UCN was immobilized into a photoreactor (*d* = 0.7 cm, l = 7 cm) to construct a heterogeneous flow reaction system. Originally, commercially available glass fiber was chosen as the support to immobilize the photocatalyst UCN. Precisely, a UCN-coated glass fiber could be obtained via direct thermal condensation of urea on the surface of fresh fiber (detailed information in Methods). As shown in Supplementary Fig. 13a, b, the fresh glass fiber demonstrated a smooth and glassy surface, the surface became rough and flake-decorated after coating with UCN photocatalyst. Strong blue emission could be clearly observed in fluorescence microscope images (Supplementary Fig. 13c, d), indicating that glass fiber was successfully covered with UCN photocatalyst. The average thickness of the UCN layer was ~20 nm (Supplementary Fig. 13b) and the amount was estimated to be 1.2 wt.% after calcination. Subsequently, the UCN-coating glass fiber was filled into photoreactor. Herein, the cycloaddition of *α*- asarone was employed in flow because the generated product magnosalin is an important anti-inflammatory agent^53^. As expected, a high reaction conversion of 79% for *α*-asarone and yield of 70% for magnosalin was achieved with almost quantitative selectivity (Table 2, Entry 2, and Supplementary Fig. 14a), which indicated a high catalytic efficiency of UCN photocatalyst and feasibility of creating a heterogeneous flow photochemistry. Unfortunately, a decreased conversion was found during the repeating experiments (Supplementary Fig. 14a). The possible reason lay in the separation and loss of UCN from glass fiber due to the high pressure and speed during the flow process, which was also demonstrated by the decrease of absorption for the UCN-coating glass fibers after 50 h and 200 h, respectively (Supplementary Fig. 13e). In order to enhance the binding between support and photocatalyst UCN, a revised coating method needed to be designed.

**Fig. 4:**
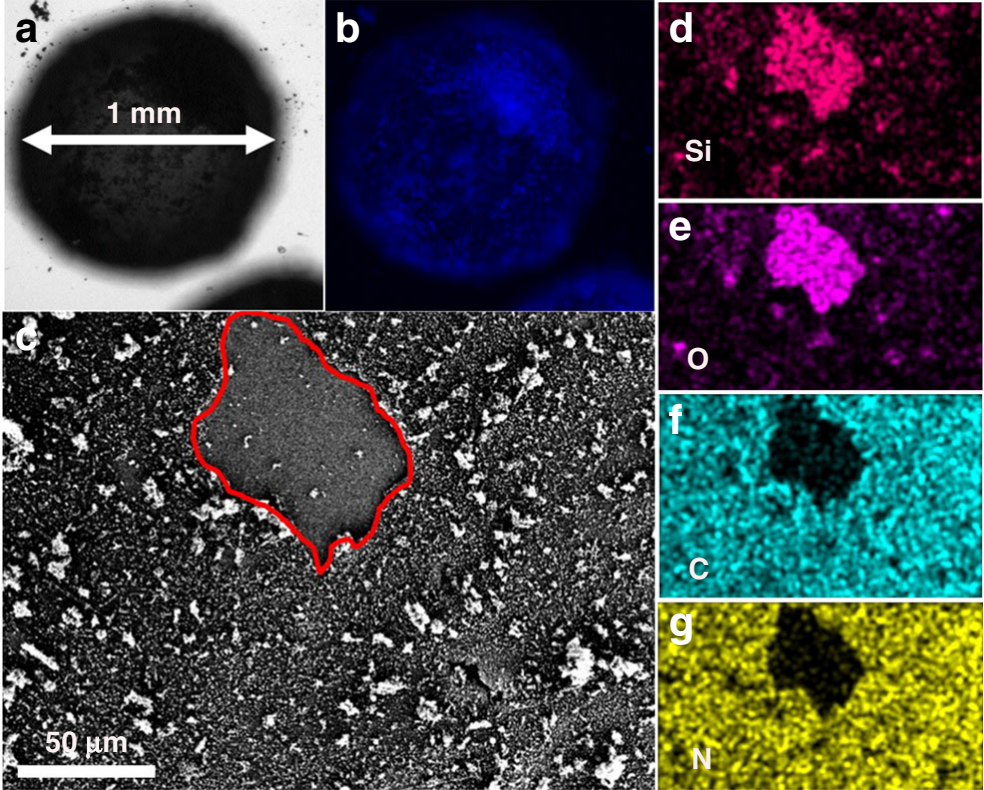
Fluorescence and SEM images of UCN@glass beads. **a** Optical microscope image and **b** fluorescence image with scale of 1 mm. **c** SEM image (the red marking region is a man-made removal of carbon nitride to distinguish the surface of UCN and glass beads). **d**–**g** Element mapping images. Elements from top to bottom are Si, O, C, and N, respectively.

Afterwards, commercially available glass beads with diameter of 1 mm were employed as another support for UCN immobilization through a modified approach. With details described in methods (see in Fig. 3d), the glass beads were successively etched with piranha solution, decorated with silylation reagent (3-aminopropyltriethoxysilane, APTES) and coated with UCN via thermal condensation of urea, thus producing the final UCN-coated glass beads (showed in Fig. 3c). The introduction of APTES could function as bridge for connecting glass bead and UCN (Supplementary Fig. 15). With this modified method, the obtained UCN@glass beads showed obvious yellow and rough surface (Fig. 3c). In addition, the glass beads displayed a strong blue fluorescence, indicating that the surface was completely covered with UCN photocatalyst (Fig. 4a, b). The thickness of the UCN flake was 5~10 nm from SEM (Fig. 4c) and the amount was calculated to be ~1 wt. % from calcination test. The element mapping on the deliberately damaged surface demonstrated a clear separation between support and photocatalyst, offering a direct proof for the successfully coating of UCN on the surface of glass beads (Fig. 4d–g). Chemical structure and morphology of UCN coated on the surface of glass beads were further demonstrated by XRD (Supplementary Fig. 16a), FT-IR (Supplementary Fig. 16b), XPS (Supplementary Fig. 17), and SEM (Supplementary Fig. 18). In addition, the MCN and TCN on the surface of glass beads were characteristic by PL (Supplementary Fig. 19), FT-IR (Supplementary Fig. 20) and XPS (Supplementary Fig. 21) to show the universality of this method. At last, UCN-coated glass beads exhibited a highly flexible processing possibility in various shapes, as demonstrated in Supplementary Fig. 22.

The obtained UCN-coated glass beads were then employed to test the photocatalytic efficiency and stability in continuous-flow condition. In all, 8 g UCN-coated glass beads were filled in the flow photoreactor with *α*-asarone concentration being 0.167 m in 60 mL CH_3_NO_2_ and a flow speed of 0.5 mL min^−1^. As shown in Table 2 (Entry 3), a comparable catalytic efficiency was achieved. The reaction conversion reached 61% after 24 h and further increased to 89% after 48 h with a final isolated yield of 81%, corresponding to 1.6 g magnosalin. No conversion was determined in darkness and trace conversion was detected in other reaction medium such as acetonitrile (Table 2, Entry 4–5). Moreover, an obviously improved stability was found with UCN-coated glass beads. In repeating experiments, the reaction conversion stabilized in five cycles without obvious decrease (Supplementary Fig. 14b). Therefore, the turn over number for the reaction was calculated with a value of 106 based on the moles of UCN on the surface of glass beads for total five cycles. This improvement is mainly attributed to the strong binding force between the glass beads and UCN photocatalyst in the modified approach, which offers a miniature for continuous industrial pharmaceutical compound, magnosalin, production. The flow reactor delivered a much higher reaction yield than that in batch (Table 2, Entry 6), indicative of a great advantage in the flow reaction system. AQY measurment of both batch and flow system under a 420 nm monochromatic light (Supplementary Fig. 23) was compared with further demonstrate the advantage of the continuous-flow system for scale-up (description in detail shown in Supplementary Discussion and Supplementary Figs. 24 and 25). The photocatalytic efficiency can be further improved when using a photoreactor assembled from six paralleled glass tubes (Supplementary Fig. 26) to enlarge the irradiation surface area (Table 2, Entry 7 vs. Entry 2).

**Fig. 5:**
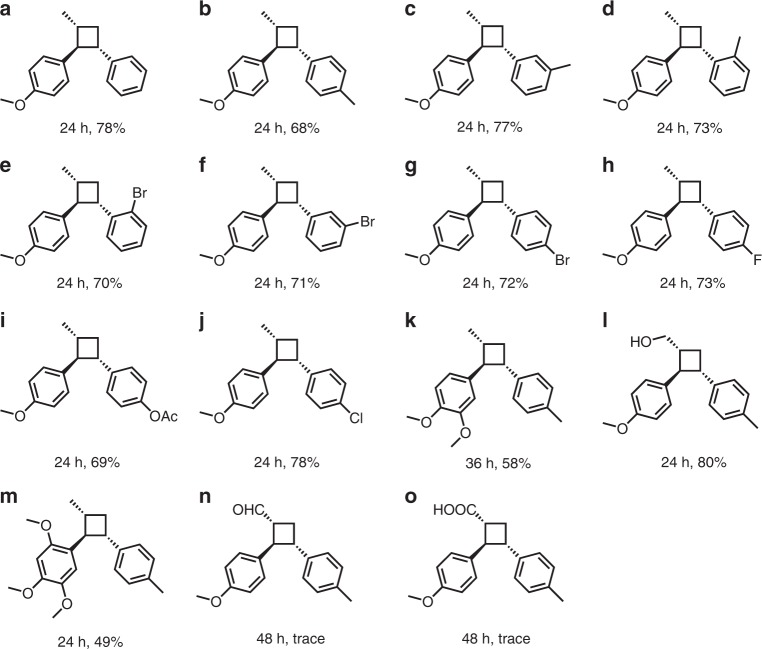
Application of the flow reactor in heterogeneous photosynthesis. Scope of unsymmetrical [2 + 2] cycloaddition reaction.

At last, to demonstrate the general accessibility of the [2 + 2] cycloaddition reaction with UCN photocatalyst, the unsymmetrical [2 + 2] cycloaddition reaction was tested with UCN-coated glass beads in continuous-flow photoreactor under the visible light irradiation. As showed in Fig. 5, various styrene derivatives with both electron-donating groups such as methyl (b–d) and electron-withdrawing groups such as ester (i) and halides (e–j) were accessible for the reaction with remarkable reaction yield. Although some side-reactions such as dimerizaztion and oxidation of anethole may present, the crossed-cycloaddition with styrene derivatives always took place as the major pathway, with a considerable amount of unsymmetrical cyclobutane products being achieved. In addition, the position of the functional group had slight influence on the reaction yield. Both methyl-substituted (b–d) and bromine-substituted (e–g) styrene on *ortho-*, *meta-*, or *para-* positions demonstrated generally comparable reaction yield, respectively. Furthermore, anethole derivatives, such as methyl isoeugenol (k), methoxycinnamyl alcohol (l), and asarone (m) were also tolerated with moderate reaction yield. The slight lower conversion for methyl isoeugenol and asarone starting compound may be resulted from the over-oxidation and decomposition of electron-rich cycloadduct intermediates. However, trace products were determined even after a longer reaction time (48 h) when *p*-methoxycinnamaldehyde (n) and *p*-methoxycinnamic acid (o) were employed as reactive substrates, probably due the high oxidation potentials of the starting compounds. Although many reports have been conducted about cyclobutanes preparation, direct synthesis of pharmaceutical product, such as magnosalin, as far as we know, is still rare. The UCN-catalyzed [2 + 2] cycloaddition in continuous-flow reaction system offers a green approach for drugs production in a mild condition.

## Discussion

The most-challenging feature of using photocatalysts in heterogeneous reaction lies in the terms of the light absorption, design of photocatalytic devices and a balance between activity and stability during the amplification reaction. We have demonstrated the use of carbon nitride as cheap, processable, and stable photocatalyst in scalable and continuous-flow system for cyclobutanes production, especially for magnosalin preparation in gram scale. Both commercial available glass fiber and glass beads have been employed as translucent support for the carbon nitrile photocatalyst. The flow photoreactor with UCN-coated glass beads demonstrates the highest reaction yields. Unsymmetrical [2 + 2] cycloaddition with extended styrene derivatives have been conducted with UCN photocatalyst with considerable conversion. The flow photochemical system is compatible for crossed [2 + 2] cycloaddition reaction with corresponding cyclobutanes being achieved with a remarkable yield. We believe that this study can demonstrate an important step toward the utilization of carbon nitride for photo-catalyzed industrial production of fine chemicals under mild and sustainable reaction conditions.

## Methods

### Synthesis of polymeric carbon nitride

Polymeric carbon nitride was synthesized using three different precursors, i.e., urea, thiourea, melamine. Typically, a lidded high-quality alumina crucible was charged with the precursor (15 g) and placed inside a muffle furnace. Then the temperature was heated up to 550 °C with a rate of 0.5 °C/minute and stabilized for 3 h. After cooling down to the ambient temperature, the resultant powder was washed with water, HCl, NaOH solution, respectively, to remove all the unreacted and potentially detrimental surface species. The products based on the precursors were denoted as UCN, TCN, and MCN, respectively.

### Synthesis of UCN-coating glass fibers

The glass fibers were first cleaned in piranha solution (H_2_O_2_: H_2_SO_4_ = 1:3) to remove the surficial contaminants. Then, the glass fibers were immersed into a saturated urea solution (10 g/mL), followed by reflux at 60 °C for 12 h. After water evaporation at 80 °C for overnight, the crude mixture was heated in a muffle furnace with the same condition as that of UCN synthesis. A UCN-coated glass fiber could be obtained after directly thermal condensation polymerization on the surface of glass fiber.

### Synthesis of UCN-coating glass beads

As shown in Fig. 3d, commercially available glass beads were first cleaned and etched with piranha solution. Then, silylation reaction was conducted in order to introduce an amine function group. Precisely, a 20 mL anhydrous toluene was added with acid etched glass beads and APTES and the mixture was reflux for 24 h. Afterwards, the glass beads were collected from the mixture and washed with dichloromethane, and dried in vacuum oven at 60 °C for overnight. The amine functionalized glass beads were mixed with urea with a mass ratio of 1:1, and then heated in a muffle furnace using the same heating program as that of UCN synthesis to produce UCN-coated glass beads.

### Photosynthesis of cyclobutanes in batch condition

A flame-dried 40 ml vial was charged with UCN (2.4 mg), *E*-anethole (50.0 mg, 0.24 mmol), and CH_3_NO_2_ (2.4 ml). Then, 10 equivalent of styrene derivatives partner was added into the reaction mixture. Afterwards, the vial was placed under the irradiation of a white LED lamp (0.1 W/cm^2^) in air condition. The conversion of was determined by GC-MS measurement based on the consumption of anethole derivatives. After the reaction was finished, the mixture was poured into a separatory funnel and extracted with a mixture of 60 ml Et_2_O and water (v/v, 1/1). A crude product was received after collecting the organic layer, drying over anhydrous MgSO_4_, and concentrating with rotary evaporator. Finally, the pure cycloaddition products could be obtained after further purification with flash-column chromatography.

### Photosynthesis of cyclobutanes in continuous-flow reactor

As showed in Fig. 3a, a 100 ml conical flask was charged with α-asarone (10 mmol) and 60 ml CH_3_NO_2_. A transparent glass tube (*d* = 0.7 cm, *l* = 7 cm) with sealed bottoms was filled with UCN-coated glass beads and used as photoreactor in flow system. Then, creep pump was employed to connect the photoreactor and reaction solution. The photoreactor was placed in front of a white LED light with a distance of 5 cm. The rate of flow was 0.5 ml min^−1^ and the conversion was recorded by GC-MS measurement. The flow reaction with glass fiber was conducted with a similar procedure while began with a same starting concentration of α-asarone (0.167 mol/L). For the photocatalytic unsymmetrical [2 + 2] cycloaddition reaction, the amount of reaction substrates was started as follows: *E*-anethole and its derivatives (1 equivalent), styrene (10 equivalent), and CH_3_NO_2_ (60 mL).

## Supplementary information

Supplementary Information

Description of Additional Supplementary Files

Supplementary Data 1

## Data Availability

All data supporting the findings of this study are available within the article as well as the Supplementary Information file, or available from the corresponding authors on reasonable request.
