# Supplementary material for: Heterogeneous photoredox flow chemistry for the scalable organosynthesis of fine chemicals
Source: Nat Commun. 2020 Mar 6;11:1239. doi: 10.1038/s41467-020-14983-w (PMC7060272; doi:10.1038/s41467-020-14983-w)
Supplement: Supplementary file 1 — Supplementary Information [file 41467_2020_14983_MOESM1_ESM.pdf]

Supplementary Information

Heterogeneous Photoredox Flow Chemistry for the Scalable Organosynthesis of Fine Chemicals

Yang et al.

Supplementary Figures

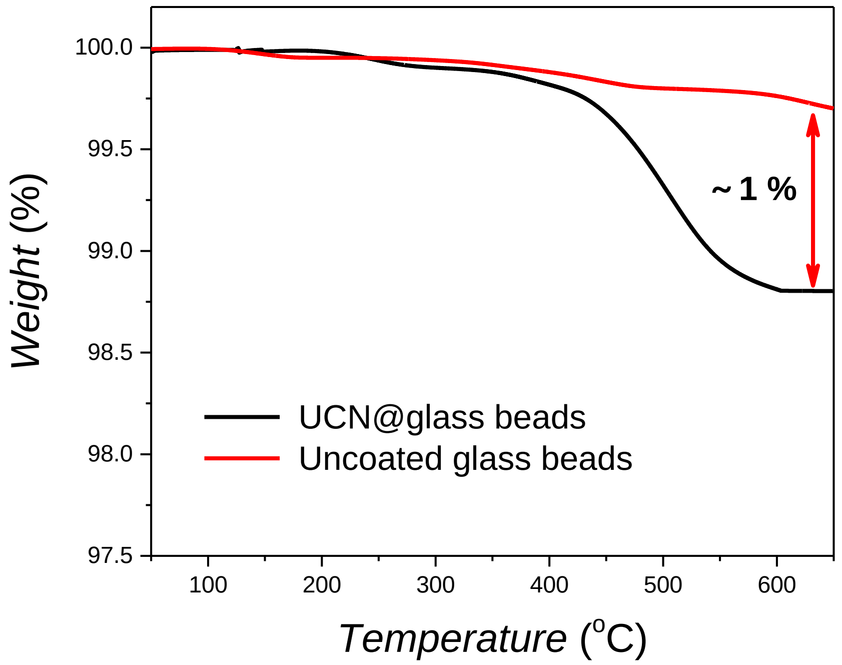

Supplementary Figure 1. TGA curves of UCN@glass beads and uncoated glass beads.

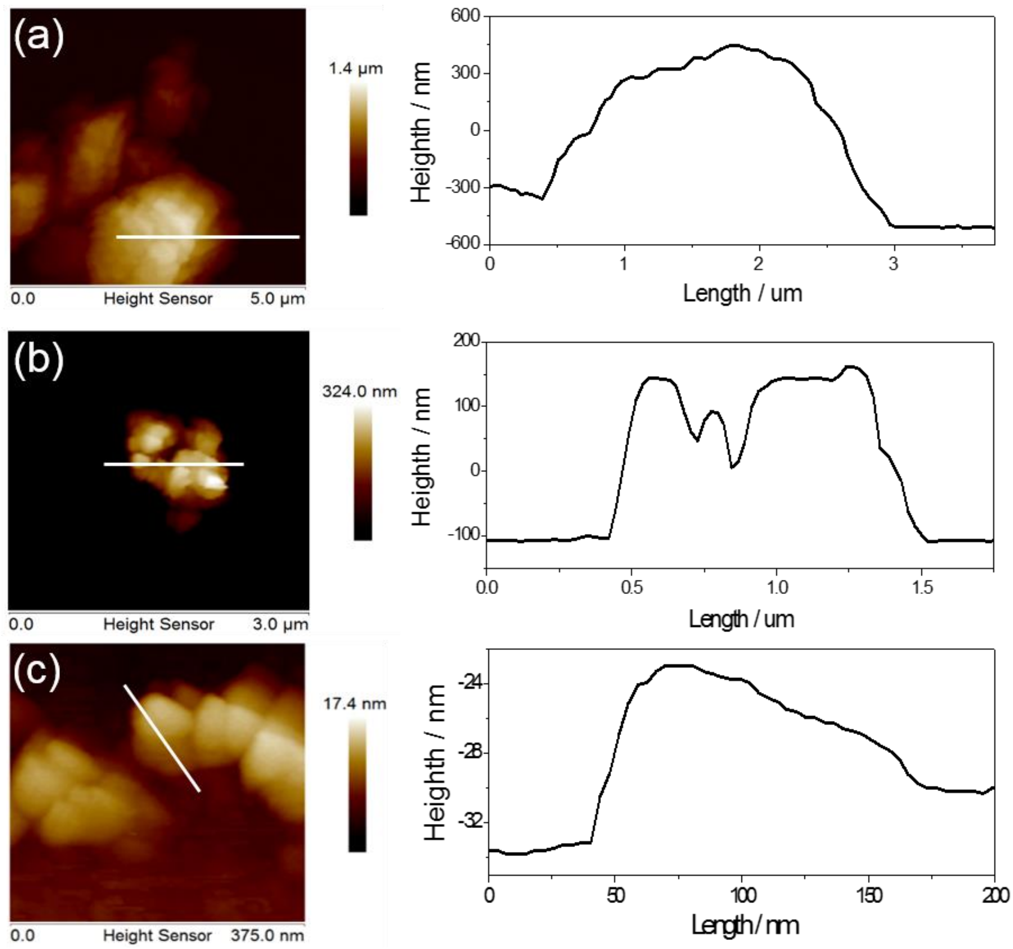

Supplementary Figure 2. Characterization of Atomic Force Microscope. a MCN; **b** TCN and **c** UCN.

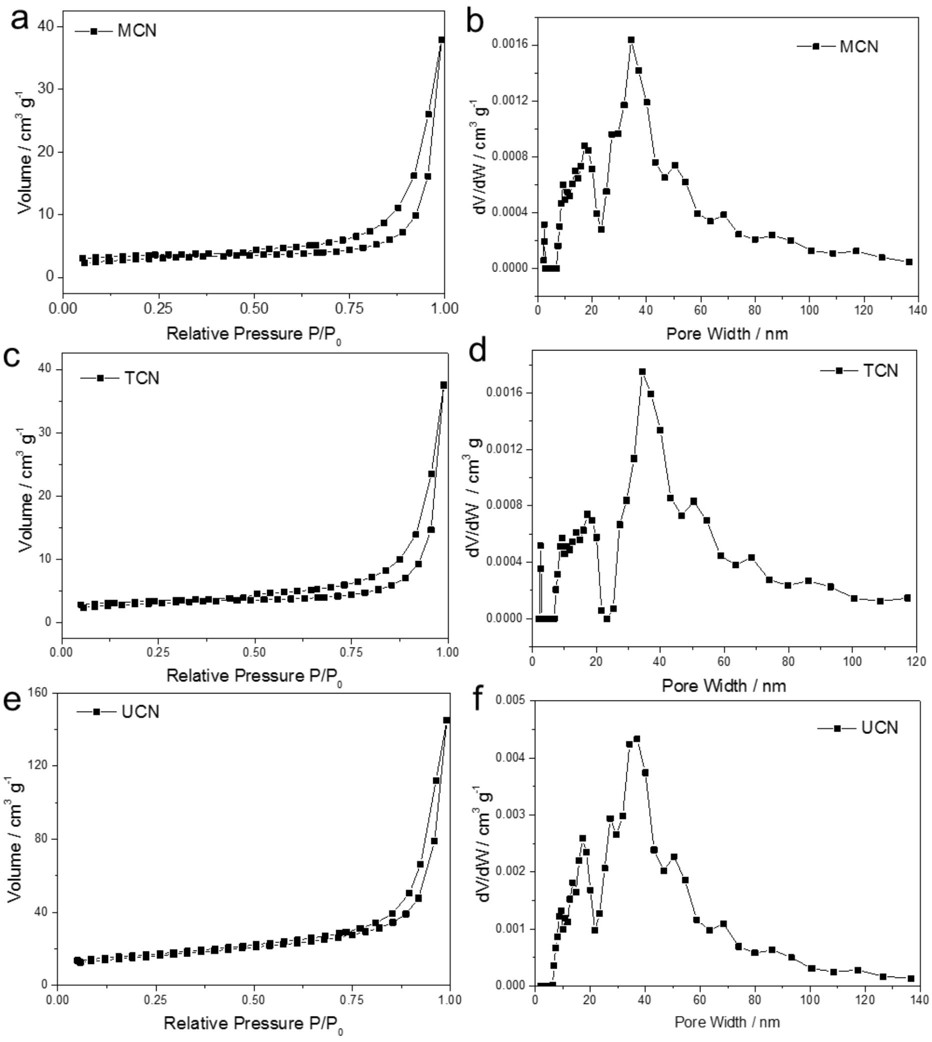

Supplementary Figure 3. **N_2 gas absorption-desorption isotherm and pore size distribution measured at 77 K. a-b** MCN; **c-d** TCN and **e-f** UCN.

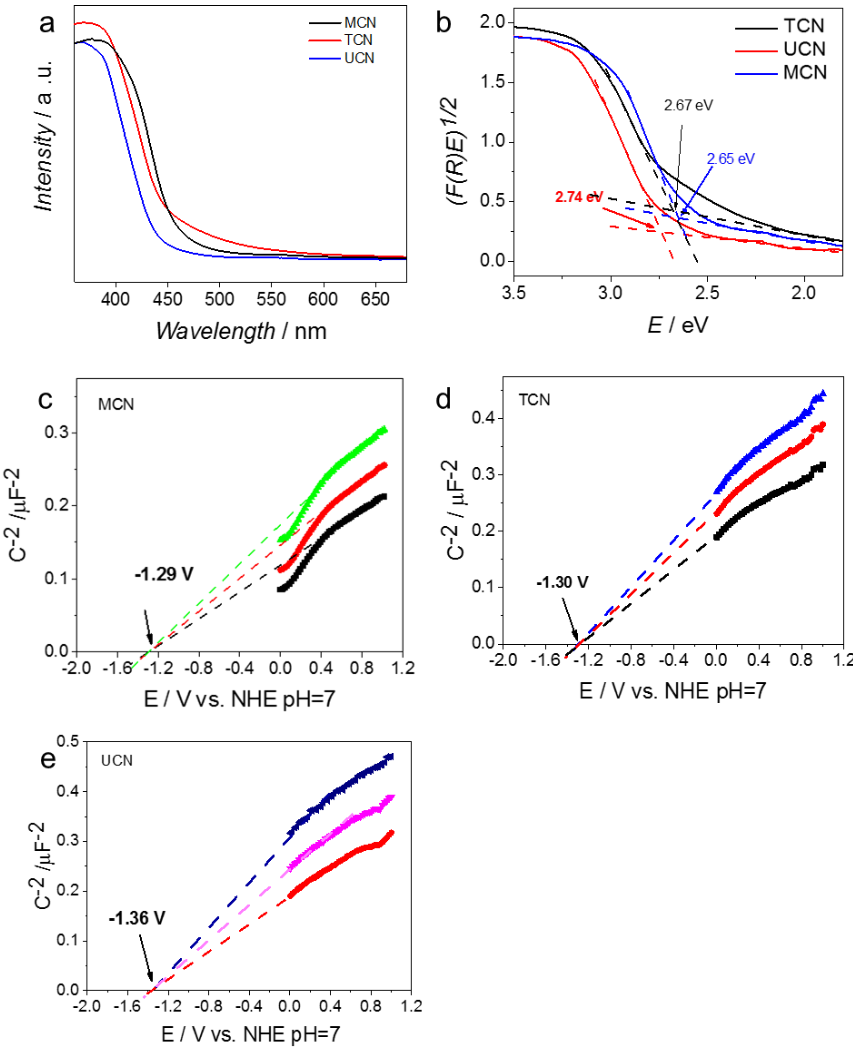

Supplementary Figure 4. Band gaps of PCNs. a UV-vis Diffuse Reflect Spectrum of three CNs; **b** Corresponding Tauc plot; **c-e** Mott-Schottky plots of MCN, TCN and UCN in 0.2 M Na_2SO_4 solution, pH=7.

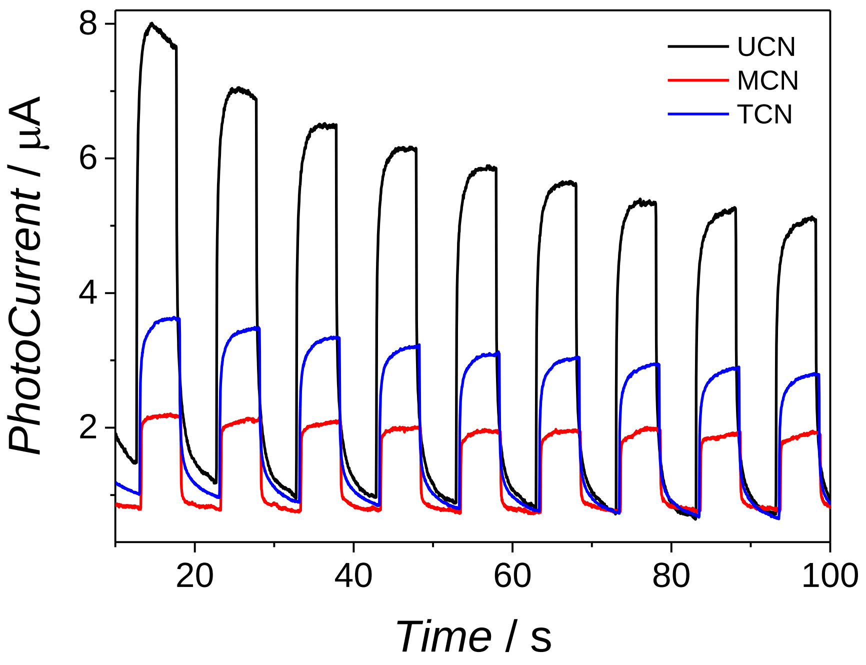

Supplementary Figure 5. Photocurrent of CNs measured with 0.3 V bias potential in 0.2 M Na_2SO_4 solution, pH=7

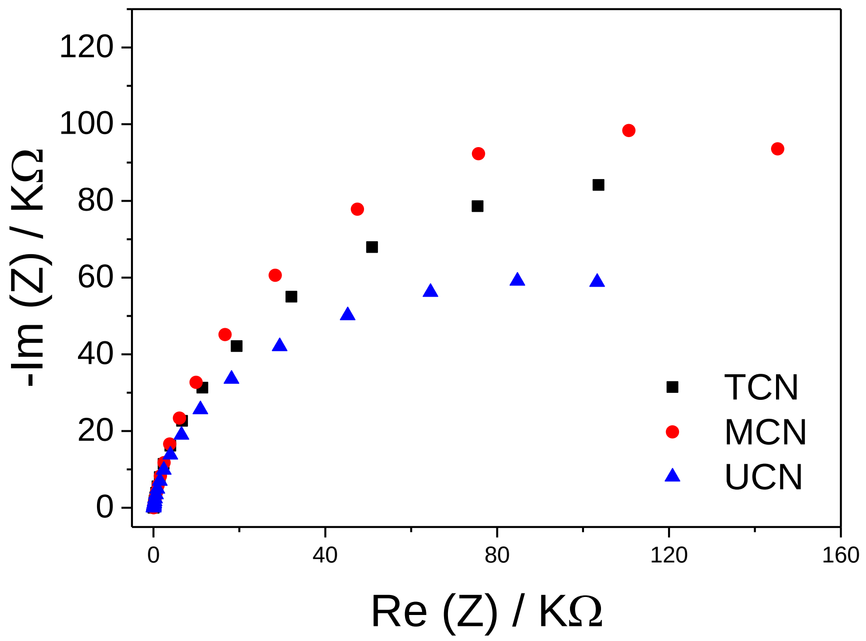

Supplementary Figure 6. Nyquist plots of the samples.

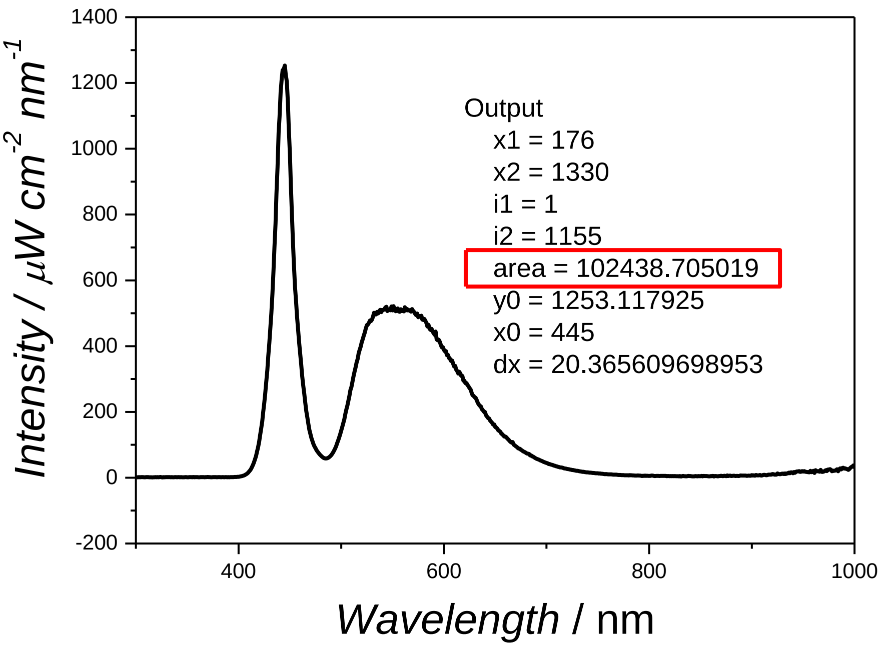

Supplementary Figure 7. The irradiance spectrum of the white LED lamp.

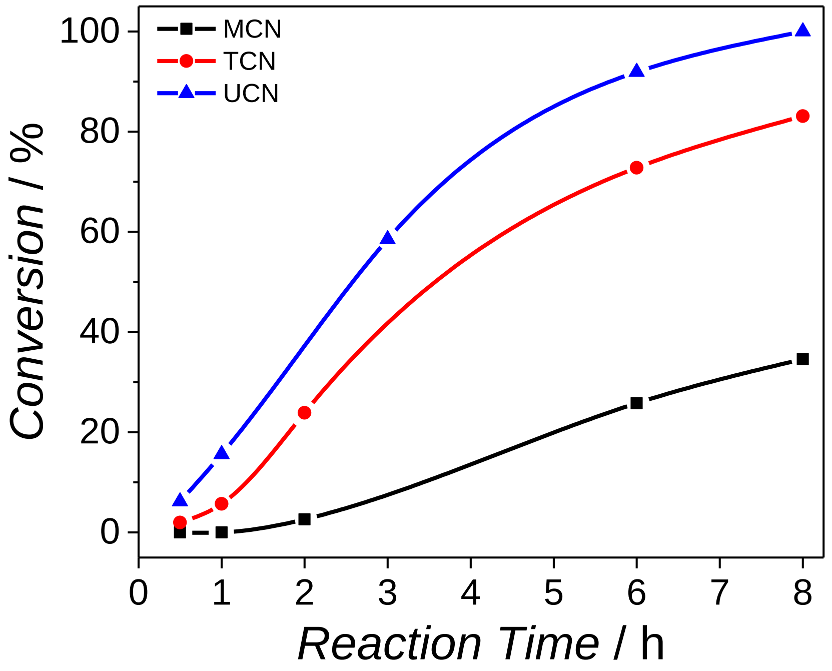

Supplementary Figure 8. Time dependence of substrate conversion using MCN, TCN and UCN.

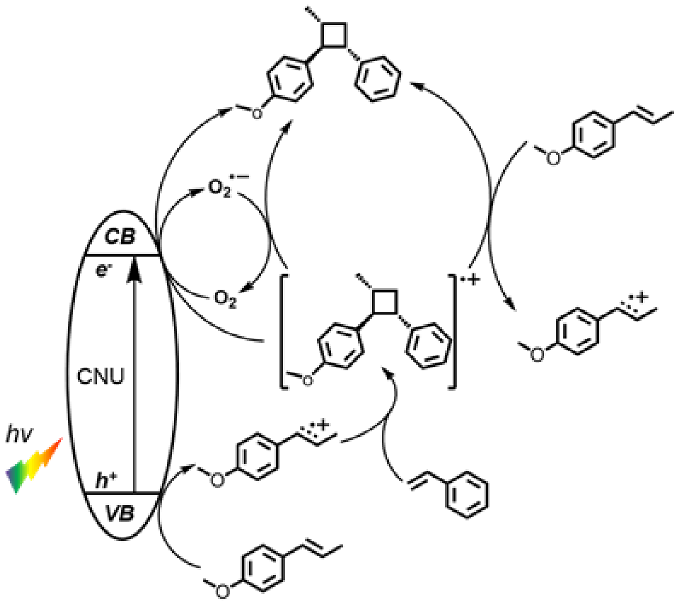

Supplementary Figure 9. Proposed reaction mechanism. Crossed [2+2] cycloaddition catalyzed by UCN under visible light irradiation.

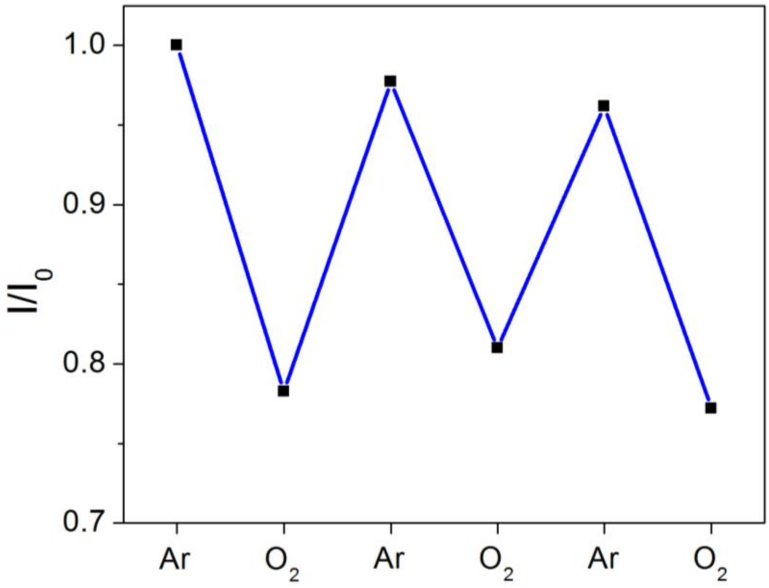

Supplementary Figure 10. Fluorescence emission spectra. The change after bubbling with Ar and O_2 for 5 min, respectively. I_0 for emission intensity after degassing with Ar and I for emission intensity after degassing with O_2.

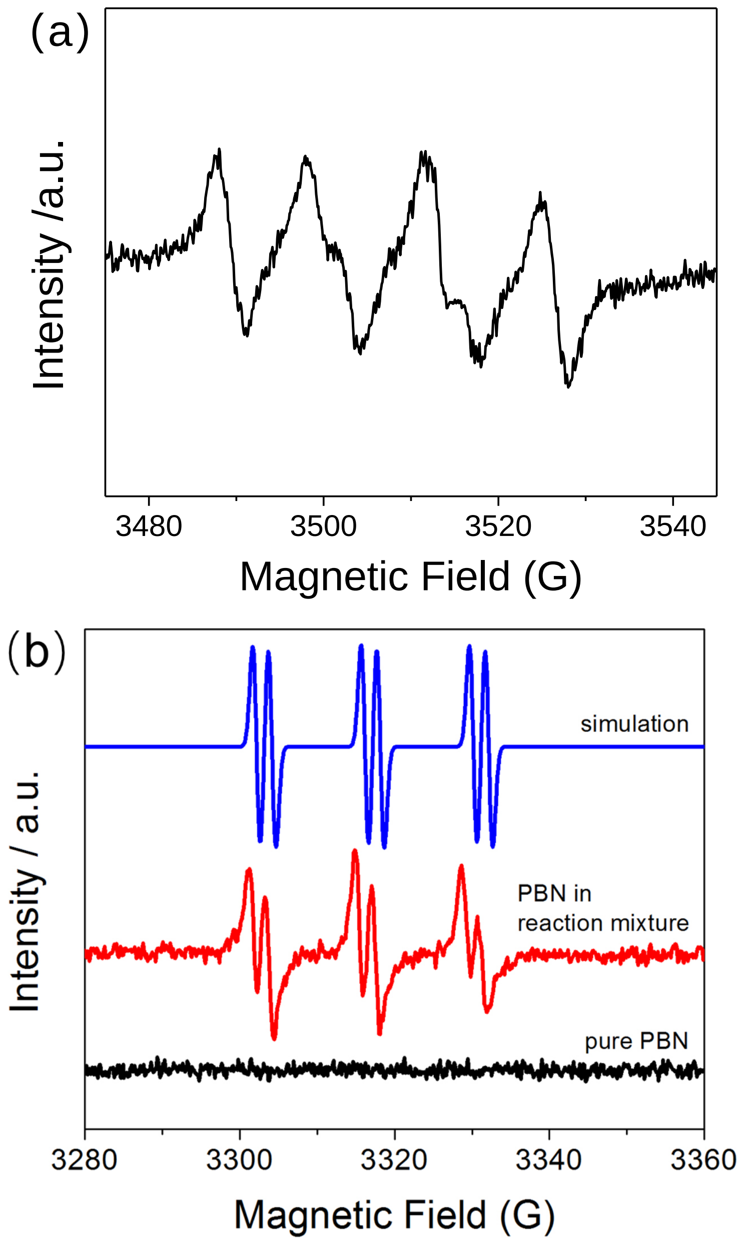

Supplementary Figure 11. Characterization of EPR signals. a DMPO-$O_2{\bullet}^-$ from carbon nitride under visible light irradiation; **b** PBN as a radical trapping agent for anethole radical intermediate under light irradiation. Pure PBN (black trace); PNB in reaction mixture (red trace); and simulated EPR pattern for the trapped radical (blue trace).

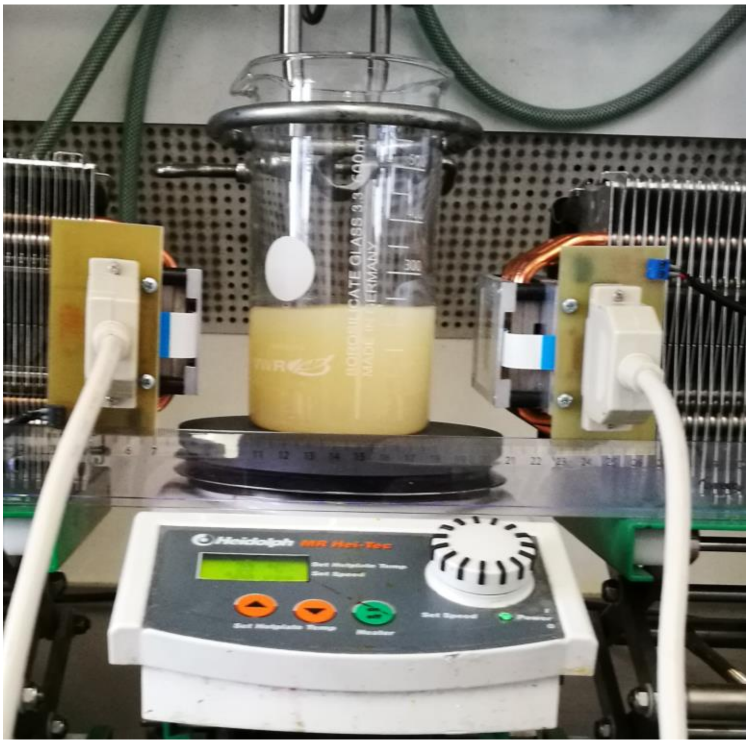

Supplementary Figure 12. Photograph of magnosalin production in 4-gram batch condition.

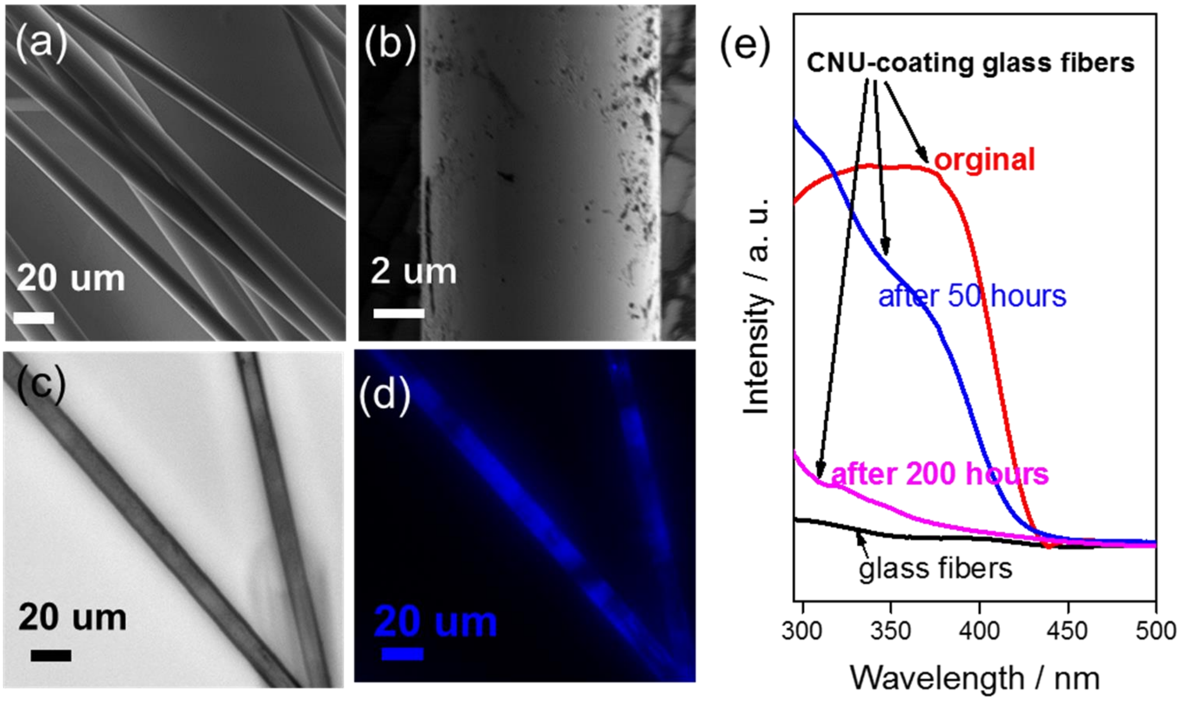

Supplementary Figure 13. SEM images and DRS spectra of UCN@glass fibers. a, b SEM images of UCN coating glass fibers; **c** Optical microscope image; **d** fluorescence image of UCN-coated glass fibers; **e** DRS of commercial glass fibers and UCN-coating glass fibers after different reaction time respecti vely.

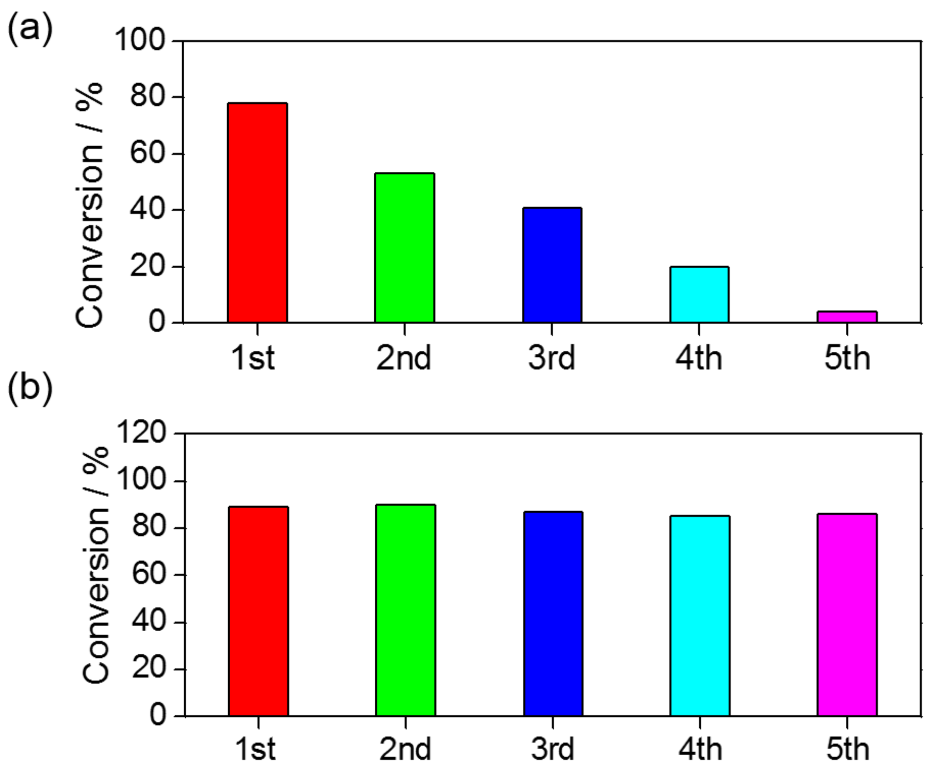

Supplementary Figure 14. The stability test. a UCN-coating glass fibers; **b** UCN-coating glass beads in continuous-flow photoreactor.

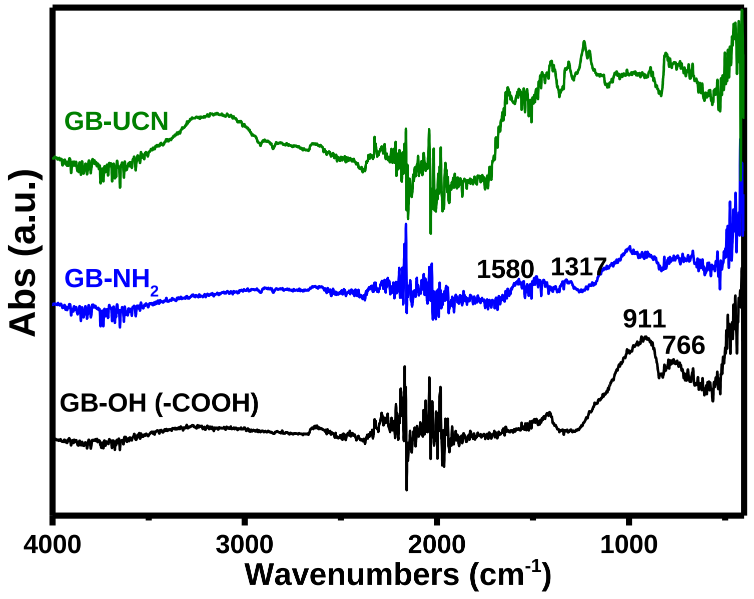

Supplementary Figure 15. IR spectrum of clean GB-OH, NH_2-modifified GB-NH_2 and GB-UCN.

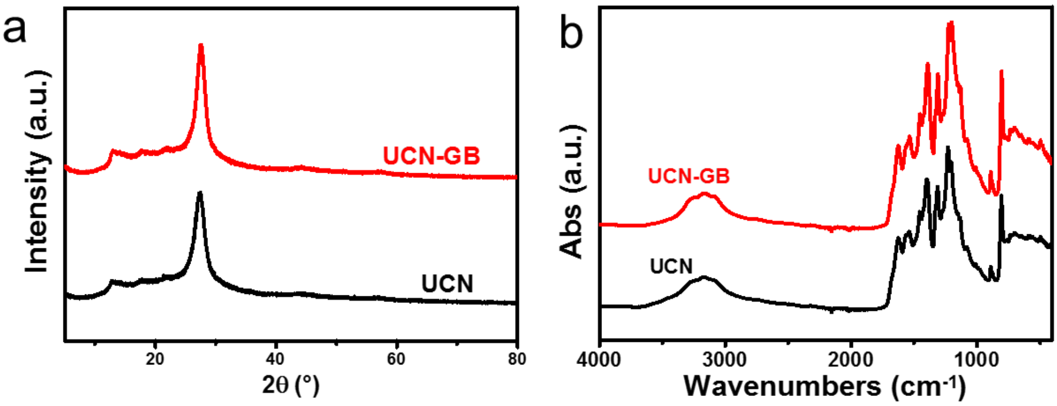

Supplementary Figure 16. Chemical structure characterization. a XRD and **b** IR of UCN powders and UCN coated on the glass beads.

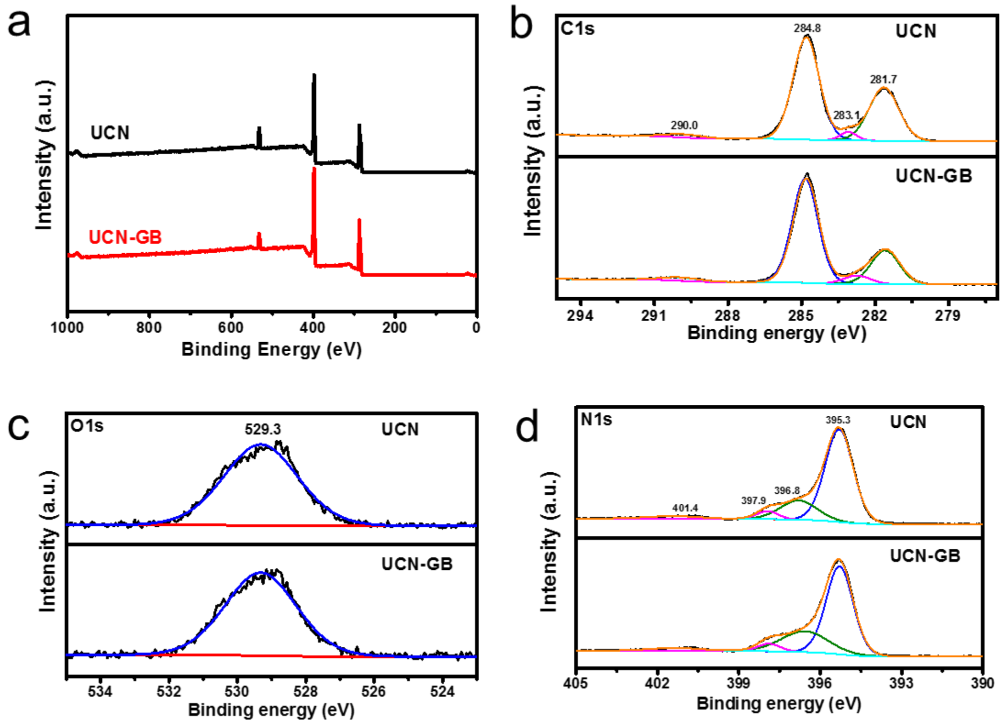

Supplementary Figure 17. XPS spectra of UCN powders and UCN coated on the glass beads. a survey; **b** C1s; **c** N1s; **d** O1s.

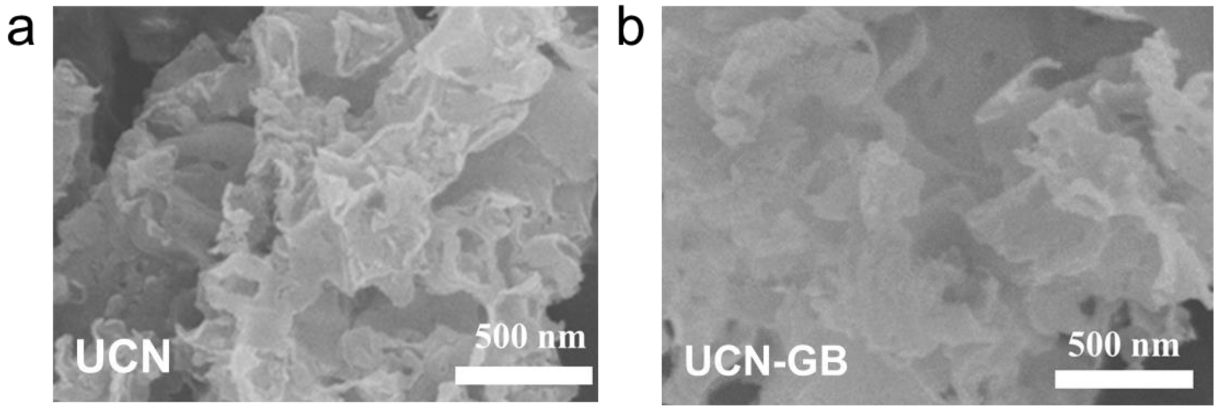

Supplementary Figure 18. SEM images. a UCN; **b** UCN-GB means UCN coated on the glass beads.

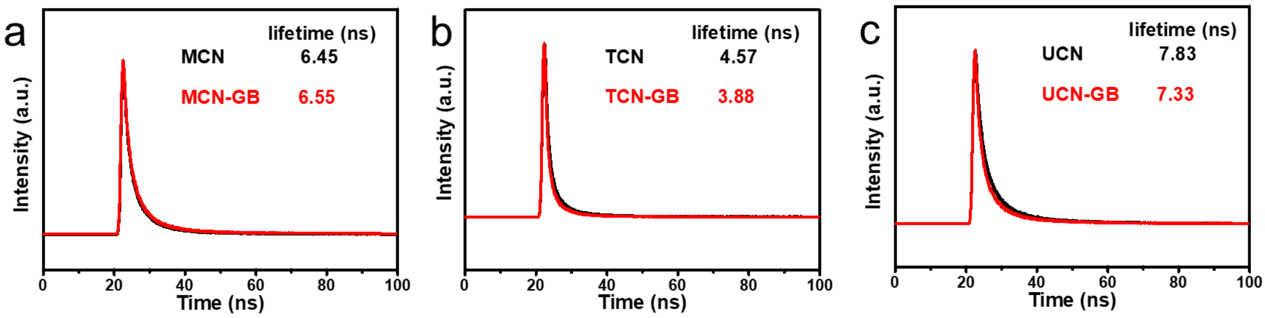

Supplementary Figure 19. Time-resolved photoluminescence spectra. PCNs (black line) and PCN coated on glass beads (red line); **a** PCN from melamine named as MCN; **b** PCN from thiourea named as TCNs; **c** PCN from urea named as UCN.

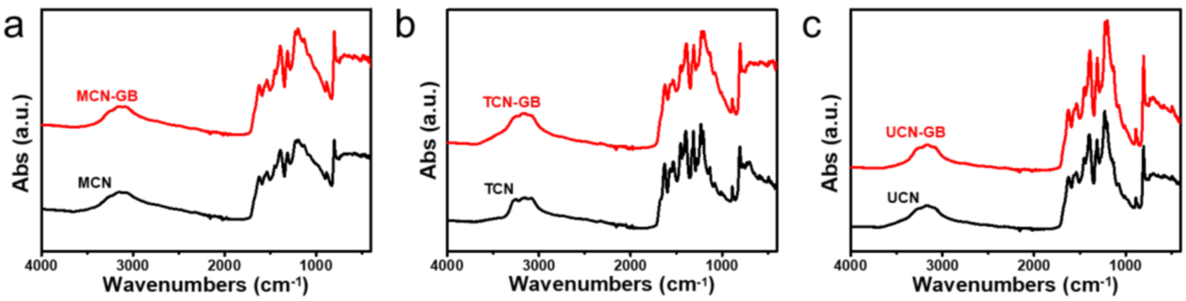

Supplementary Figure 20. Chemical structure characterization. The diffuse reflectance infrared Fourier transform (DRIFT) of PCNs (black line) and PCNs coated on glass beads (red line) from various precursors. (a) PCN from melamine named as MCN; (b) PCN from thiourea named as TCNs; (c) PCN from urea named as UCN.

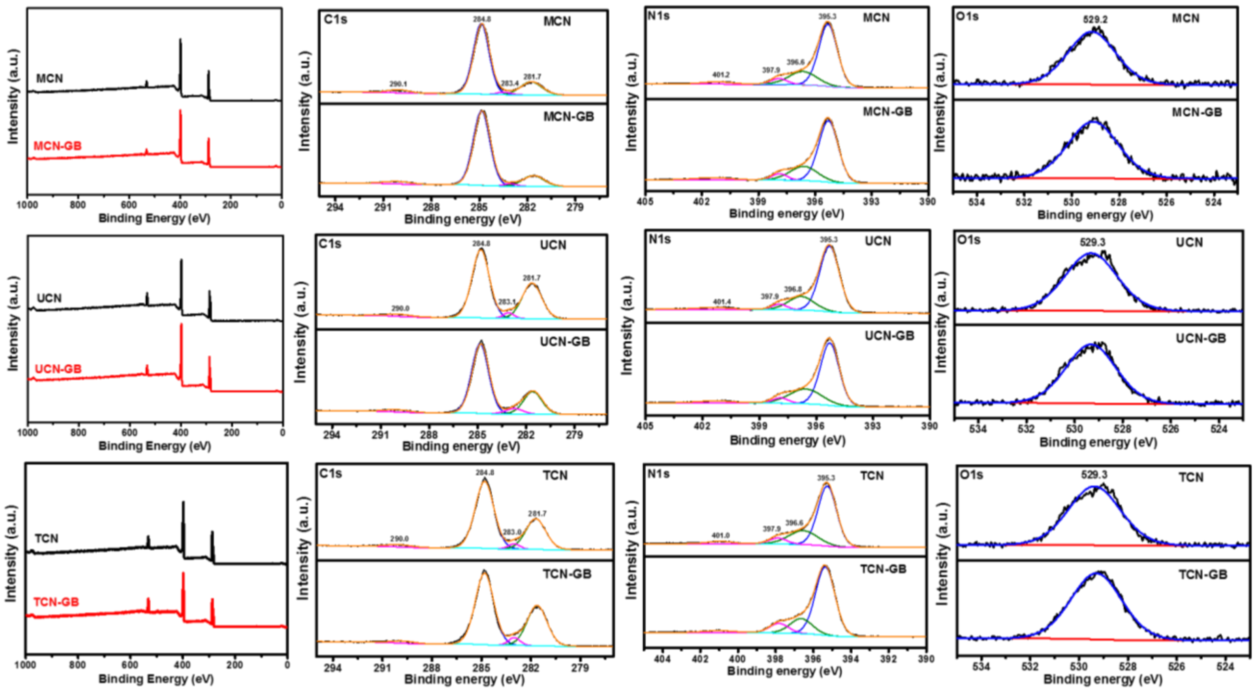

Supplementary Figure 21. The XPS spectra of PCNs and PCN coated on glass beads.

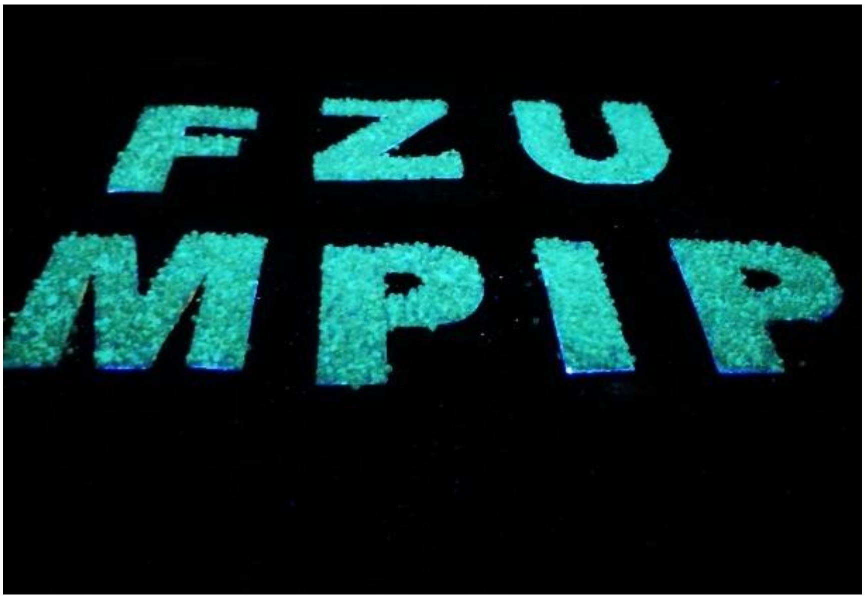

Supplementary Figure 22. Photo of UCN-coated glass beads pattern with words "FZU" and "MPIP" under the irradiation of UV light

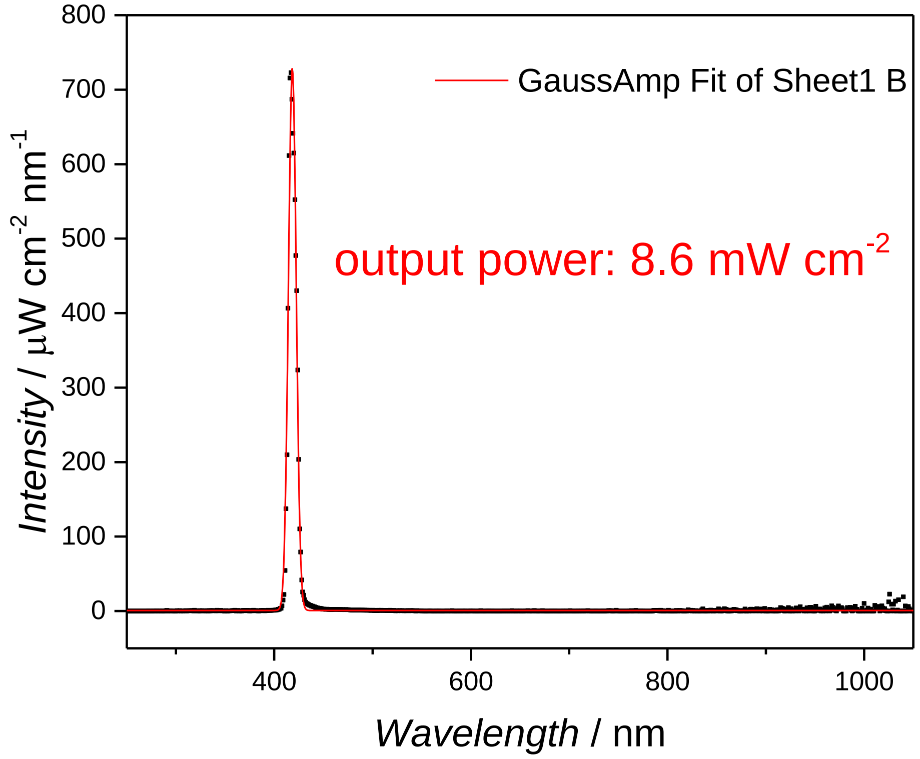

Supplementary Figure 23. Output section of LED with 420 nm band-pass filter. (The integral area means the value of output power: 8.6 mW•cm^{-2})

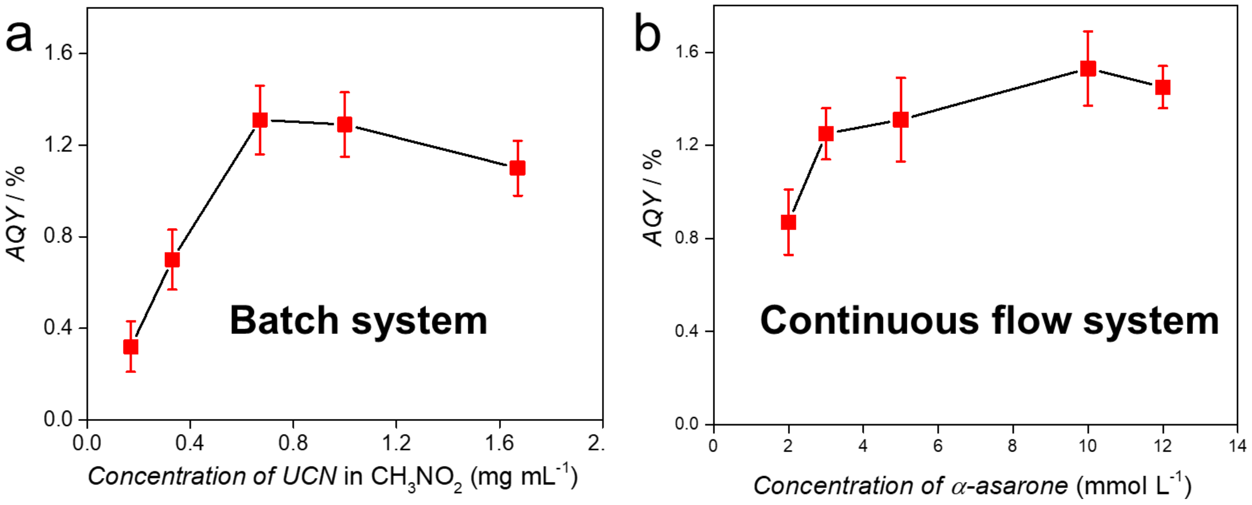

Supplementary Figure 24. Optimized conditions for AQY measurement. a batch system; **b** flow system.

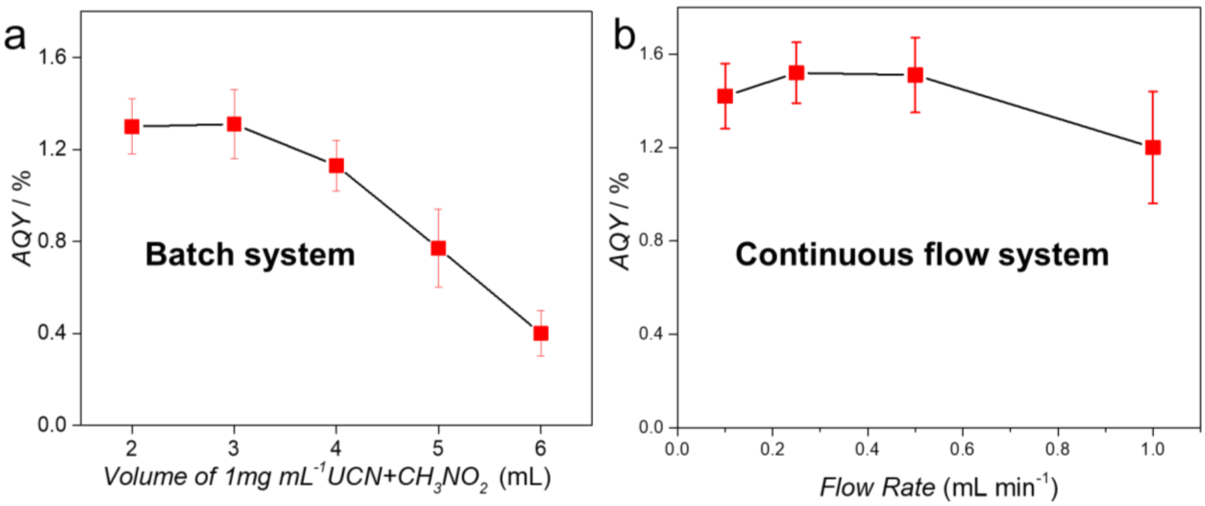

Supplementary Figure 25. AQY measurement. a batch system and **b** continuous flow system.

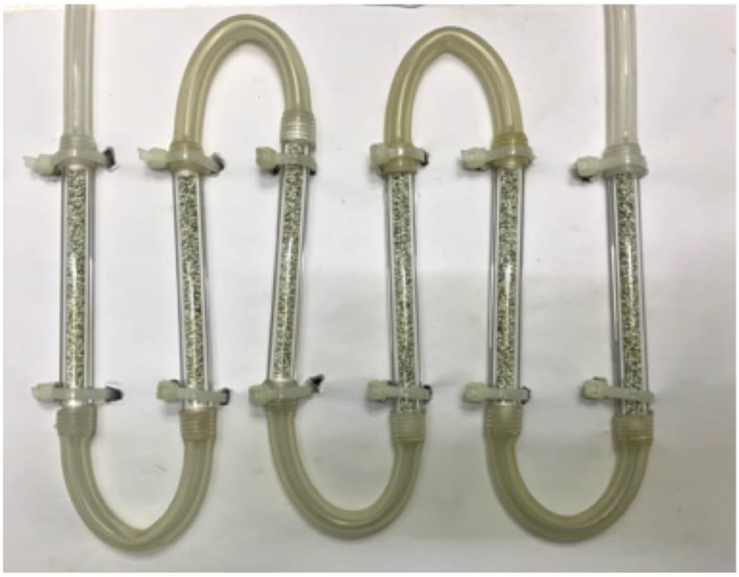

Supplementary Figure 26. Photograph of the continuous flow photoreactor assembled from 6 paralleled glass tubes.

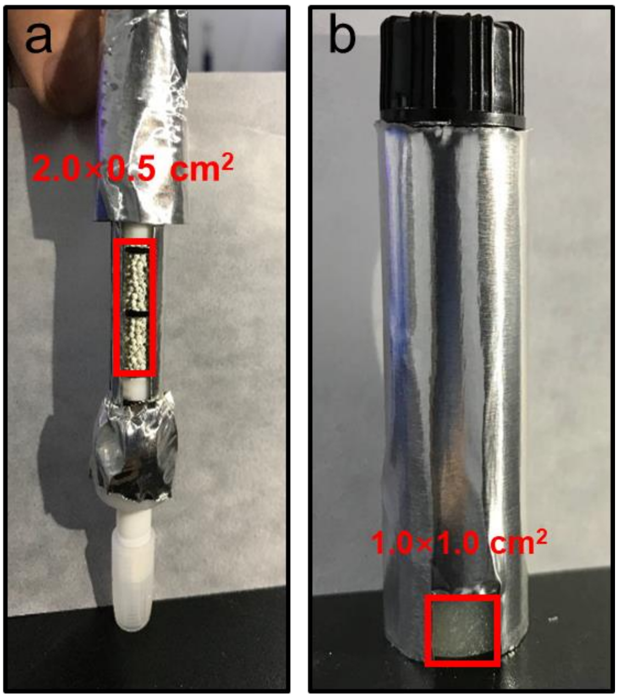

Supplementary Figure 27. Photograph of reactors for AQY measurement. a continuous flow system; **b** batch system.

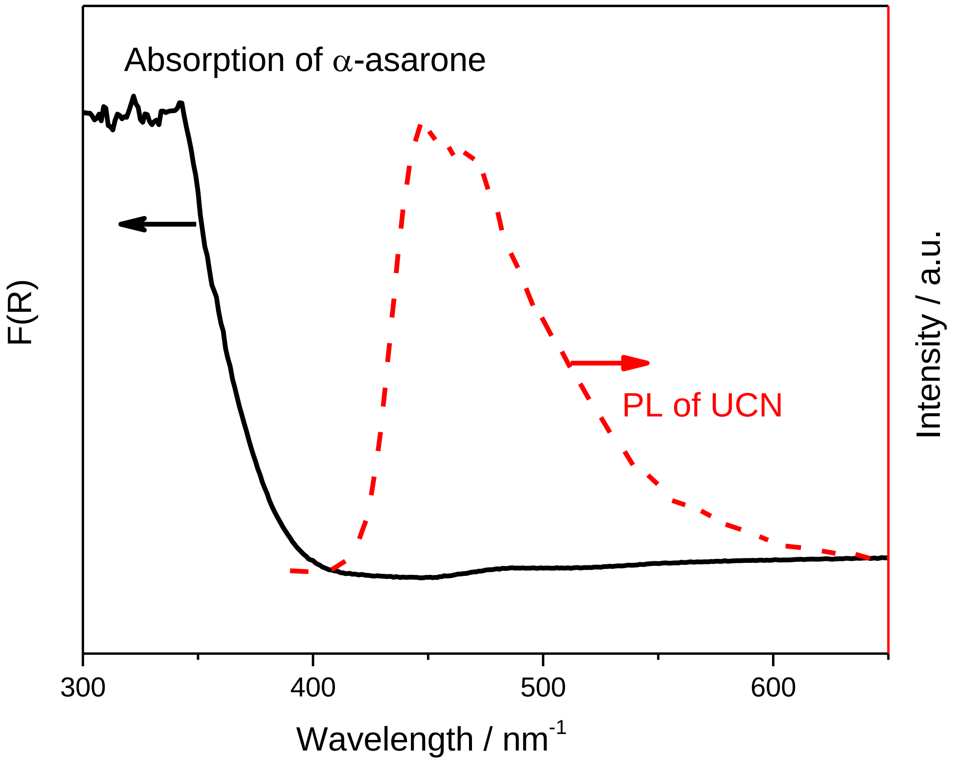

Supplementary Figure 28. Absorption of *α*-asarone and fluorescence spectrum of UCN

Supplementary Table

Supplementary Table 1. Screening and control experiments of UCN catalyzed cross [2+2] cycloaddition between *trans*-anethole and styrene under white light [a]

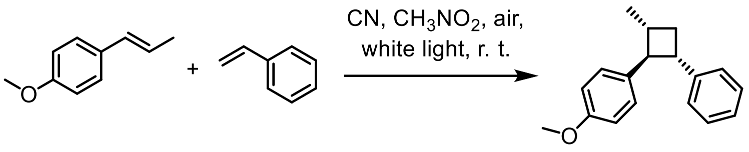

Entry	light	Reaction condition variations	Conversion [b] (%)	Selectivity [b] (%)
1	+	Standard conditions (UCN)	>99	>92
2	+	MCN photocatalyst	83	>90
3	-	TCN photocatalyst	35	>90
4	+	Without photocatalyst	trace	-
5	-	In dark	trace	-
6	+	In acetonitrile	2.5	>92
7	+	In toluene	trace	-
8	+	In dimethyl sulfoxide	trace	-
9	+	In dichloromethane	trace	-
10	+	In DMF	trace	-
11	+	In N_2	6.2	>93
12	+	With hole scavenger [c]	trace	-
13	+	With electron scavenger [d]	53	75

[a] Standard reaction conditions: [anethole] = 0.1 M, [styrene] = 0.5 M, [CN] = 1 mg/mL, CH_3NO_2 2.4 mL, white LED lamp (0.16 W/cm^2), room temperature, air, 8 h. [b] Conversion and selectivity determined by GC-MS, dimer and oxides of alkene-1 as the major side product. [c] KI as a hole scavenger. [d] $K_2S_2O_8$ as an electron scavenger.

Supplementary Discussion

The AQY is related to the reaction conditions including the following factors: output of light, area of irradiation and ratio of catalysts and reactants. We need to optimize conditions of the batch reactor system to get a reliable value that is scientific to compare with that of the flow reactor system. Here, we tested the AQY of the intermolecular addition between α-asarone and styrene and set the irradiation area as 1×1 cm^2, the concentrate of α-asarone as 10 mmol/L and the volume of solvents (CH_3NO_2) as 3 mL to get the optimal value of the catalyst mass. As shown in Supplementary Figure 24a, the AQY increased first with the increase of photocatalyst mass, when the mass of UCN was 2 mg (0.67 mg mL^{-1}), the AQY achieved the maximum 1.31%, whereafter the AQY begun to decrease due to the optical shielding effect, but considering the experimental error (from the variance of three experimental results), we accepted under this condition, the concentration of UCN from 0.67 mg mL^{-1} (2mg UCN) to 1mg mL^{-1} (3mg UCN) could achieve the highest AQY (1.31%). Afterwards, we also used 1mg mL^{-1} UCN in CH_3NO_2 to study the effect of amplification in this condition of batch system. It could be observed that the AQY decreased in Supplementary Figure 25a after the volume increased with 1×1 cm^2 irradiation area, thus resulting from not only the increase of by-productions but also the light shielding effect, which demonstrated the limitation of a dimension-enlarging strategy for scale-up.

Then, we studied the AQY of the flow-reactor system based on the results gotten from the batch-reactor. It was noting that we used residence time of solvent (t_r = volume/flow rate) to replace reaction time (t), furthermore in order to accurately calculate the AQY of the flow-reactor, we calculated the AQY starting at the moment solvents was filled with the transparent tube, and stopping at the moment a certain volume of production was obtained (here we set the volume as 30 mL) in a single path. We filled the photocatalytic reactor with 2g of UCN-coating GBs, controlled the irradiation area with 2×0.5 cm^2, set the flow rate as 0.5 mL min^{-1} and changed the concentrate of substrate to study the ability of photocatalytic conversion. In Supplementary Figure 24b, the AQY of photocatalytic reaction would increase firstly as the increase of the substrate concentration at a low-value field and the highest AQY was 1.53% when the concentration was 10 mmol L^{-1}. When the concentration exceeded 10 mmol L^{-1}, the AQY begun to decreased. Based on this result, we studied the relationship between AQY and Flow Rate that was shown in Supplementary Figure 25b. There was only a little difference among AQYs corresponding to the different flow rates (0.1, 0.25 and 0.5 mL min^{-1}) with the concentration of 10 mmol L^{-1}.

Supplementary Methods

General Information. Urea, thiourea, melamine, glass fiber and glass beads were obtained from commercial source. *α*-asarone was purchased from TCI Development Co., Ltd. *E*-anethole, *E*-methoxycinnamyl alcohol, *p*-methoxycinnamaldehyde and *p*-methoxycinnamic acid were obtained from commercial source without further purification. Styrene derivatives, such as methyl-styrene, halogenated styrene, and acetated-styrene, were purified by elution through neutral Al_2O_3 (50-200 um) and anhydrous $CaCl_2$ with a ratio of 95/5. Other chemicals and solvents were used as received unless otherwise noted. Column flash chromatography was carried out with silica 60 (0.063-0.2 mm).

Characterization. XRD measurements were performed on a Bruker D8 Advance diffractometer with $Cu_{K\alpha1}$ radiation (λ=1.5406 Å). Fourier transform infrared (FTIR) spectra were recorded on Varian 1000 FT-IR spectrometer. The ^{1}H and ^{13}C NMR experiments were performed on a Bruker Advance 300 MR (at 300 MHz) spectrometer. Solid State ^{13}C CP/MAS NMR measurements were carried out with Bruker Avance II solid state NMR spectrometer operating at 300 MHz Larmor frequency equipped with a standard 4 mm magic angle spinning (MAS) double resonance probe head. The UV/Vis diffuse reflectance spectra (DRS) were recorded on a Perkin Elmer Lambda 100 spectrometer. Photoluminescence (PL) spectra were recorded on J&M TIDAS spectrofluormeter. GC-MS detection was carried out on Shimadzu GC-2010 plus gas chromatography and QP2010 ultra mass spectrometer with fused silica column (122-5532, DB-5MS) and flame ionization detector. Surface areas and pore size distributions were conducted by N2 adsorption and desorption at 77.3 K by 2020. Electron paramagnetic resonance (EPR) measurements were carried out on a Bruker EMX-plua spectrometer (Bruker B-VT 2000). Electrochemical measurements were conducted with a Bio-Logic Electrochemical System in a conventional three electrode cell, using a Pt plate as the counter electrode and an Ag/AgCl electrode (3 M KCl) as the reference electrode, the active area was confined to 0.25 cm^2. Transmission electron microscopy (TEM) was performed on a JEOL JEM-1400 microscope. scanning electron microscope (SEM) was recorded by a LEO Gemini 1530 (Germany) microscope with an in lens SE detector. Measurement of Atomic Force Microscope (AFM) was conducted on Bruker Dimension Icon. Optical microscope and fluorescence microscope images were taken by Leica DMI8.

Details of the calculations. All DFT calculations were carried out via the Vienna ab initio simulation package (VASP).[1, 2] Projector augmented wave (PAW) potentials[3, 4] were used to describe the electron-ion interactions and the generalized gradient approximation of Perdew, Burke, and Ernzerhof (GGA-PBE) functional was used to describe the exchange and correlation interactions.[5] Grimme's D2 method was included for the van del Waals corrections.[6] The wave function was expanded using plane waves with a cutoff energy of 520 eV. The Brillouin zone of 2 × 2 unit cells was sampled with a 5×5×1 Monkhorst−Pack mesh of k points.[7] The systems were fully relaxed until the conventional energy smaller than

10^{-4} eV and the Hellmann−Feynman forces acting on the atoms were smaller than 0.01 eV/Å. The 1.5 nm vacuum layer was added to avert the interaction between adjacent layers.

Details of AQY measurement: A white light LED with a 420 ± 4.6 nm band-pass filter (Output: 8.6 mW/cm^2; the output section was shown in Supplementary Fig. 20) was employed to test the AQY. The irradiation area was controlled as 1×1 cm^2 for the batch reactor and 0.5×2 cm^2 for the flow reactor respectively as shown in Supplementary Fig. 27. Depending on the amount of production from the photocatalytic [2+2] cycloaddition reaction in an average of one hour, and this reaction was regarded as a single electron process (Supplementary Fig. 28). AQY was calculated as Supplementary Equations 1:

$$AQY\% = N_e/N_p \times 100\% = (n \cdot N_A) / (E_{total}/E_{photon})$$

$$= (n \cdot N_A \cdot \hbar \cdot C) / (S \cdot P \cdot \lambda) \times 100\% \qquad \text{Supplementary Equations (1)}$$

Where, N_e is the number of electron obtained by aimed product; N_p is the total number of incident photon. n is the amount of aimed product (mol) without no by-production; N_A is Avogadro constant (6.022×10^{23}/mol); $\hbar$ is the Planck constant (6.626×10^{-34} J·s); C is the speed of light (3×10^8 m/s); S is the irradiation area (cm^2); P is the intensity of irradiation light (W/cm^2); λ is the wavelength of the monochromatic light (m).

Supplementary References

[1] Kresse, G. & Furthmuller, J. Efficient iterative schemes for ab initio total-energy calculations using a plane-wave basis set. *Phys. Rev. B*, **54**, 11169-11186 (1996);
[2] Kresse, G. & Furthmuller J. Efficiency of ab-initio total energy calculations for metals and semiconductors using a plane-wave basis set, *Computational Mater. Sci*., **6**, 15-50 (1996).
[3] Blochl, P. E. Projector augmented-wave method, *Phys. Rev. B*, **50**, 17953-17979 (1994);
[4] Kresse, G. & Joubert D. From ultrasoft pseudopotentials to the projector augmented-wave method, *Phys. Rev. B*, **59**, 1758-1775 (1999).
[5] Perdew J. P., Burke K., Ernzerhof M., Generalized Gradient Approximation Made Simple. *Phys. Rev. Lett.*, **77**, 3865-3868 (1996).
[6] Grimme S., Semiempirical GGA-type density functional constructed with a long-range dispersion correction, J. Comput. Chem., **27**, 1787-1799 (2006).
[7] Pack H., Monkhorst J., "Special points for Brillouin-zone integrations"—a reply. *Phys. Rev. B*, **16**, 1748-1749 (1977).
