# Supplementary material for: Heterogeneous photoredox flow chemistry for the scalable organosynthesis of fine chemicals
Source: Nat Commun. 2020 Mar 6;11:1239. doi: 10.1038/s41467-020-14983-w (PMC7060272; doi:10.1038/s41467-020-14983-w)
Supplement: Supplementary file 2 — Description of Additional Supplementary Files [file 41467_2020_14983_MOESM2_ESM.pdf]

Description of Additional Supplementary files

File name: Supplementary Data 1

Description: Crystal data for Magnosalin and NMR characterization of Compounds with their corresponding spectra.
