# Supplementary material for: Heterogeneous photoredox flow chemistry for the scalable organosynthesis of fine chemicals
Source: Nat Commun. 2020 Mar 6;11:1239. doi: 10.1038/s41467-020-14983-w (PMC7060272; doi:10.1038/s41467-020-14983-w)

Crystal data for Magnosalin

formula	$C_{24}H_{32}O_6$
molecular weight	416.49 $gmol^{-1}$
absorption	$\mu = 0.087\ mm^{-1}$
crystal size	0.1 x 0.11 x 0.50 mm^3 colourless block
space group	$P\ 2_1/n$ (monoclinic)
lattice parameters	a = 5.6941(3)Å
(calculate from	b = 17.8408(8)Å ß = 92.165(4)°
8017 reflections with	c = 22.3112(12)Å
2.15° < θ < 28.18°)	V = 2264.9(2)$Å^3$ z = 4 F(000) = 896
temperature	-80°C
density	$d_{xray} = 1.221\ gcm^{-3}$

<u>data collection</u>

diffractometer	STOE IPDS 2T
radiation	Mo-K_α Graphitmonochromator
Scan – type	ω scans
Scan – width	1°
scan range	$2° \leq \theta < 28°$
	$-7 \leq h \leq 7$ $-23 \leq k \leq 23$ $-29 \leq l \leq 29$
number of reflections:	
measured	11466
unique	5574 ($R_{int} = 0.044$)
observed	3060 ($\|F\|/\sigma(F) > 4.0$)

<u>data correction, structure solution and refinement</u>

corrections	Lorentz and polarisation correction.
Structure solution	Program: SIR-2004 (Direct methods)
refinement	Program: SHELXL-2014 (full matrix). 279 refined parameters, weighting scheme: $w=1/[\sigma^2(F_o^2) + (0.0427*P)^2+1.58*P]$ with $(Max(F_o^2,0)+2*F_c^2)/3$. H-atoms at calculated positions and refined with isotropic displacement parameters, non H-atoms refined anisotropically.
R-values	wR2 = 0.1680 (R1 = 0.0684 for observed reflections, 0.1427 for all reflections)
goodness of fit	S = 1.089
maximum deviation of parameters	0.001 * e.s.d
maximum peak height in diff. Fourier synthesis	0.23, -0.24 $eÅ^{-3}$

final coordinates and equivalent displacement parameters (Å^2)

$U_{äq} = (1/3)*\sum\sum_{ij} a_i^* a_j^* \mathbf{a}_i \mathbf{a}_j$

Atom	X	Y	Z	U_{eq}
C1	0.2559(5)	0.2154(1)	0.4732(1)	0.0392(8)
C2	0.1460(5)	0.1353(2)	0.4728(1)	0.0459(9)
C3	0.0590(5)	0.1509(2)	0.4081(1)	0.0438(9)
C4	0.0643(5)	0.2354(1)	0.4246(1)	0.0392(8)
C5	0.2833(5)	0.2602(1)	0.5301(1)	0.0383(8)
C6	0.1292(5)	0.3187(1)	0.5427(1)	0.0403(8)
C7	0.1546(5)	0.3604(1)	0.5952(1)	0.0409(8)
C8	0.3401(5)	0.3450(2)	0.6357(1)	0.0423(8)
C9	0.4934(5)	0.2867(2)	0.6244(1)	0.0438(9)
C10	0.4642(5)	0.2448(2)	0.5720(1)	0.0407(8)
O11	0.0094(3)	0.4183(1)	0.61098(9)	0.0512(7)
C12	-0.1657(5)	0.4407(2)	0.5674(1)	0.055(1)
O13	0.3563(4)	0.3901(1)	0.68557(8)	0.0544(7)
C14	0.5659(6)	0.3840(2)	0.7229(1)	0.065(1)
O15	0.6102(3)	0.1863(1)	0.55829(8)	0.0514(7)
C16	0.7631(6)	0.1584(2)	0.6053(1)	0.061(1)
C17	0.2917(6)	0.0661(2)	0.4870(2)	0.060(1)
C18	-0.1675(6)	0.1164(2)	0.3835(2)	0.061(1)
C19	0.0974(4)	0.2940(1)	0.3774(1)	0.0381(8)
C20	0.2679(4)	0.2860(1)	0.3343(1)	0.0388(8)
C21	0.2900(5)	0.3368(2)	0.2882(1)	0.0428(8)
C22	0.1354(5)	0.3978(2)	0.2842(1)	0.0449(9)
C23	-0.0293(5)	0.4085(2)	0.3271(1)	0.0450(9)
C24	-0.0491(5)	0.3569(2)	0.3737(1)	0.0417(8)
O25	0.4533(4)	0.3326(1)	0.24453(8)	0.0561(7)
C26	0.6111(5)	0.2710(2)	0.2471(1)	0.0529(10)
O27	0.1642(4)	0.4450(1)	0.23628(8)	0.0607(8)
C28	-0.0258(7)	0.4944(2)	0.2213(2)	0.075(1)
O29	-0.2105(4)	0.3640(1)	0.41760(9)	0.0558(7)
C30	-0.3803(5)	0.4223(2)	0.4127(1)	0.055(1)

anisotropic displacement parameters

Atom	U_{11}	U_{22}	U_{33}	U_{12}	U_{13}	U_{23}
C1	0.045(1)	0.030(1)	0.042(1)	0.008(1)	0.004(1)	0.001(1)
C2	0.051(2)	0.033(1)	0.055(2)	0.004(1)	0.012(1)	0.001(1)
C3	0.043(1)	0.034(1)	0.054(2)	0.001(1)	0.005(1)	-0.011(1)
C4	0.041(1)	0.033(1)	0.043(1)	0.005(1)	0.003(1)	-0.006(1)
C5	0.045(1)	0.031(1)	0.039(1)	0.005(1)	0.006(1)	0.000(1)
C6	0.042(1)	0.035(1)	0.044(1)	0.005(1)	0.001(1)	0.000(1)
C7	0.045(1)	0.032(1)	0.047(1)	0.004(1)	0.010(1)	0.000(1)
C8	0.049(2)	0.039(1)	0.039(1)	-0.002(1)	0.006(1)	-0.004(1)
C9	0.048(2)	0.048(2)	0.036(1)	0.006(1)	0.005(1)	0.003(1)
C10	0.044(1)	0.039(1)	0.040(1)	0.007(1)	0.008(1)	0.003(1)
O11	0.057(1)	0.039(1)	0.057(1)	0.0129(9)	0.0008(9)	-0.0102(9)
C12	0.056(2)	0.044(2)	0.064(2)	0.013(1)	0.000(1)	-0.005(1)
O13	0.061(1)	0.056(1)	0.047(1)	0.005(1)	-0.0014(9)	-0.0114(9)
C14	0.065(2)	0.078(2)	0.049(2)	0.000(2)	-0.008(2)	-0.017(2)
O15	0.058(1)	0.057(1)	0.0383(9)	0.024(1)	-0.0005(9)	0.0017(9)
C16	0.077(2)	0.064(2)	0.043(2)	0.030(2)	-0.001(1)	0.008(1)
C17	0.069(2)	0.037(2)	0.074(2)	0.007(1)	0.013(2)	0.008(1)
C18	0.058(2)	0.047(2)	0.078(2)	-0.007(1)	0.001(2)	-0.014(2)
C19	0.038(1)	0.035(1)	0.041(1)	0.006(1)	-0.008(1)	-0.008(1)
C20	0.042(1)	0.035(1)	0.038(1)	0.007(1)	-0.006(1)	-0.006(1)
C21	0.053(2)	0.041(1)	0.034(1)	0.006(1)	-0.006(1)	-0.004(1)
C22	0.061(2)	0.037(1)	0.036(1)	0.008(1)	-0.012(1)	-0.001(1)
C23	0.053(2)	0.036(1)	0.045(1)	0.012(1)	-0.013(1)	-0.007(1)
C24	0.041(1)	0.041(1)	0.043(1)	0.009(1)	-0.007(1)	-0.008(1)
O25	0.070(1)	0.056(1)	0.042(1)	0.022(1)	0.0071(10)	0.0052(9)
C26	0.056(2)	0.051(2)	0.052(2)	0.014(1)	0.005(1)	-0.004(1)
O27	0.087(2)	0.049(1)	0.046(1)	0.023(1)	-0.006(1)	0.0072(9)
C28	0.095(3)	0.061(2)	0.066(2)	0.024(2)	-0.019(2)	0.017(2)
O29	0.054(1)	0.051(1)	0.063(1)	0.0243(10)	0.0080(10)	0.0030(10)
C30	0.046(2)	0.046(2)	0.072(2)	0.016(1)	-0.006(1)	-0.013(1)

final coordinates and isotropic displacement parameters (Å^2) for H- atoms

Atom	X	Y	Z	U_{iso}
H1	0.41142	0.21303	0.45397	0.0471
H2	0.00768	0.13543	0.49913	0.0551
H3	0.18804	0.13985	0.38024	0.0525
H4	-0.08375	0.24733	0.44537	0.0470
H6	0.00435	0.33018	0.51480	0.0484
H9	0.61804	0.27529	0.65246	0.0526
H12A	-0.09061	0.45508	0.53035	0.082
H12B	-0.25305	0.48353	0.58274	0.082
H12C	-0.27409	0.39895	0.55925	0.082
H14A	0.70367	0.39037	0.69833	0.097
H14B	0.57153	0.33455	0.74197	0.097
H14C	0.56588	0.42299	0.75384	0.097
H16A	0.83368	0.11118	0.59258	0.092
H16B	0.67323	0.14976	0.64122	0.092
H16C	0.88745	0.19515	0.61426	0.092
H17A	0.20024	0.02124	0.47623	0.090
H17B	0.33431	0.06508	0.52994	0.090
H17C	0.43474	0.06729	0.46393	0.090
H18A	-0.20212	0.13554	0.34291	0.092
H18B	-0.29615	0.12963	0.40943	0.092
H18C	-0.15078	0.06179	0.38207	0.092
H20	0.37167	0.24435	0.33690	0.0465
H23	-0.12962	0.45100	0.32498	0.0541
H26A	0.52203	0.22407	0.24362	0.0794
H26B	0.70085	0.27176	0.28549	0.0794
H26C	0.71922	0.27457	0.21412	0.0794
H28A	0.00647	0.52101	0.18406	0.112
H28B	-0.04332	0.53077	0.25374	0.112
H28C	-0.17117	0.46542	0.21575	0.112
H30A	-0.30066	0.47101	0.41471	0.082
H30B	-0.48861	0.41829	0.44560	0.082
H30C	-0.46853	0.41790	0.37425	0.082

Compound Characterization

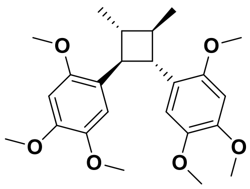

5,5'-((1S,2S,3R)-3-methylcyclobutane-1,2-diyl)bis(1,2,4-trimethoxybenzene): The synthesis (2g α-asarone) was completed in flow-continuous reactor. After the reaction was completed, the mixture was performed as the workup protocol and purified through chromatography on silica with hexane/diethyl ether (3/1) as the elution to afford the pure cyclobutane as white solid.

R_f (hexane/diethyl ether 3/1): 0.07

^{1}H NMR (300 MHz, $CDCl_3$) δ 6.87 (s, 2H), 6.39 (s, 2H), 3.80 (s, 6H), 3.78 (s, 6H), 3.62 (s, 6H), 3.21 (dd, J = 3.3, 9.0 Hz, 2H), 1.70 (m, 2H), 1.12 (d, J = 5.2 Hz, 6H).

^{13}C NMR (300 MHz, $CDCl_3$) δ 151.70, 147.56, 143.15, 123.97, 112.33, 97.96, 56.77, 56.59, 56.23, 45.41, 43.49, 19.09.

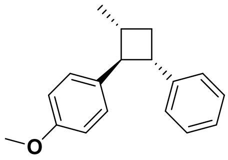

1-methoxy-4-((1S,2R,4S)-2-methyl-4-phenylcyclobutyl) benzene: The synthesis (0.75 g *E*-anethole, 6 mL styrene) was completed in flow-continuous reactor. After the reaction was completed, the mixture was performed as the workup protocol and purified through chromatography on silica with hexane/ dichloromethane (6/1) as the elution to afford the pure cyclobutane as clear oil.

R_f (hexane/dichloromethane 6/1): 0.24

^{1}H NMR (300 MHz, $CDCl_3$): δ 7.18 (m, 2H), 7.10 (m, 5H), 6.78 (BB’ of AA’BB’, J = 8.7 Hz, 2H), 3.29 (s, 3H), 3.32 (q, J = 8.1 Hz, 1H), 2.86 (t, J = 9.6 Hz, 1H), 2.45 (dt, J = 2.1, 7.8 Hz, 1H), 2.24 (m, 1H), 1.63 (q, J = 9.9 Hz, 1H), 1.11 (d, J = 6.6 Hz 3H).

^{13}C NMR (300 MHz, $CDCl_3$): δ 158.11, 144.68, 135.87, 128.26, 127.80, 126.64, 125.94, 113.80, 55.59, 55.27, 44.15, 35.53, 33.98, 20.50.

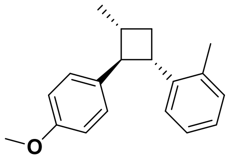

1-((1S,2S,3R)-2-(4-methoxyphenyl)-3-methylcyclobutyl)-2-methylbenzene: The synthesis (0.75 g *E*-anethole, 6.5 mL 2-methylstyrene) was completed in flow-continuous reactor. After the reaction was completed, the mixture was performed as the workup protocol and purified through chromatography on silica with hexane/ dichloromethane (6/1) as the elution to afford the pure cyclobutane as clear oil.

R_f (hexane/dichloromethane 6/1): 0.30

^{1}H NMR (300 MHz, $CDCl_3$): δ 7.23 (d, J = 7.5 Hz, 1H), 7.11 (AA' of AA'BB', J = 8.4 Hz, 2H), 7.07 (m, 1H), 7.00 (m, 2H), 6.73 (BB' of AA'BB', J = 8.6 Hz, 2H), 3.67 (s, 3H), 3.45 (q, J = 8.1 Hz, 1H), 3.04 (t, J = 9.6 Hz, 1H), 2.52 (q, J = 10.2 Hz, 1H), 2.24 (m, 1H), 2.10 (s, 3H), 1.47 (q, J = 9.9 Hz, 1H), 1.12 (d, J = 6.3 Hz, 3H).

^{13}C NMR (300 MHz, $CDCl_3$): δ 158.13, 142.36, 136.09, 136.00, 130.02, 127.81, 125.94, 125.87, 125.78, 113.80, 55.28, 53.44, 41.78, 35.73, 34.77, 20.66, 19.88.

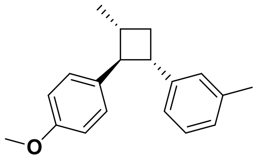

1-((1S,2S,3R)-2-(4-methoxyphenyl)-3-methylcyclobutyl)-3-methylbenzene: The synthesis (0.75 g *E*-anethole, 6.5 mL 3-methylstyrene) was completed in flow-continuous reactor.. After the reaction was completed, the mixture was performed as the workup protocol and purified through chromatography on silica with hexane/dichloromethane (10/1) as the elution to afford the pure cycloadduct as clear oil.

R_f (hexane/dichloromethane 10/1): 0.23

^{1}H NMR (300 MHz, $CDCl_3$): δ 7.09 (m, 3H), 6.92 (m, 3H), 6.76 (BB' of AA'BB', J = 8.7 Hz, 2H), 3.68 (s, 3H), 3.27 (q, J = 8.1 Hz, 1H), 2.86 (t, J = 9.6 Hz, 1H), 2.42 (dt, J = 2.1, 9.9 Hz, 1H), 2.24 (m, 1H), 2.22 (s, 3H), 1.61 (q, J = 10.2 Hz, 1H), 1.10 (d, J = 6.6 Hz, 3H).

^{13}C NMR (300 MHz, $CDCl_3$): δ 158.12, 144.67, 137.79, 136.01, 128.21, 127.81, 127.44, 126.75, 123.77, 113.82, 55.48, 55.28, 44.12, 35.55, 34.16, 21.53, 20.56.

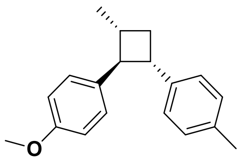

1-methoxy-4-((1S,2R,4S)-2-methyl-4-(p-tolyl)cyclobutyl)benzene: The synthesis (0.75 g *E*-anethole, 6.5 mL 4-methylstyrene) was completed in flow-continuous reactor. After the reaction was completed, the mixture was performed as the workup protocol and purified through chromatography on silica with hexane/dichloromethane (6/1) as the elution to afford the pure cycloadduct as clear oil.

R_f (hexane/dichloromethane 6/1): 0.35

^1H NMR (300 MHz, $CDCl_3$): δ 7.06 (AA' of AA'BB', J = 8.7 Hz, 2H), 7.00 (s, 4H), 6.77 (BB' of AA'BB', J = 8.4 Hz, 2H), 3.70 (s, 3H), 3.25 (q, J = 7.8 Hz, 1H), 2.84 (t, J = 9.6 Hz, 1H), 2.43 (q, J = 9.9 Hz, 1H), 2.25 (m, 1H), 2.22 (s, 3H), 1.61 (q, J = 10.2 Hz, 1H), 1.09 (d, J = 6.3 Hz, 3H).

^{13}C NMR (300 MHz, $CDCl_3$): δ 158.06, 141.67, 135.98, 135.39, 128.93, 127.77, 126.55, 113.76, 55.64, 55.26, 43.89, 35.40, 34.14, 21.03, 20.52.

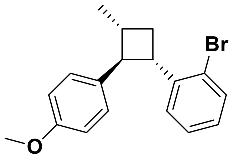

1-bromo-2-((1S,2S,3R)-2-(4-methoxyphenyl)-3-methylcyclobutyl)benzene: The synthesis (0.75 g *E*-anethole, 6 mL 1-bromo-2-vinylbenzene) was completed in flow-continuous reactor. After the reaction was completed, the mixture was performed as the workup protocol and purified through chromatography on silica with hexane/ dichloromethane (6/1) as the elution to afford the pure cyclobutane as clear oil.

R_f (hexane/dichloromethane 6/1): 0.35

^1H NMR (300 MHz, $CDCl_3$): δ 7.54 (dd, J = 1.2, 6.6 Hz, 1H), 7.40 (dd, J =1.5, 6.3 Hz, 1H), 7.30 (m, 1H), 7.26 (AA' of AA'BB', J = 8.7 Hz, 2H), 7.08 (dt, J = 1.5, 7.8 Hz, 1H), 6.91 (BB' of AA'BB', J = 8.7 Hz, 2H), 3.82 (s, 3H), 3.75 (q, J = 9.9 Hz, 1H), 3.19

(t, J = 9.6 Hz, 1H), 2.83 (m, 1H), 2.37 (m, 1H), 1.53 (q, J = 9.9 Hz, 1H), 1.27 (d, J = 6.6 Hz, 3H).

^{13}C NMR (300 MHz, $CDCl_3$): δ 158.19, 143.18, 135.29, 132.71, 127.80, 127.80, 127.49, 127.40, 124.19, 113.82, 55.27, 52.84, 43.55, 35.79, 35.40, 20.62.

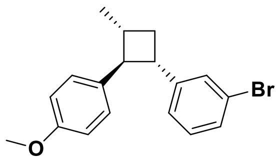

1-bromo-3-((1S,2S,3R)-2-(4-methoxyphenyl)-3-methylcyclobutyl)benzene: The synthesis (0.75 g *E*-anethole, 6 mL 1-bromo-3-vinylbenzene) was completed in flow-continuous reactor. After the reaction was completed, the mixture was performed as the workup protocol and purified through chromatography on silica with hexane/dichloromethane (6/1) as the elution to afford the pure cycloadduct as clear oil.

R_f (hexane/dichloromethane 6/1): 0.30

^{1}H NMR (300 MHz, $CDCl_3$): δ 7.39 (m, 1H), 7.33 (m, 1H), 7.19 (AA' of AA'BB', J = 8.4 Hz, 2H), 7.14 (m, 2H), 6.92 (BB' of AA'BB', J = 8.7 Hz, 2H), 3.84 (s, 3H), 3.41 (q, J = 8.1 Hz, 1H), 2.96 (t, J = 9.6 Hz, 1H), 2.57 (dt, J = 2.1, 10.2 Hz, 1H), 2.38 (m, 1H), 1.73 (q, J = 9.9 Hz, 1H), 1.24 (d, J = 6.6 Hz, 3H).

^{13}C NMR (300 MHz, $CDCl_3$): δ 158.24, 147.04, 135.29, 129.83, 129.72, 129.05, 127.77, 125.40, 122.50, 113.90, 55.56, 55.28, 43.85, 35.53, 33.91, 20.42.

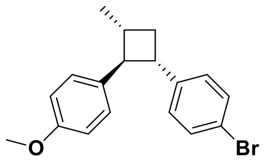

1-bromo-4-((1S,2S,3R)-2-(4-methoxyphenyl)-3-methylcyclobutyl)benzene: The synthesis (0.75 g *E*-anethole, 6 mL 1-bromo-4-vinylbenzene) was completed in flow-continuous reactor. After the reaction was completed, the mixture was performed as the workup protocol and purified through chromatography on silica with hexane/dichloromethane (6/1) as the elution to afford the pure cycloadduct as clear oil.

R_f (hexane/dichloromethane 6/1): 0.41

^{1}H NMR (300 MHz, $CDCl_3$): δ 7.41 (d, J = 8.1 Hz, 2H), 7.18 (AA' of AA’BB’, J = 8.7 Hz, 2H), 7.08 (d, J = 8.4 Hz, 2H), 6.89 (BB' of AA’BB’, J = 8.7 Hz, 2H), 3.83 (s, 3H), 3.35 (q, J = 8.1 Hz, 1H), 2.93 (t, J = 9.6 Hz, 1H), 2.53 (q, J = 10.2 Hz, 1H), 2.38 (m, 1H), 1.68 (q, J = 9.9 Hz, 1H), 1.22 (d, J = 6.3 Hz, 3H).

^{13}C NMR (300 MHz, $CDCl_3$): δ 158.19, 143.60, 135.37, 131.29, 128.44, 127.79, 119.64, 113.85, 55.74, 55.29, 43.76, 35.48, 33.81, 20.49.

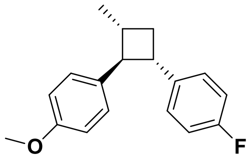

1-fluoro-4-((1S,2S,3R)-2-(4-methoxyphenyl)-3-methylcyclobutyl)benzene: The synthesis (0.75 g *E*-anethole, 6 mL 1-fluoro-4-vinylbenzene) was completed in flow-continuous reactor. After the reaction was completed, the mixture was performed as the workup protocol and purified through chromatography on silica with hexane/dichloromethane (8/1) as the elution to afford the pure cycloadduct as clear oil.

R_f (hexane/dichloromethane 8/1): 0.36

^{1}H NMR (300 MHz, $CDCl_3$): δ 7.05 (m, 4H), 6.85 (t, J = 8.7 Hz, 3H), 6.77 (BB’ of AA’BB’, J = 8.7 Hz, 2H), 3.69 (s, 3H), 3.26 (q, J = 8.4 Hz, 1H), 2.78 (t, J = 9.6 Hz, 1H), 2.42 (q, J = 10.2 Hz, 1H), 2.23 (m, 1H), 1.57 (q, J = 9.9 Hz, 1H), 1.10 (d, J = 6.6 Hz, 3H).

^{13}C NMR (300 MHz, $CDCl_3$): δ 162.93 ($^{1}J_{CF}$ = 966 Hz), 158.20, 140.34 ($^{1}J_{CF}$ = 12 Hz), 135.54, 128.04 ($^{3}J_{CF}$ = 33 Hz), 127.77, 115.09 ($^{2}J_{CF}$ = 84 Hz), 113.86, 55.98, 55.26, 43.58, 35.45, 34.12, 20.45.

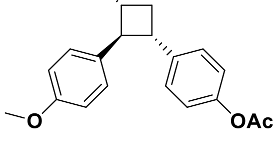

4-((1S,2S,3R)-2-(4-methoxyphenyl)-3-methylcyclobutyl)phenyl acetate: The synthesis (0.75 g *E*-anethole, 7.5 mL 4-vinylphenyl acetate) was completed in flow-continuous reactor. After the reaction was completed, the mixture was performed as the workup protocol and purified through chromatography on silica with hexane/diethyl ether (5/1) as the elution to afford the pure cycloadduct as clear oil.

R_f (hexane/diethyl ether 5/1): 0.42

^{1}H NMR (300 MHz, $CDCl_3$): δ 7.17 (AA' of AA'BB', J = 4.8 Hz, 2H), 7.14 (AA' of AA'BB', J = 4.8 Hz, 2H), 6.96 (BB' of AA'BB', J = 8.7 Hz, 2H), 6.84 (BB' of AA'BB', J = 8.7 Hz, 2H), 3.75 (s, 3H), 3.34 (td, J = 1.2, 8.1 Hz, 1H), 2.89 (t, J = 9.6 Hz, 1H), 2.50 (q, J = 9.9 Hz, 1H), 2.29 (m, 1H), 2.24 (s, 3H), 1.66 (q, J = 10.2 Hz, 1H), 1.14 (d, J = 6.3 Hz, 3H).

^{13}C NMR (300 MHz, $CDCl_3$): δ 169.71, 158.13, 148.77, 142.26, 135.58, 127.80, 127.56, 121.23, 113.81, 55.66, 55.25, 43.55, 35.58, 33.98, 21.14, 20.47.

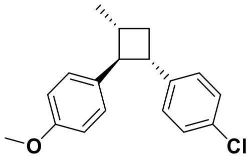

1-chloro-4-((1S,2S,3R)-2-(4-methoxyphenyl)-3-methylcyclobutyl)benzene: The synthesis (0.75 g *E*-anethole, 6 mL 1-chloro-4-vinylbenzene) was completed in flow-continuous reactor. After the reaction was completed, the mixture was performed as the workup protocol and purified through chromatography on silica with hexane/dichloromethane (6/1) as the elution to afford the pure cycloadduct as clear oil.

R_f (hexane/dichloromethane 6/1): 0.27

^{1}H NMR (300 MHz, $CDCl_3$): δ 7.26 (d, J = 8.4 Hz, 2H), 7.18 (AA' of AA'BB', J = 8.4 Hz, 2H), 7.14 (d, J = 8.4 Hz, 2H), 6.92 (BB' of AA'BB', J = 8.7 Hz, 2H), 3.84 (s, 3H), 3.40 (q, J = 8.1 Hz, 1H), 2.93 (t, J = 9.6 Hz, 1H), 2.57 (q, J = 10.2 Hz, 1H), 2.39 (m, 1H), 1.72 (q, J = 9.9 Hz, 1H), 1.24 (d, J = 6.6 Hz, 3H).

^{13}C NMR (300 MHz, $CDCl_3$): δ 158.18, 143.09, 135.40, 131.58, 128.34, 128.02, 127.79, 113.85, 55.80, 55.28, 43.70, 35.48, 33.88, 20.48.

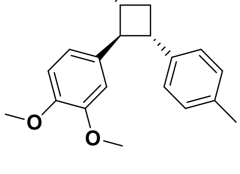

1,2-dimethoxy-4-((1S,2R,4S)-2-methyl-4-(p-tolyl)cyclobutyl)benzene: The synthesis (0.8 mL (*E*)-1,2-dimethoxy-4-(prop-1-en-1-yl)benzene, 6 mL 4-methylstyrene) was completed in flow-continuous reactor. After the reaction was

completed, the mixture was performed as the workup protocol and purified by chromatography on silica with hexane/ethyl acetate (8/1) as the elution to afford the pure cycloadduct as clear oil.

R_f (hexane/ethyl acetate 8/1): 0.40

^{1}H NMR (300 MHz, $CDCl_3$): δ 7.12 (m, 4H), 6.84 (m, 2H), 6.69 (m, 1H), 3.89 (s, 6H), 3.40 (q, J = 9.9 Hz, 1H), 2.94 (t, J = 9.6 Hz, 1H), 2.51 (dt, J = 2.1, 10.2 Hz, 1H), 2.41 (m, 1H), 2.34 (s, 3H), 1.70 (q, J = 9.9 Hz, 1H), 1.22 (d, J = 6.3 Hz, 3H).

^{13}C NMR (300 MHz, $CDCl_3$): δ 148.88, 147.50, 141.60, 136.57, 135.44, 128.94, 126.54, 118.68, 111.25, 110.26, 56.17, 55.95, 55.85, 43.84, 35.41, 33.89, 21.03, 20.57.

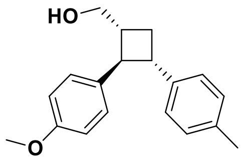

((1R,2R,3S)-2-(4-methoxyphenyl)-3-(p-tolyl)cyclobutyl)methanol: The synthesis (0.8 g, (*E*)-3-(4-methoxyphenyl)prop-2-en-1-ol, 6 mL 4-methylstyrene) was completed in flow-continuous reactor. After the reaction was completed, the mixture was performed as the workup protocol and purified through chromatography on silica with hexane/ethyl acetate (8/1) as the elution to afford the pure cycloadduct as clear oil.

R_f (hexane/ethyl acetate 8/1): 0.12

^{1}H NMR (300 MHz, $CDCl_3$): δ 7.12 (AA', J = 8.4 Hz, 2H), 7.02 (m, 4H), 6.77 (BB', J = 8.7 Hz, 2H), 3.70 (s, 3H), 3.68 (m, 1H), 3.64 (m, 1H), 3.33 (td, J = 1.8, 8.1 Hz, 1H), 3.13 (t, J = 9.3 Hz, 1H), 2.45 (m, 1H), 2.34 (m, 1H), 2.23 (s, 3H), 1.82 (q, J = 9.9 Hz, 1H), 1.47 (br s, 1H).

^{13}C NMR (300 MHz, $CDCl_3$): δ 158.17, 141.24, 135.64, 135.49, 129.00, 127.91, 126.55, 113.86, 66.05, 55.27, 50.22, 43.96, 41.62, 28.63, 21.03.

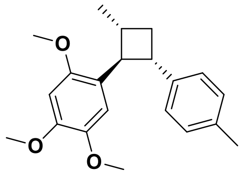

1,2,4-trimethoxy-5-((1S,2R,4S)-2-methyl-4-(p-tolyl)cyclobutyl)benzene: The synthesis (1 g (*E*)-1,2,4-trimethoxy-5-(prop-1-en-1-yl)benzene, 6 mL 4-methylstyrene) was completed in flow-continuous reactor. After the reaction was

completed, the mixture was performed as the workup protocol and purified through chromatography on silica with hexane/ethyl acetate (2/1) as the elution to afford the pure cycloadduct as clear oil.

R_f (hexane/ethyl acetate 2/1): 0.77

^{1}H NMR (300 MHz, $CDCl_3$): δ 7.11 (m, 4H), 6.92 (s, 1H), 6.51 (s, 1H), 3.89 (s, 3H), 3.88 (s, 3H), 3.68 (s, 3H), 3.45 (m, 1H), 2.39 (m, 1H), 2.54 (m, 1H), 2.38 (m, 1H), 2.32 (s, 3H), 1.68 (q, J = 9.6 Hz, 1H), 1.20 (d, J = 6.6 Hz, 3H).

^{13}C NMR (300 MHz, $CDCl_3$): δ 151.87, 147.80, 143.21, 141.97, 135.13, 128.73, 126.49, 123.84, 112.09, 98.30, 56.85, 56.66, 56.20, 49.34, 42.80, 35.39, 34.34, 20.98, 20.69.

^{1}H and ^{13}C NMR spectra

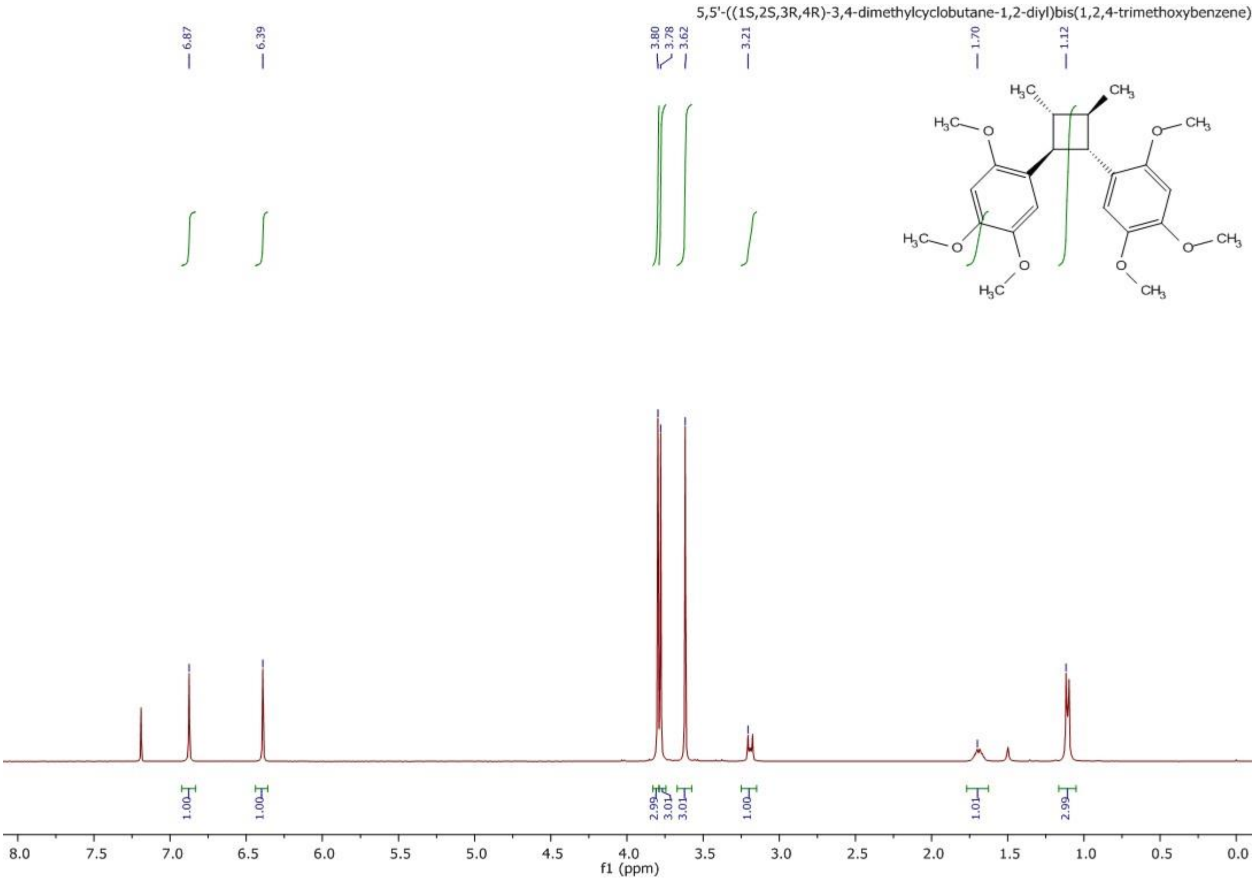

5,5'-((1S,2S,3R,4R)-3,4-dimethylcyclobutane-1,2-diyl)bis(1,2,4-trimethoxybenzene)

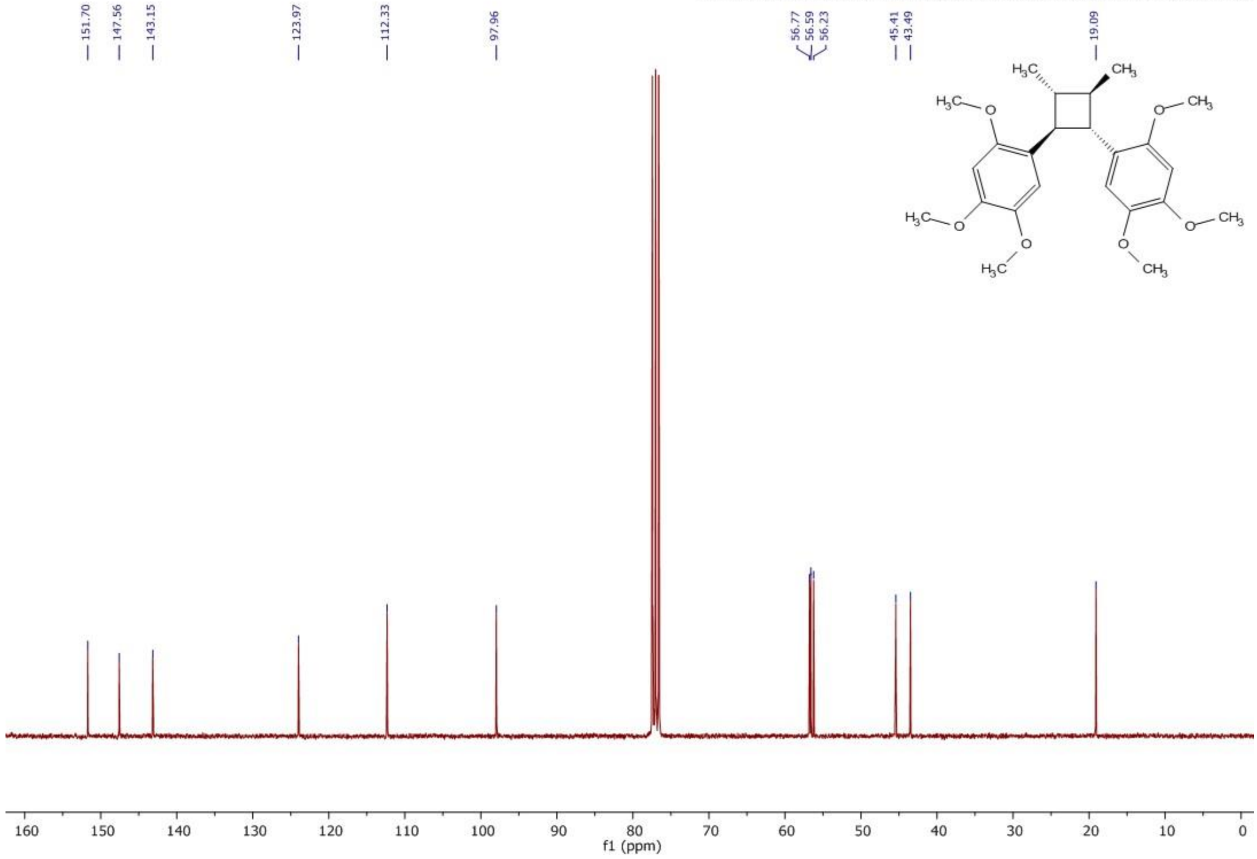

1-methoxy-4-((1S,2R,4S)-2-methyl-4-phenylcyclobutyl)benzene

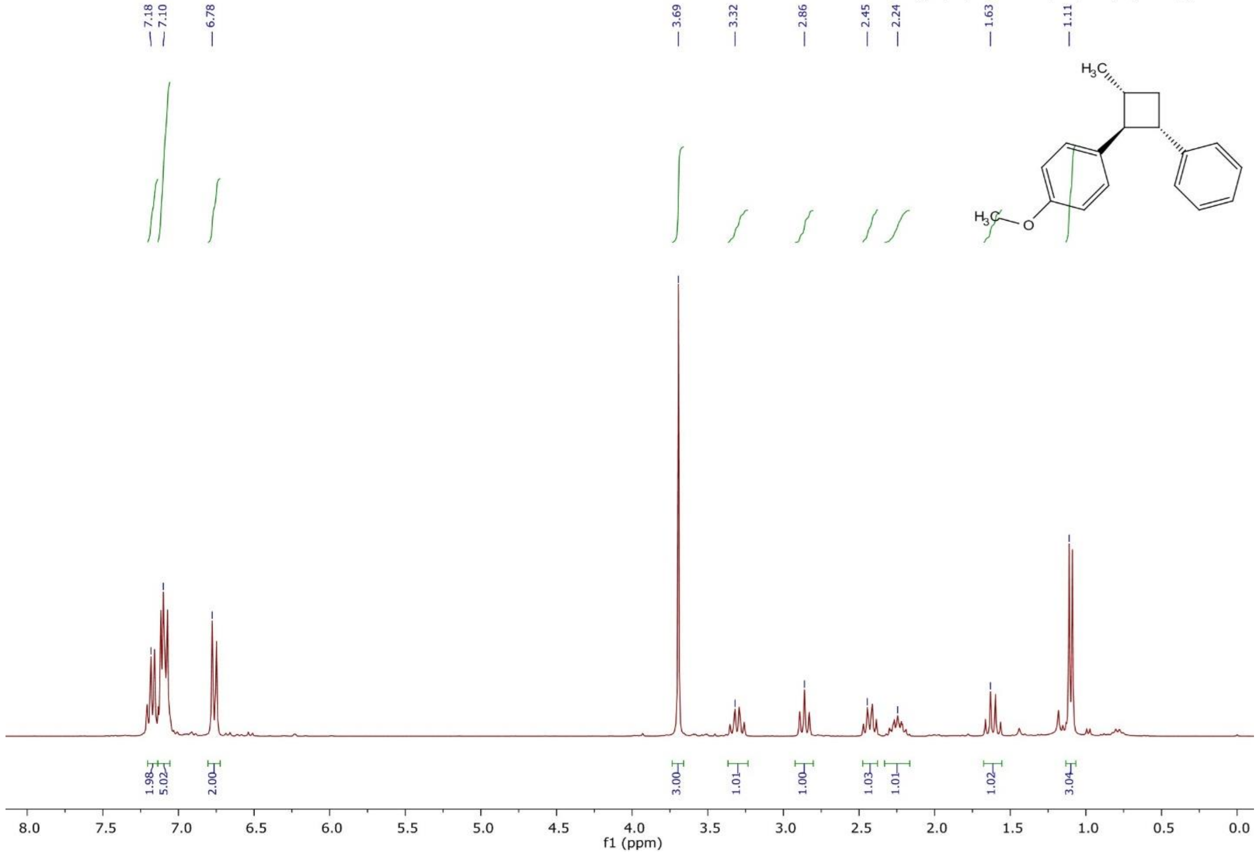

1-methoxy-4-((1S,2R,4S)-2-methyl-4-phenylcyclobutyl)benzene

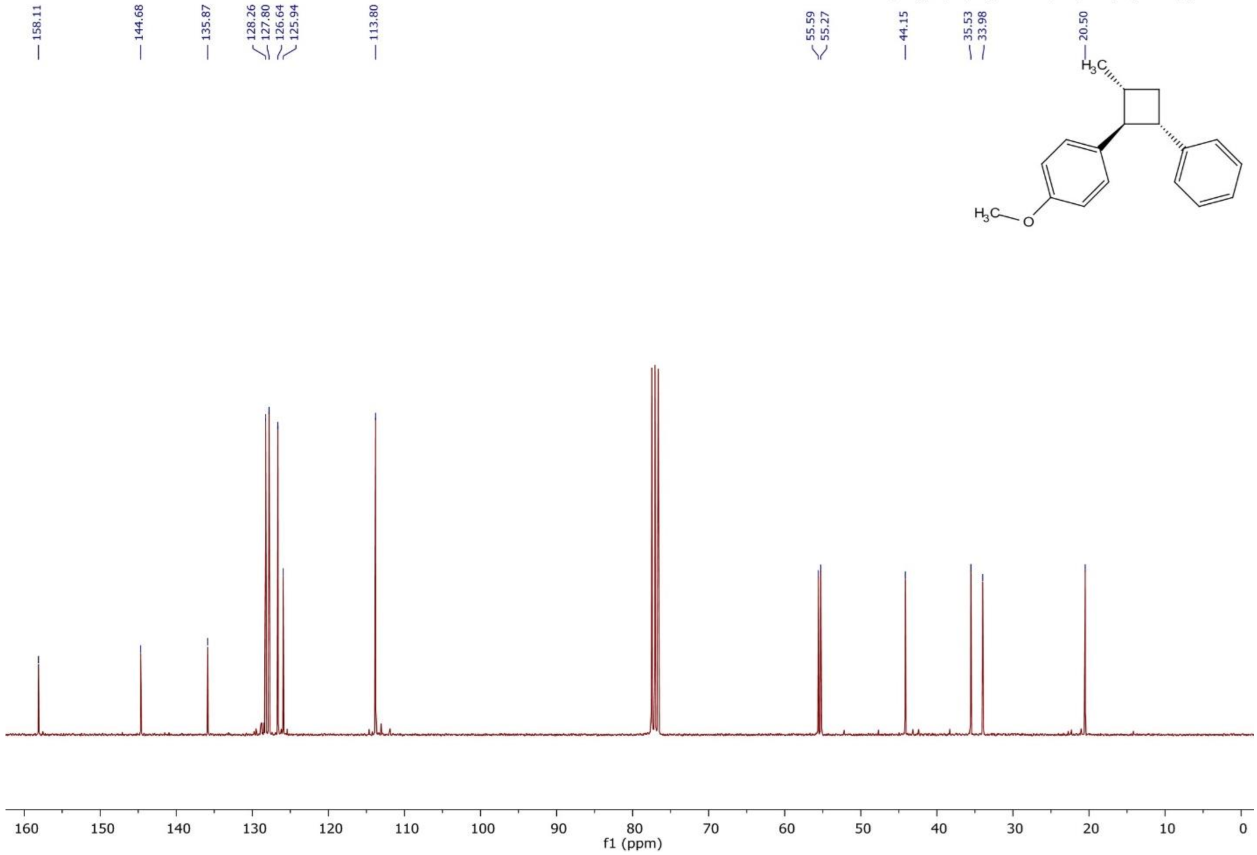

1-((1S,2S,3R)-2-(4-methoxyphenyl)-3-methylcyclobutyl)-2-methylbenzene

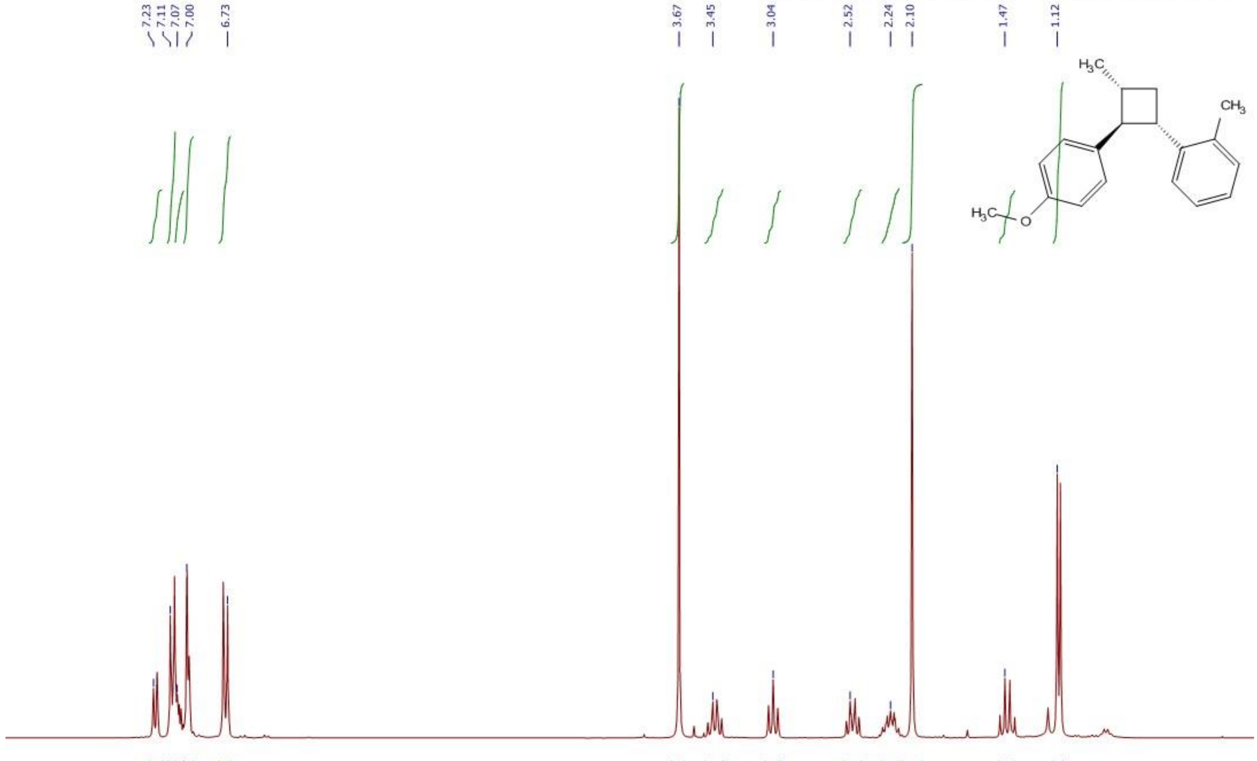

1-((1S,2S,3R)-2-(4-methoxyphenyl)-3-methylcyclobutyl)-2-methylbenzene

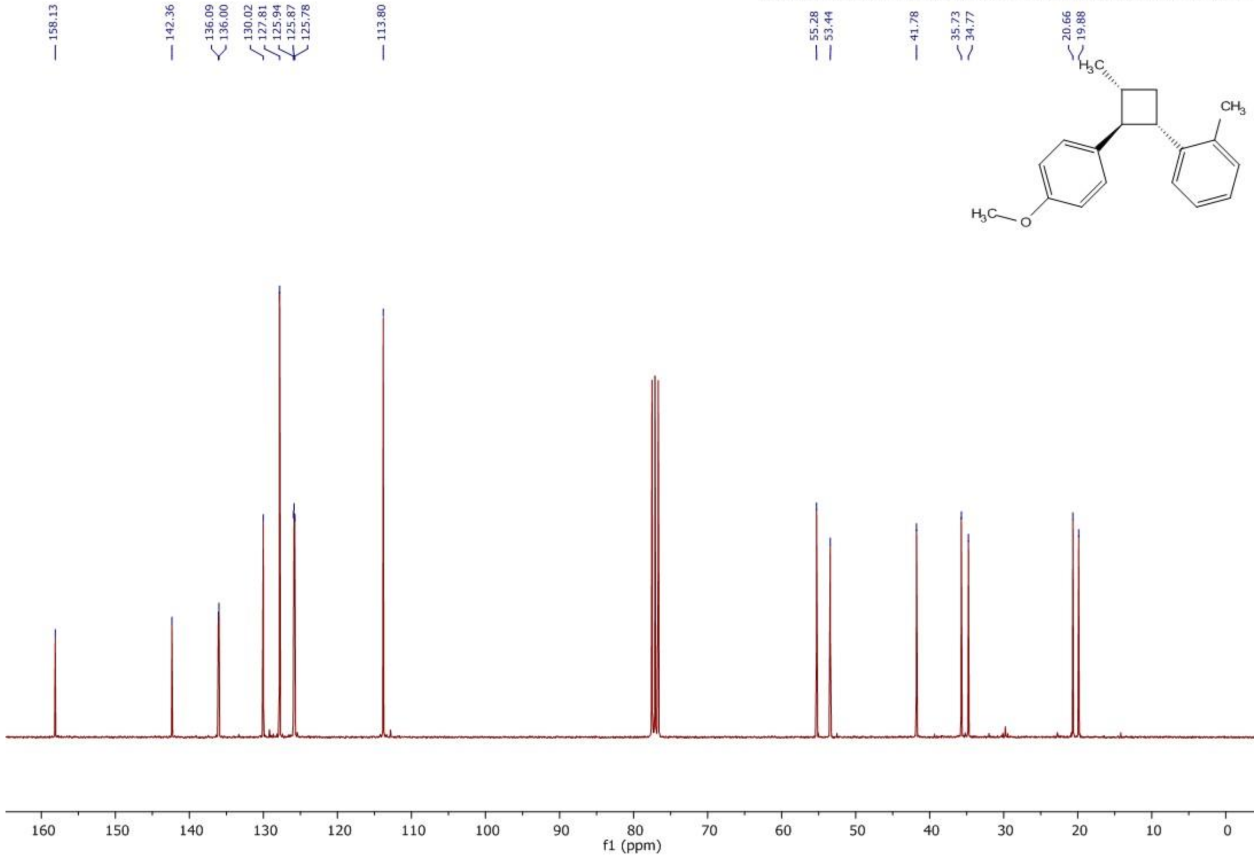

1-((1S,2S,3R)-2-(4-methoxyphenyl)-3-methylcyclobutyl)-3-methylbenzene

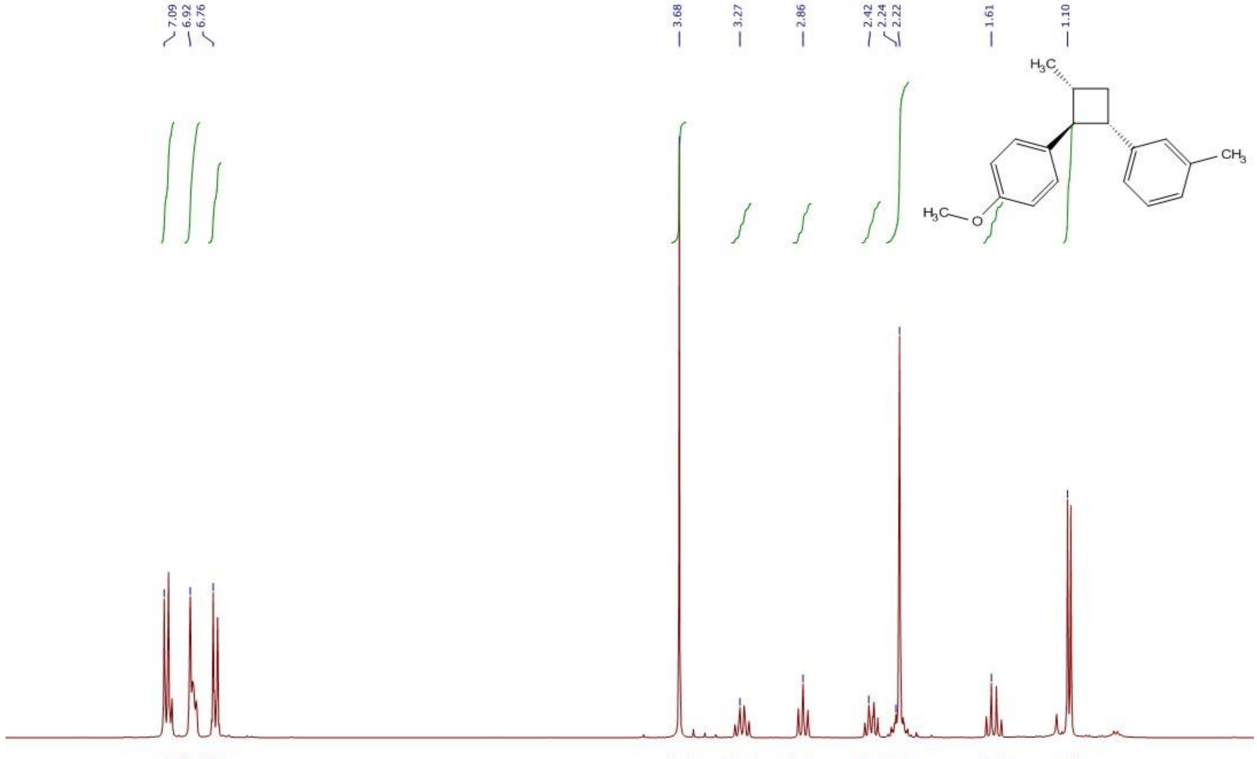

1-((1S,2S,3R)-2-(4-methoxyphenyl)-3-methylcyclobutyl)-3-methylbenzene

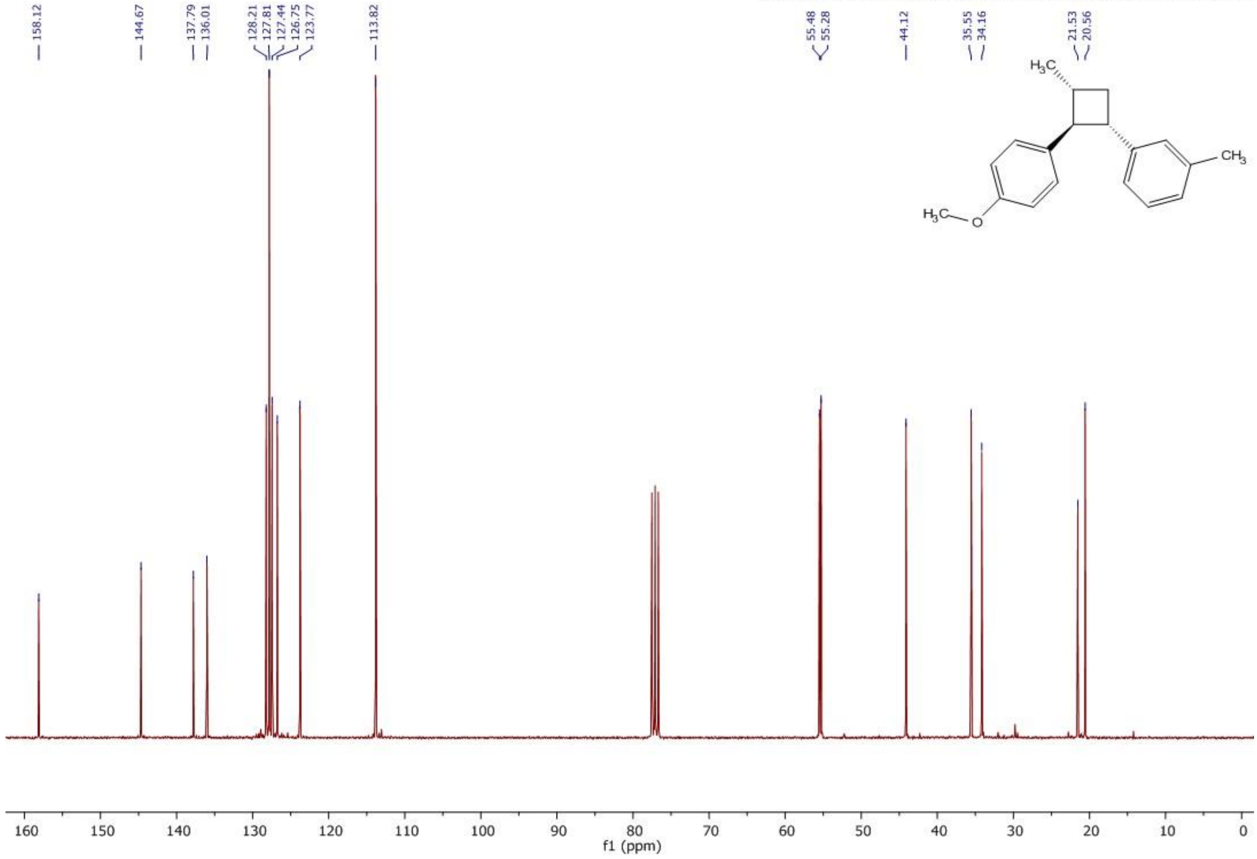

1-methoxy-4-((1S,2R,4S)-2-methyl-4-(p-tolyl)cyclobutyl)benzene

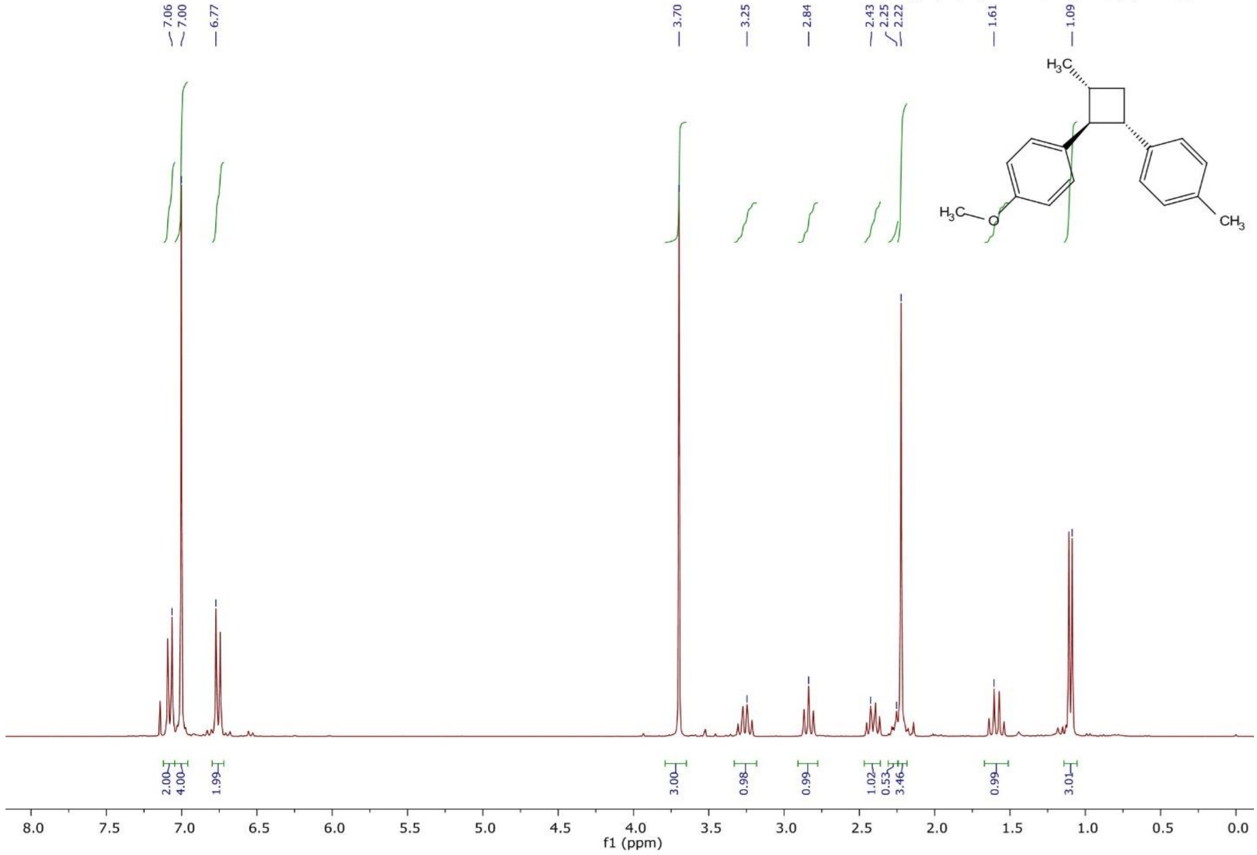

1-methoxy-4-((1S,2R,4S)-2-methyl-4-(p-tolyl)cyclobutyl)benzene

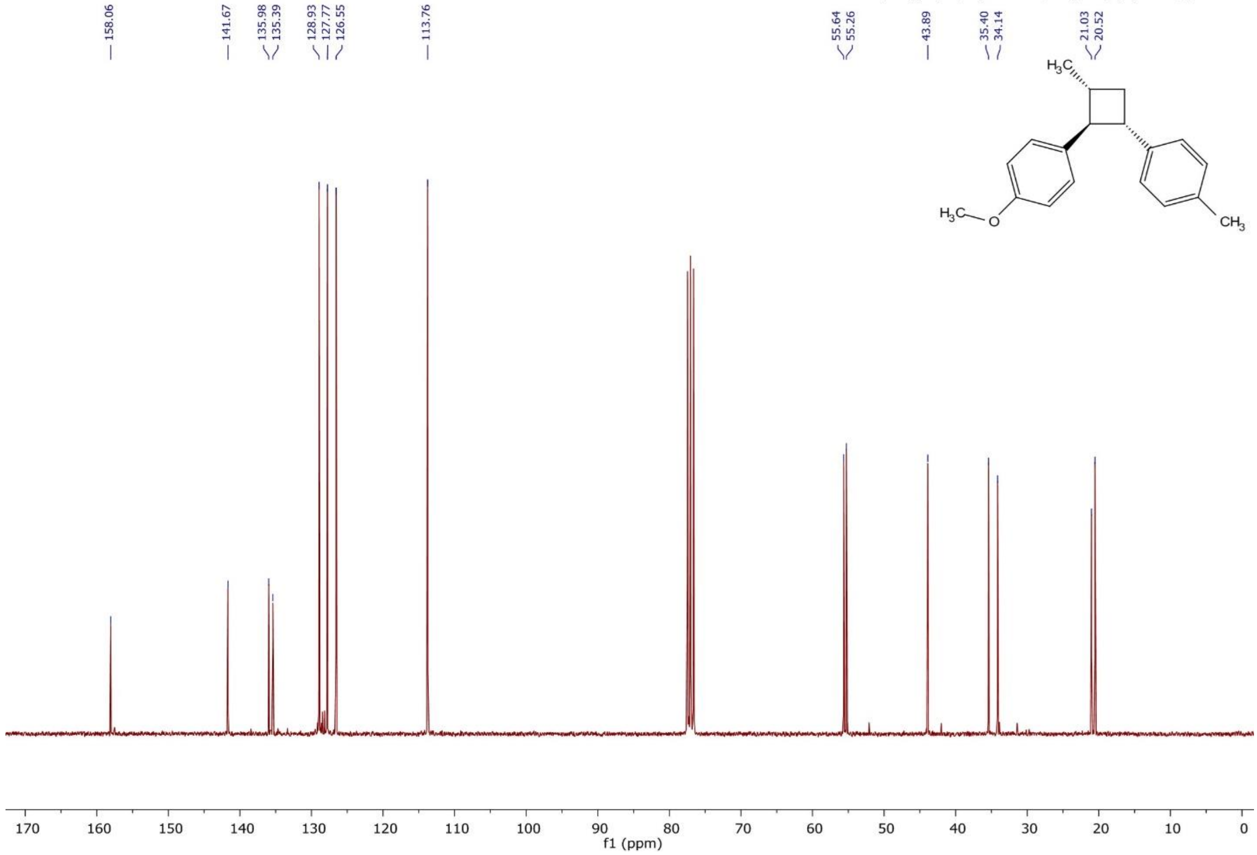

1-bromo-2-((1S,2S,3R)-2-(4-methoxyphenyl)-3-methylcyclobutyl)benzene

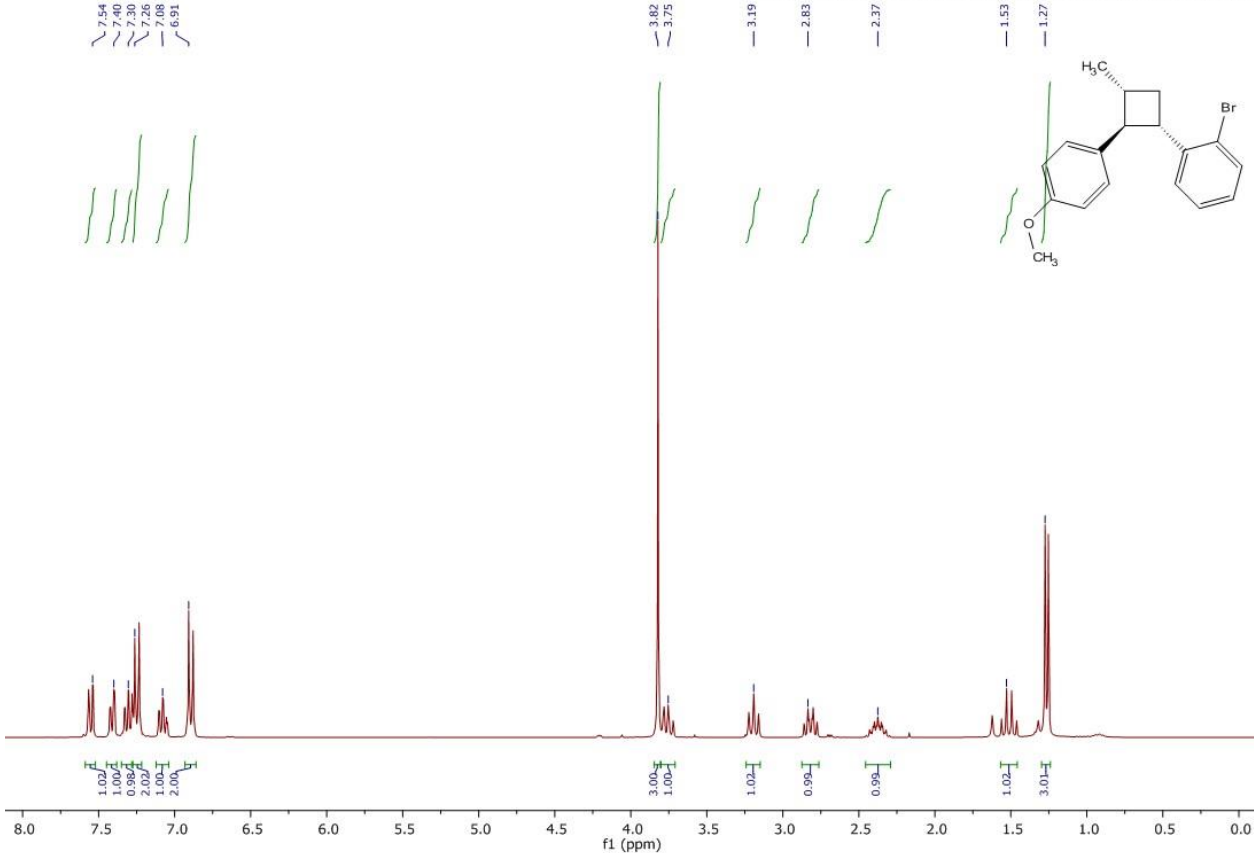

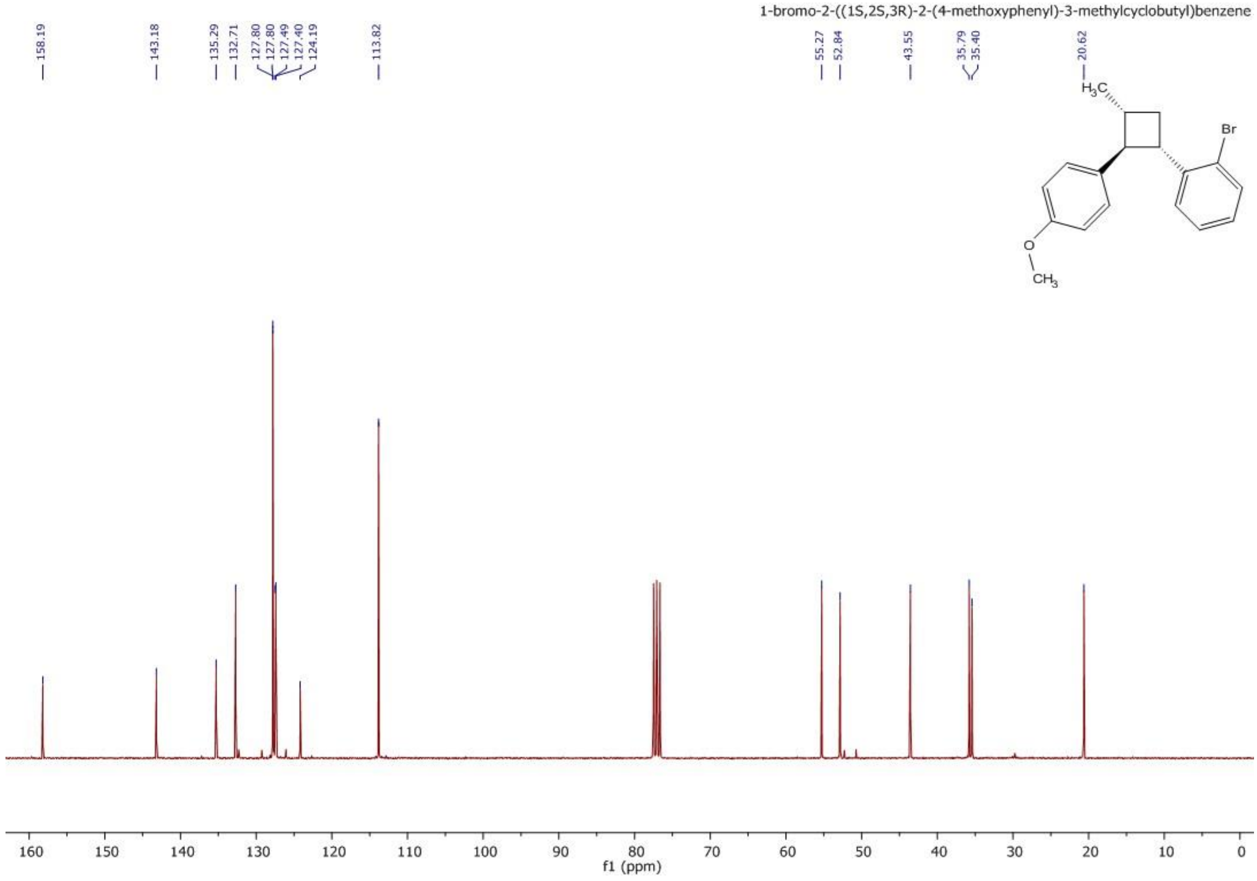
1-bromo-2-((1S,2S,3R)-2-(4-methoxyphenyl)-3-methylcyclobutyl)benzene
158.19
143.18
135.29
132.71
127.80
127.80
127.49
127.40
124.19
113.82
55.27
52.84
43.55
35.79
35.40
20.62
H3C
Br
O
CH3
160 150 140 130 120 110 100 90 80 70 60 50 40 30 20 10 0
f1 (ppm)

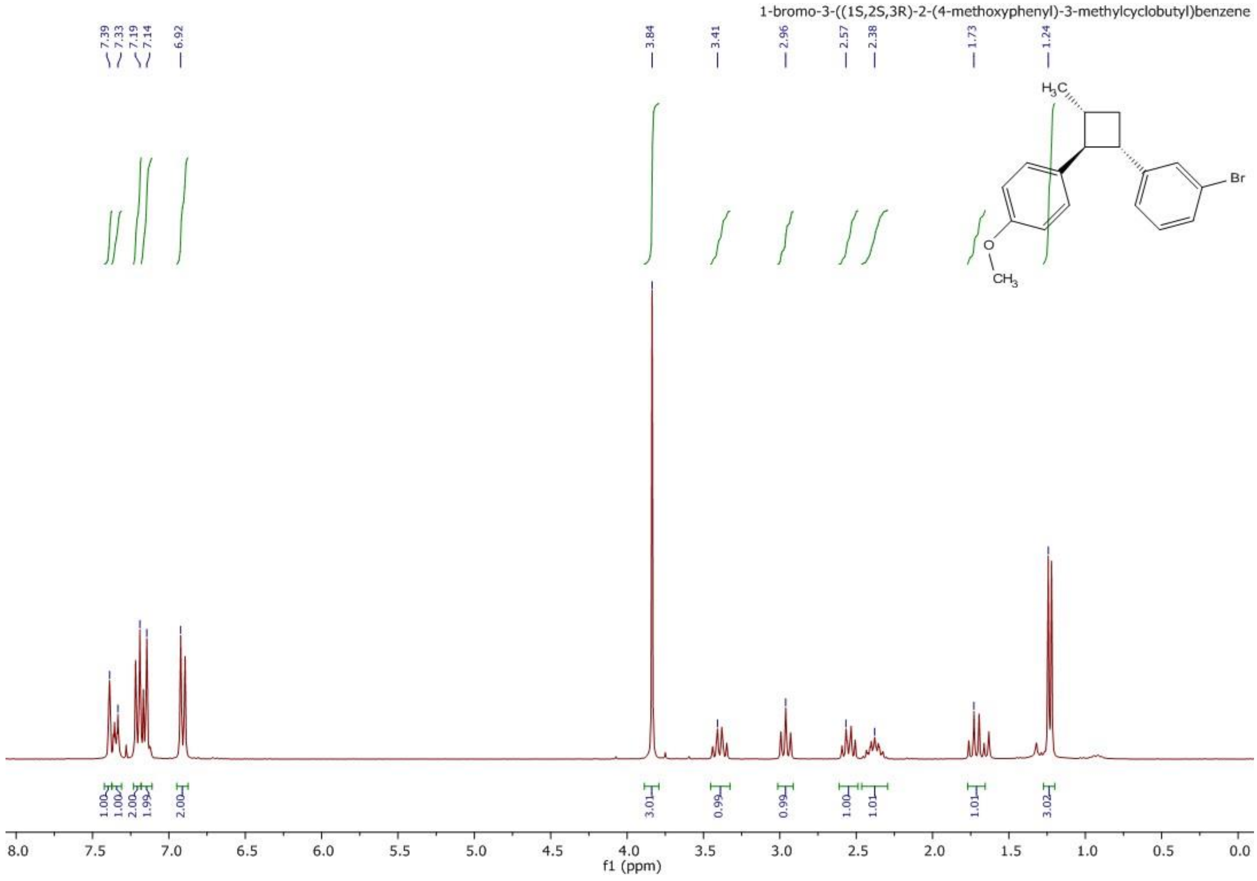
1-bromo-3-((1S,2S,3R)-2-(4-methoxyphenyl)-3-methylcyclobutyl)benzene
7.39
7.33
7.19
7.14
6.92
3.84
3.41
2.96
2.57
2.38
1.73
1.24
H3C
Br
O
CH3
1.00
1.00
2.00
1.99
2.00
3.01
0.99
0.99
1.00
1.01
1.01
3.02
8.0 7.5 7.0 6.5 6.0 5.5 5.0 4.5 4.0 3.5 3.0 2.5 2.0 1.5 1.0 0.5 0.0
f1 (ppm)

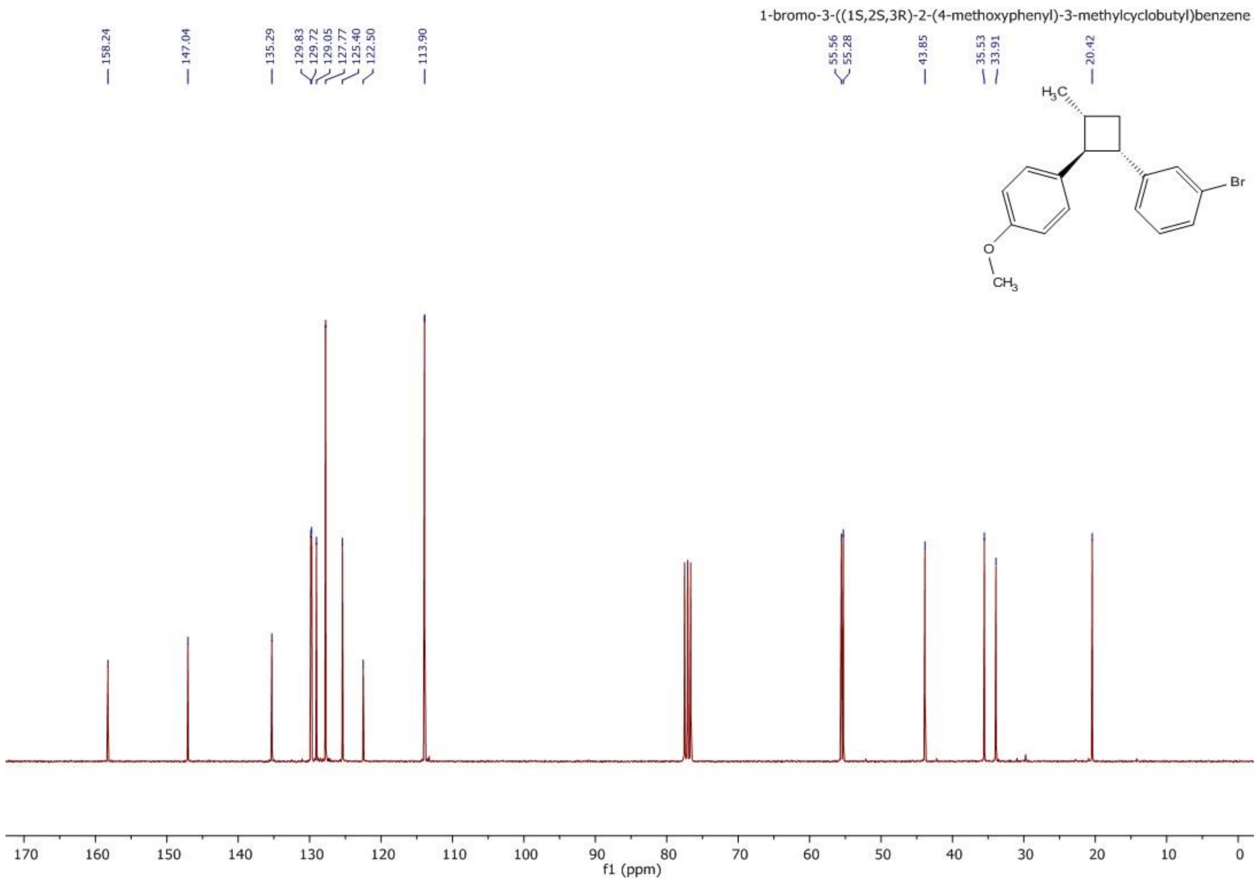
1-bromo-3-((1S,2S,3R)-2-(4-methoxyphenyl)-3-methylcyclobutyl)benzene
158.24
147.04
135.29
129.83
129.72
129.05
127.77
125.40
122.50
113.90
55.56
55.28
43.85
35.53
33.91
20.42
H3C
Br
O
CH3
f1 (ppm)

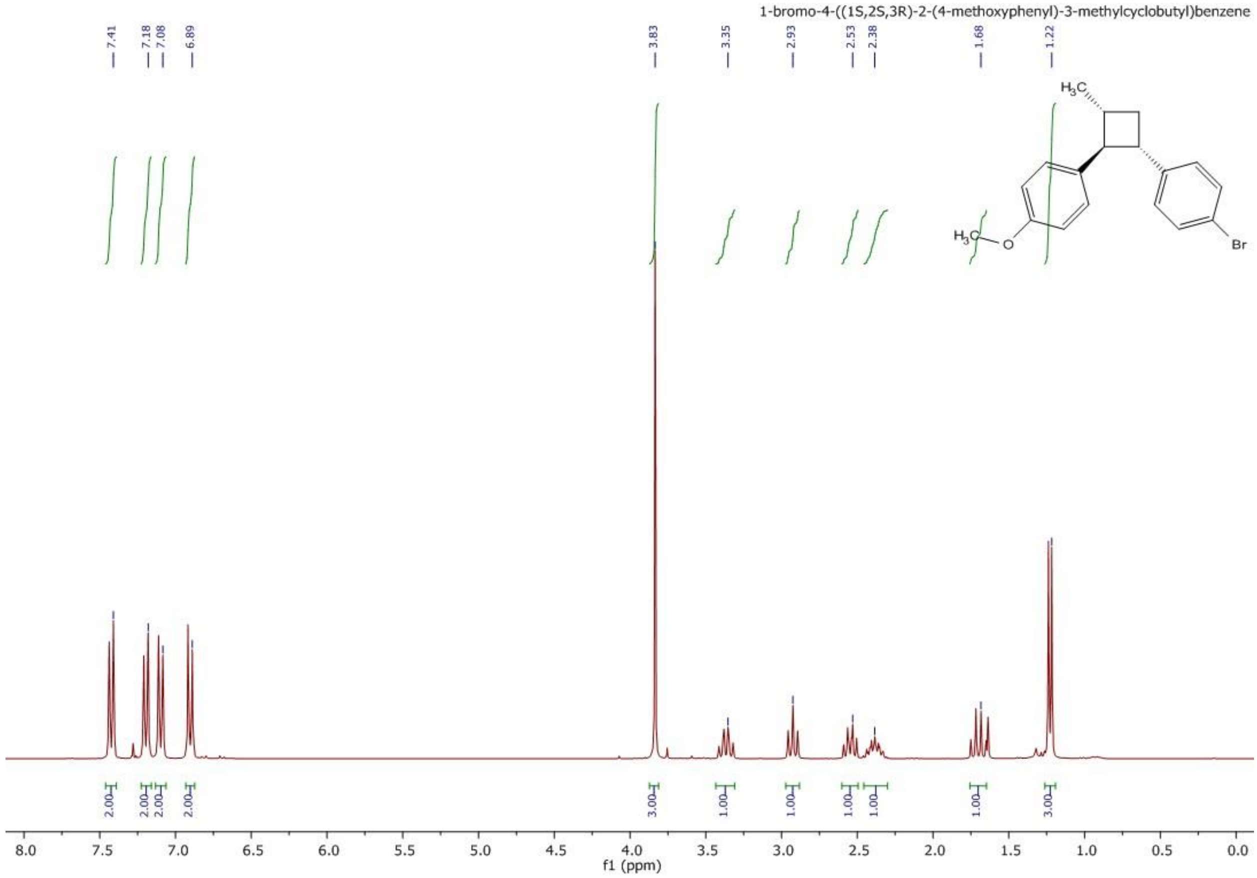
1-bromo-4-((1S,2S,3R)-2-(4-methoxyphenyl)-3-methylcyclobutyl)benzene
7.41
7.18
7.08
6.89
3.83
3.35
2.93
2.53
2.38
1.68
1.22
H3C
H3C
O
Br
f1 (ppm)

1-bromo-4-((1S,2S,3R)-2-(4-methoxyphenyl)-3-methylcyclobutyl)benzene

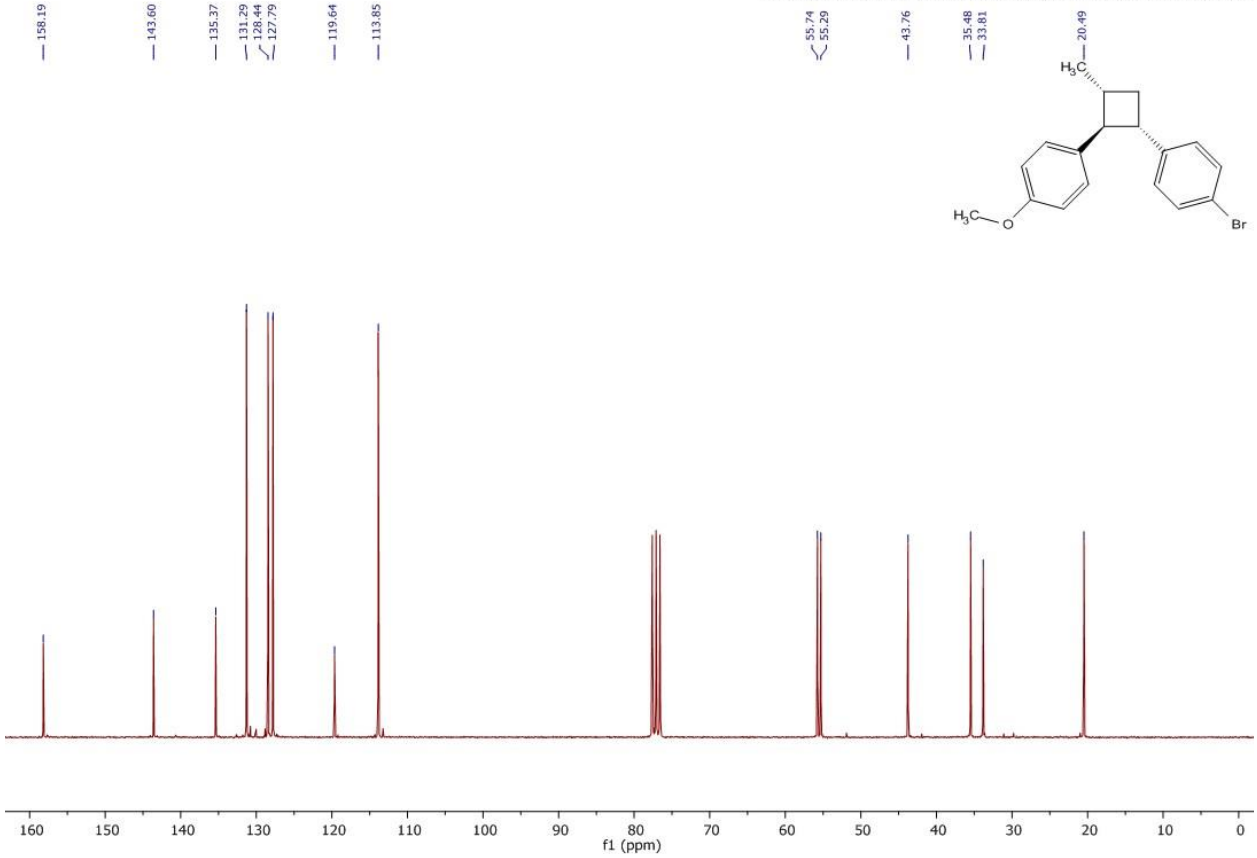

1-fluoro-4-((1S,2S,3R)-2-(4-methoxyphenyl)-3-methylcyclobutyl)benzene

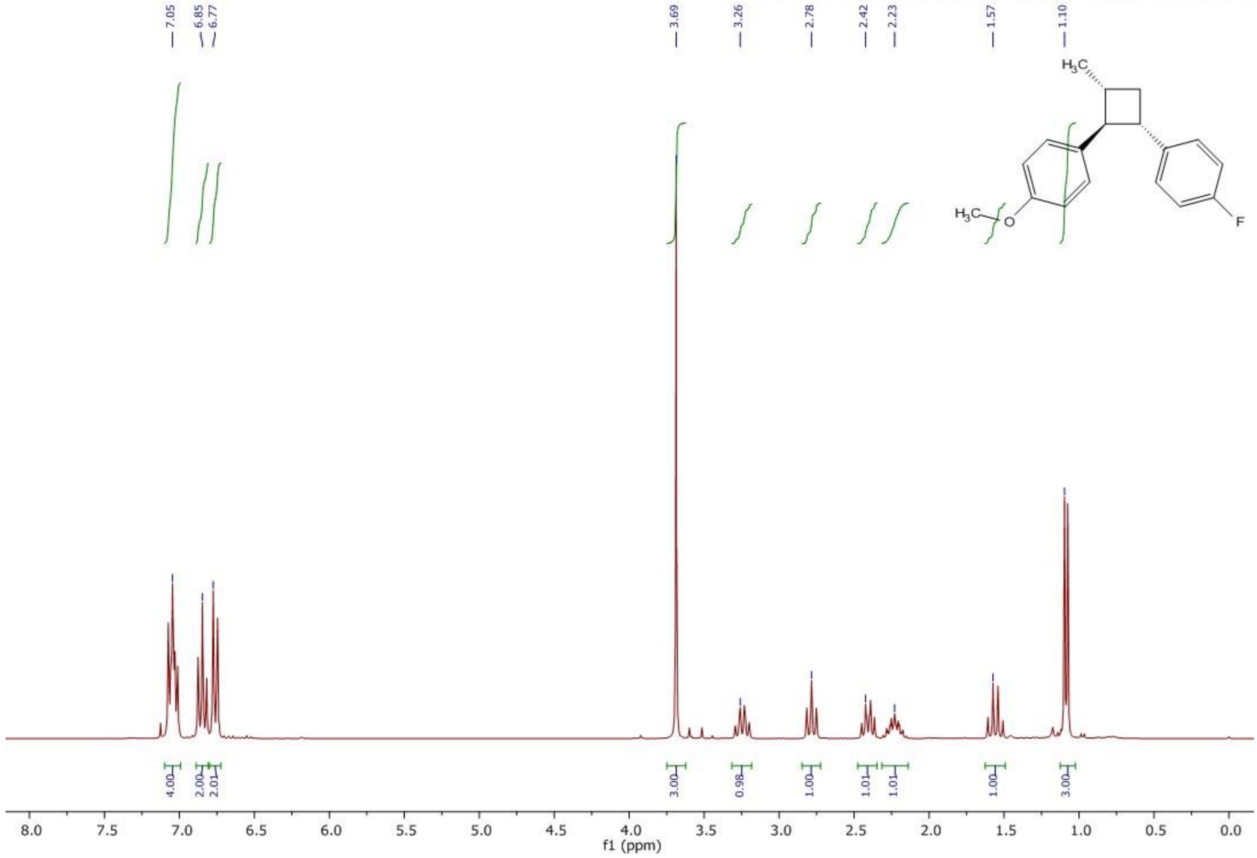

1-fluoro-4-((1S,2S,3R)-2-(4-methoxyphenyl)-3-methylcyclobutyl)benzene

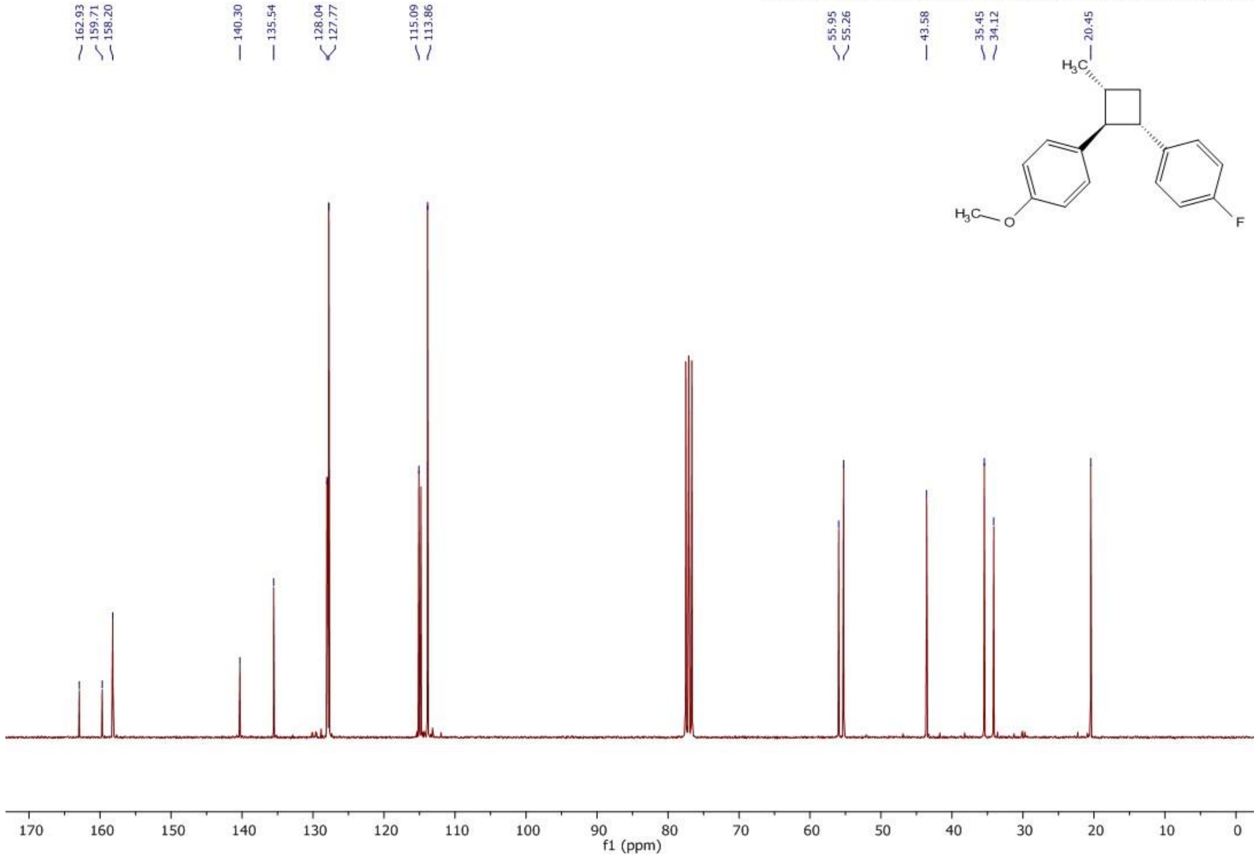

4-((1S,2S,3R)-2-(4-methoxyphenyl)-3-methylcyclobutyl)phenyl acetate

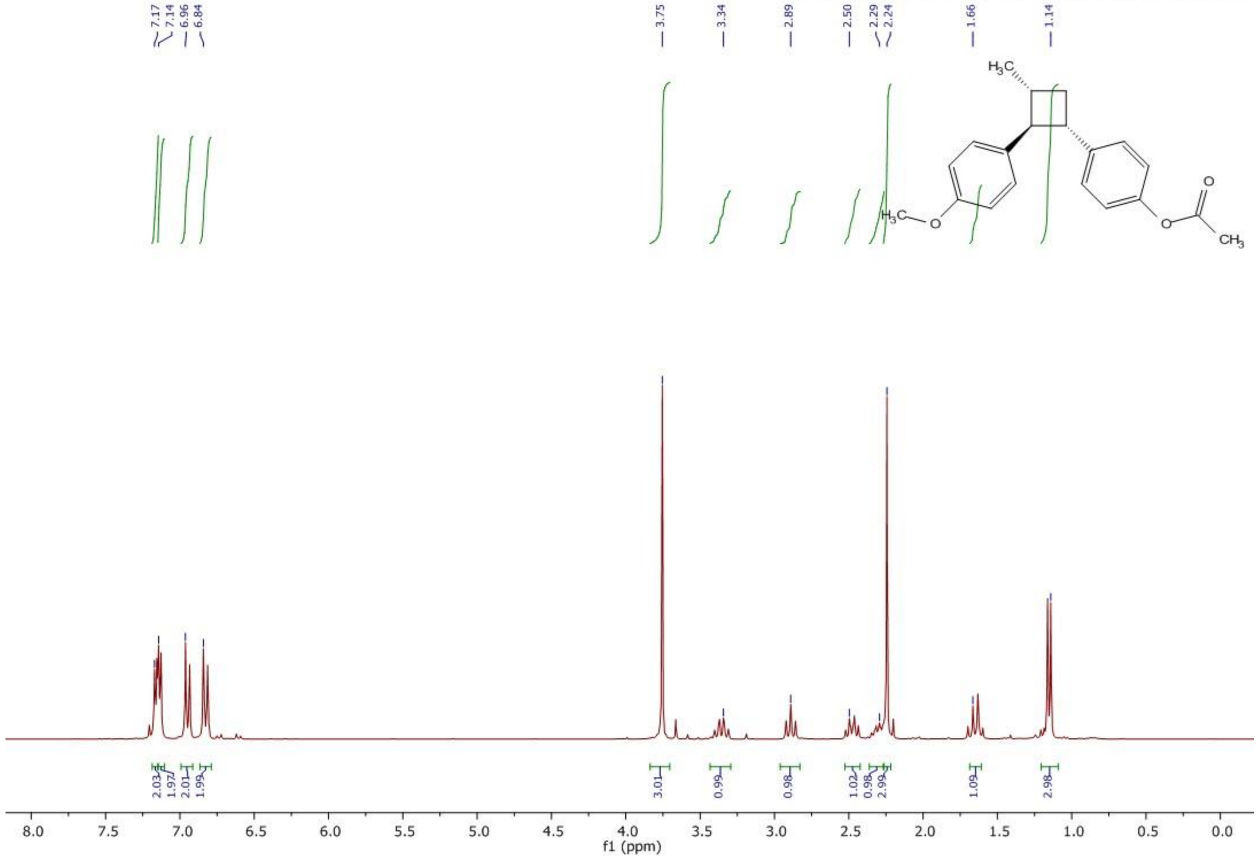

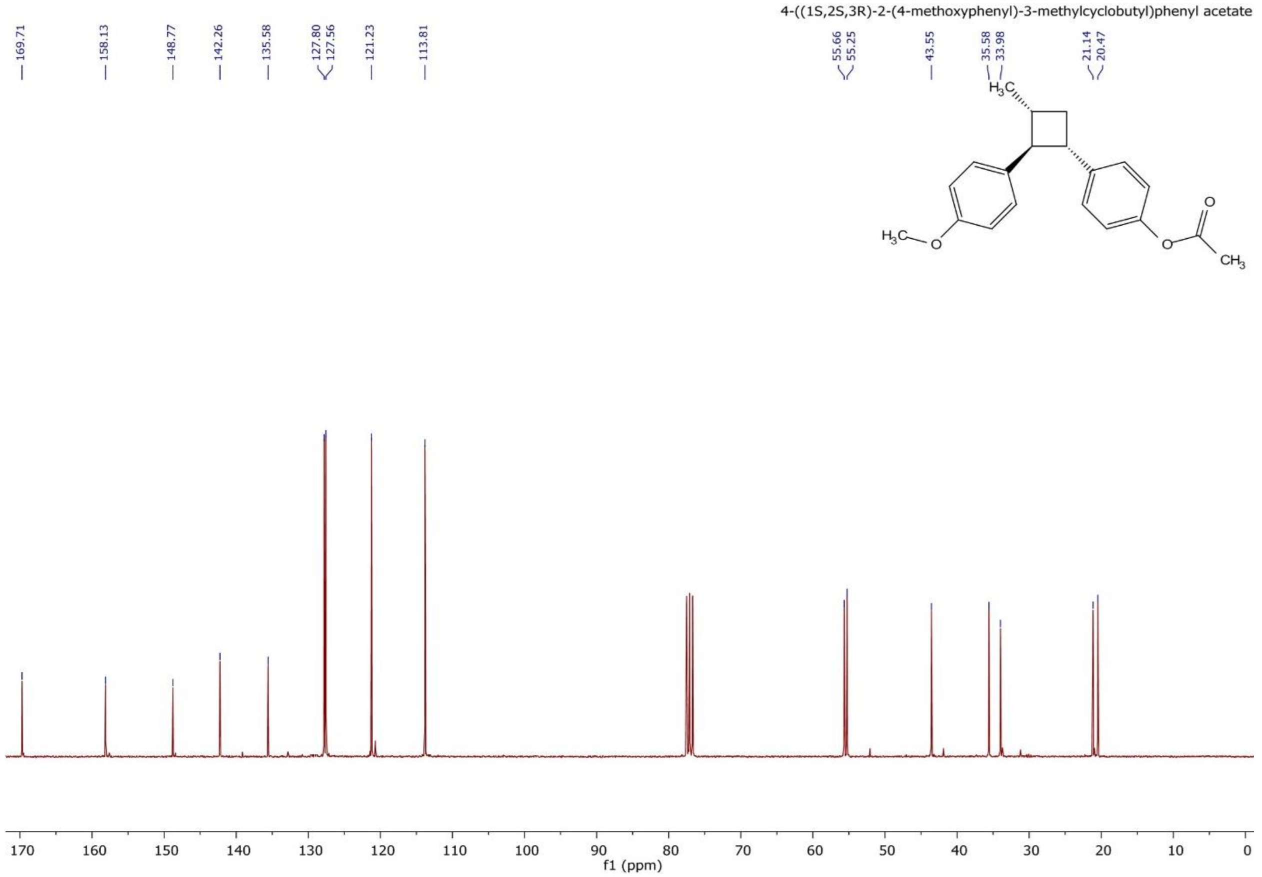
4-((1S,2S,3R)-2-(4-methoxyphenyl)-3-methylcyclobutyl)phenyl acetate
169.71
158.13
148.77
142.26
135.58
127.80
127.56
121.23
113.81
55.66
55.25
43.55
35.58
33.98
21.14
20.47
f1 (ppm)

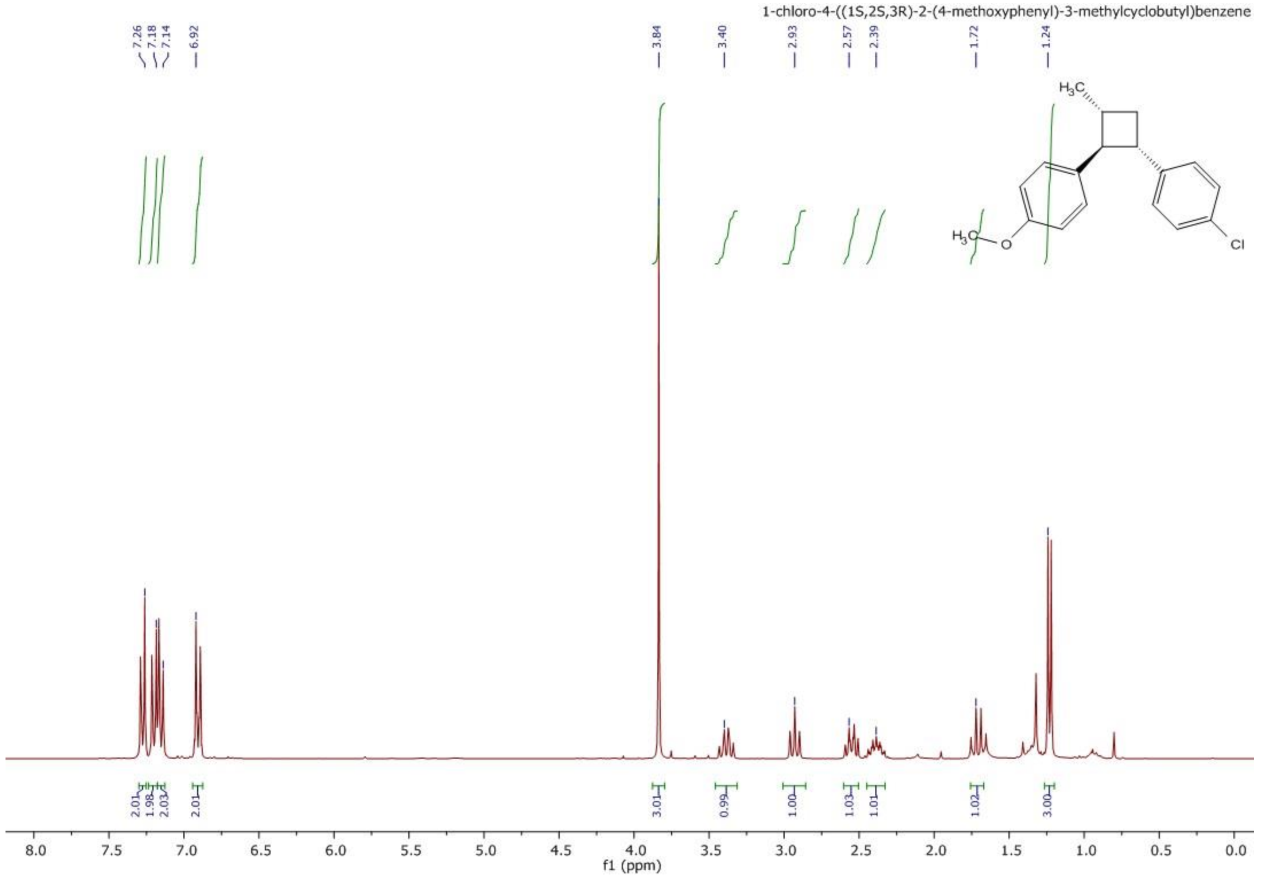
1-chloro-4-((1S,2S,3R)-2-(4-methoxyphenyl)-3-methylcyclobutyl)benzene
7.26
7.18
7.14
6.92
3.84
3.40
2.93
2.57
2.39
1.72
1.24
2.01
1.98
2.03
2.01
3.01
0.99
1.00
1.03
1.01
1.02
3.00
f1 (ppm)

1-chloro-4-((1S,2S,3R)-2-(4-methoxyphenyl)-3-methylcyclobutyl)benzene

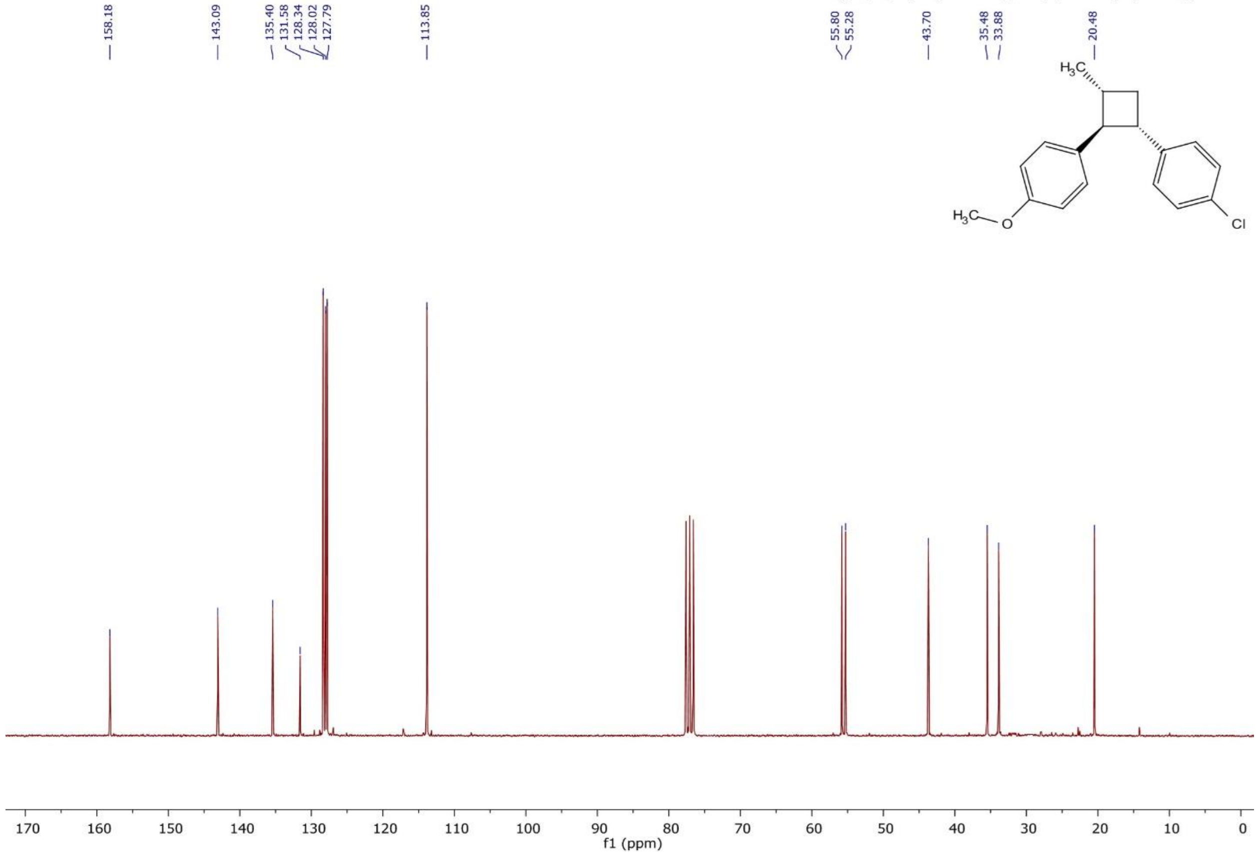

1,2-dimethoxy-4-((1S,2R,4S)-2-methyl-4-(p-tolyl)cyclobutyl)benzene

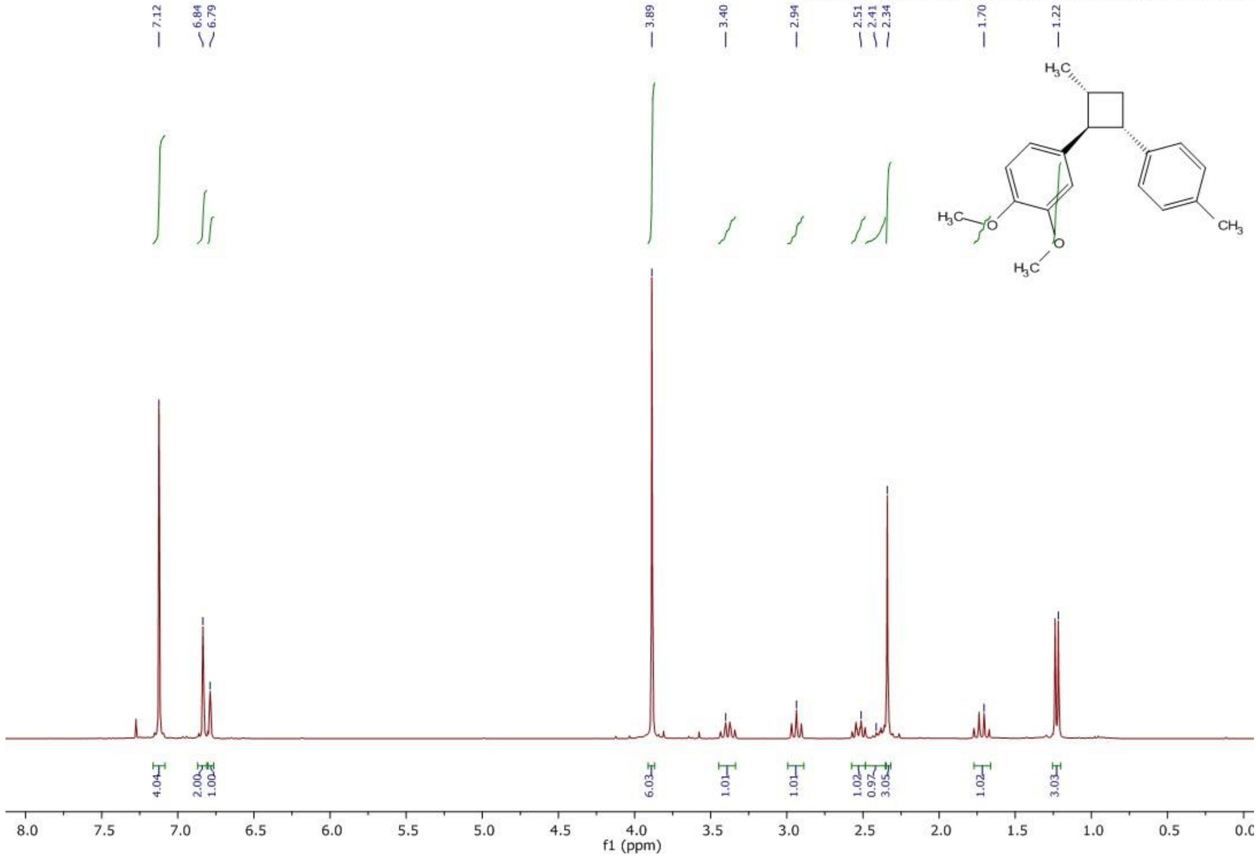

1,2-dimethoxy-4-((1S,2R,4S)-2-methyl-4-(p-tolyl)cyclobutyl)benzene

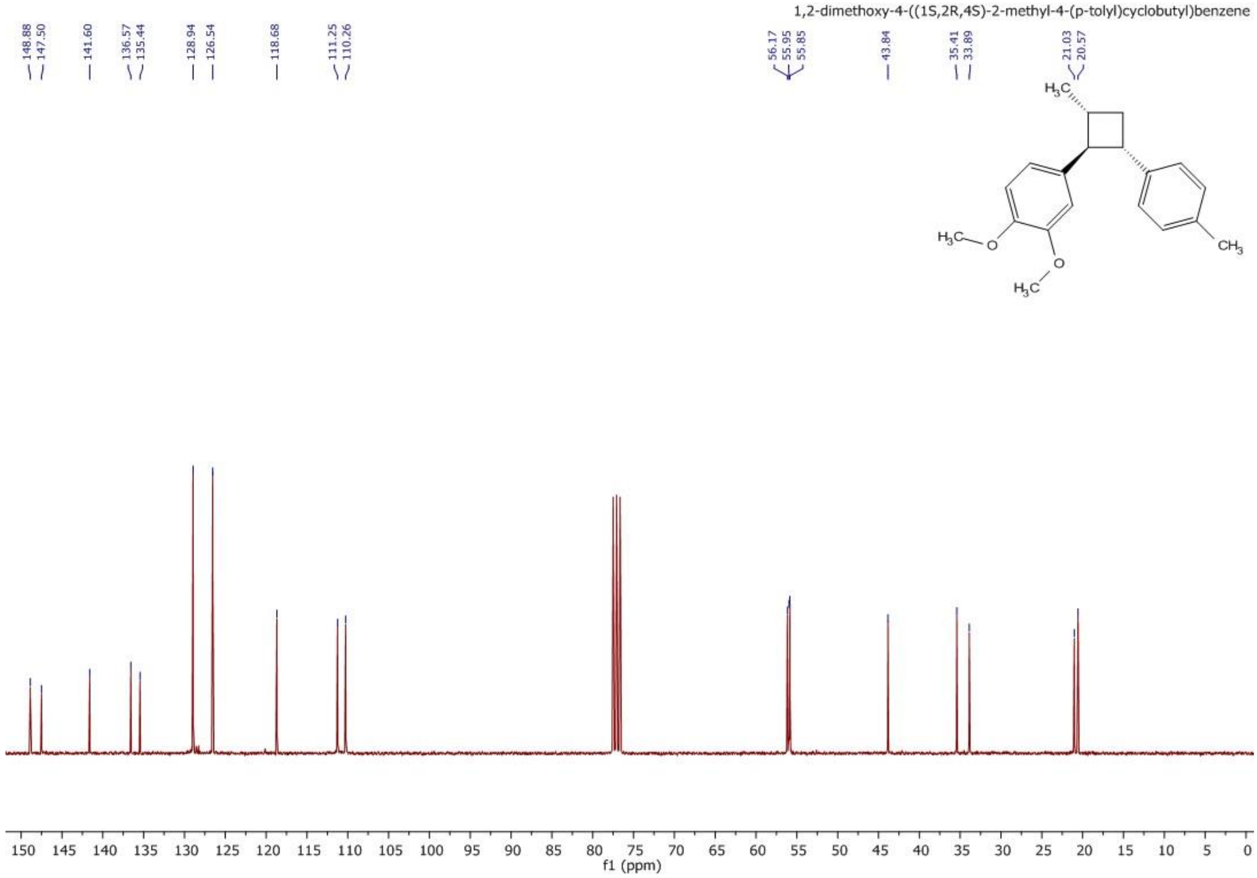

((1R,2R,3S)-2-(4-methoxyphenyl)-3-(p-tolyl)cyclobutyl)methanol

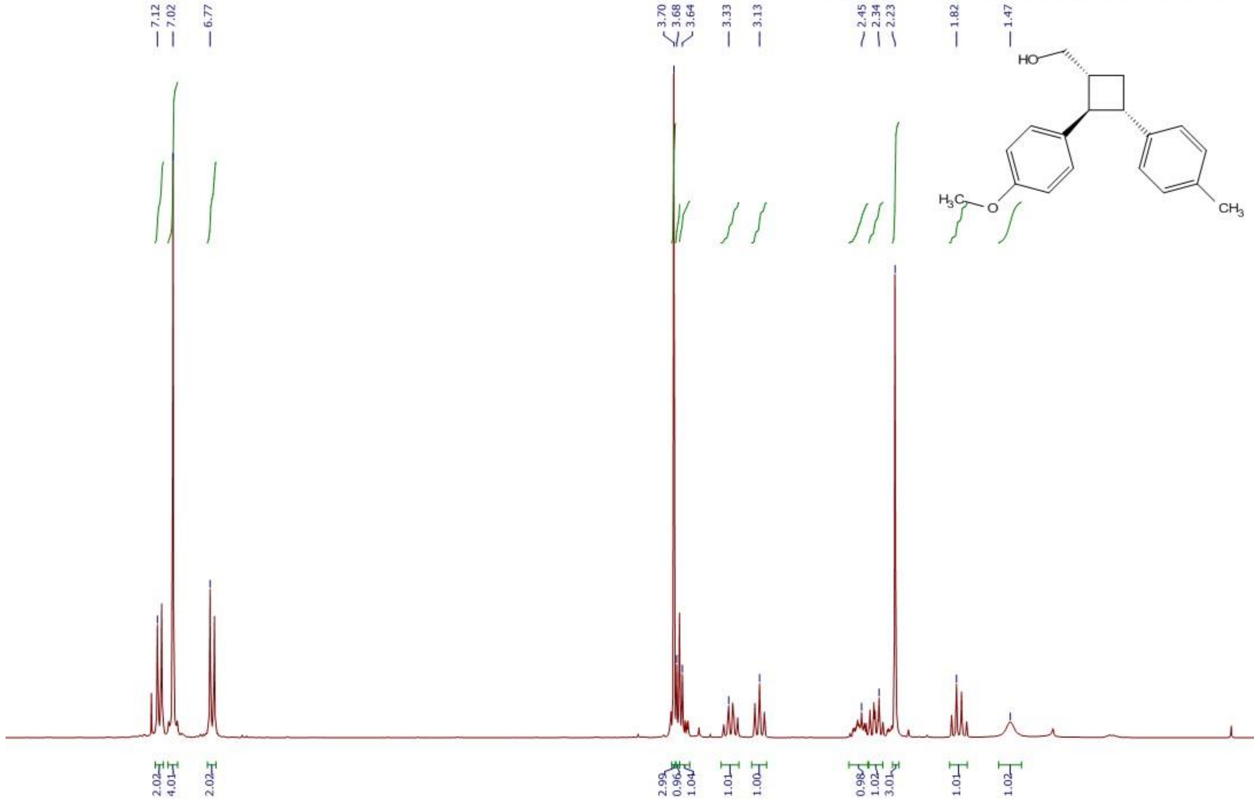

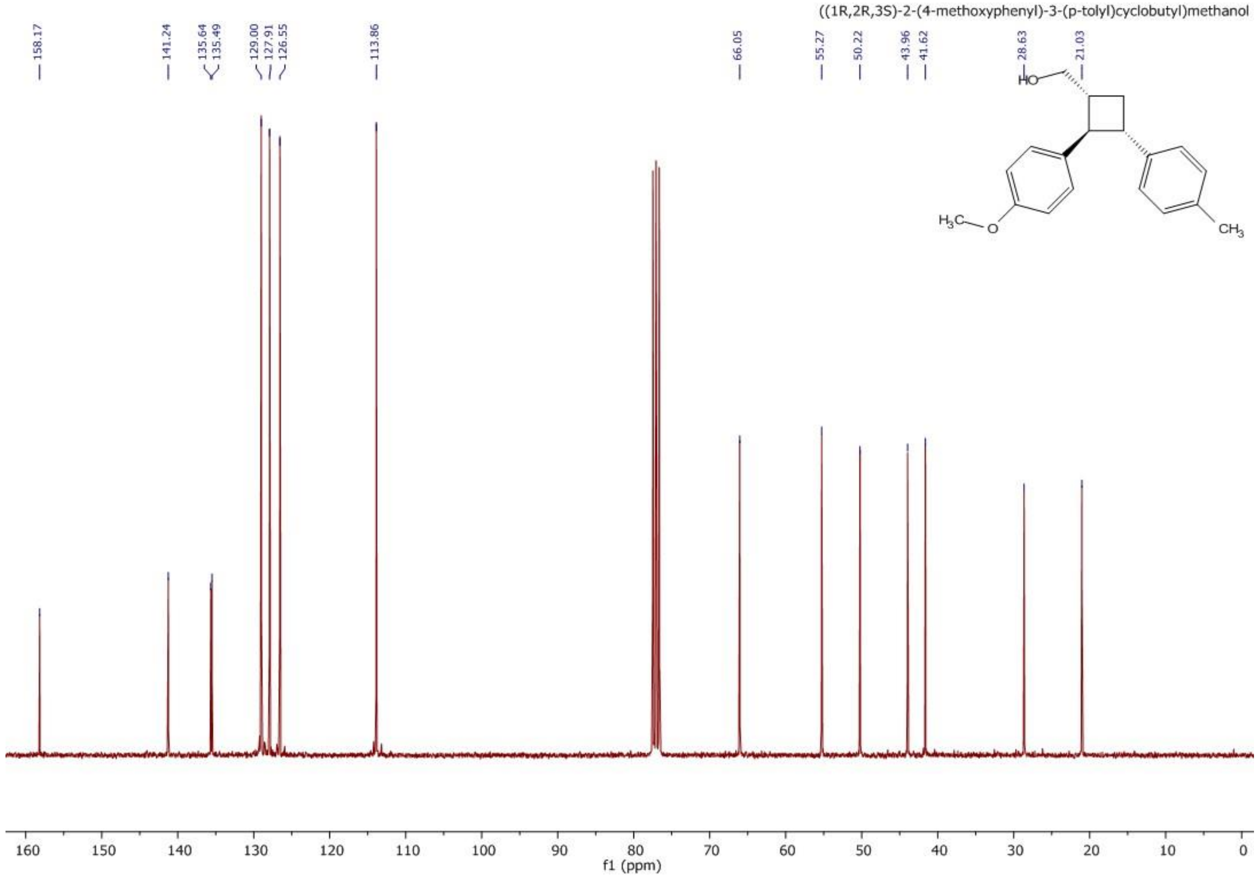

((1R,2R,3S)-2-(4-methoxyphenyl)-3-(p-tolyl)cyclobutyl)methanol
158.17
141.24
135.64
135.49
129.00
127.91
126.55
113.86
66.05
55.27
50.22
43.96
41.62
28.63
21.03
f1 (ppm)

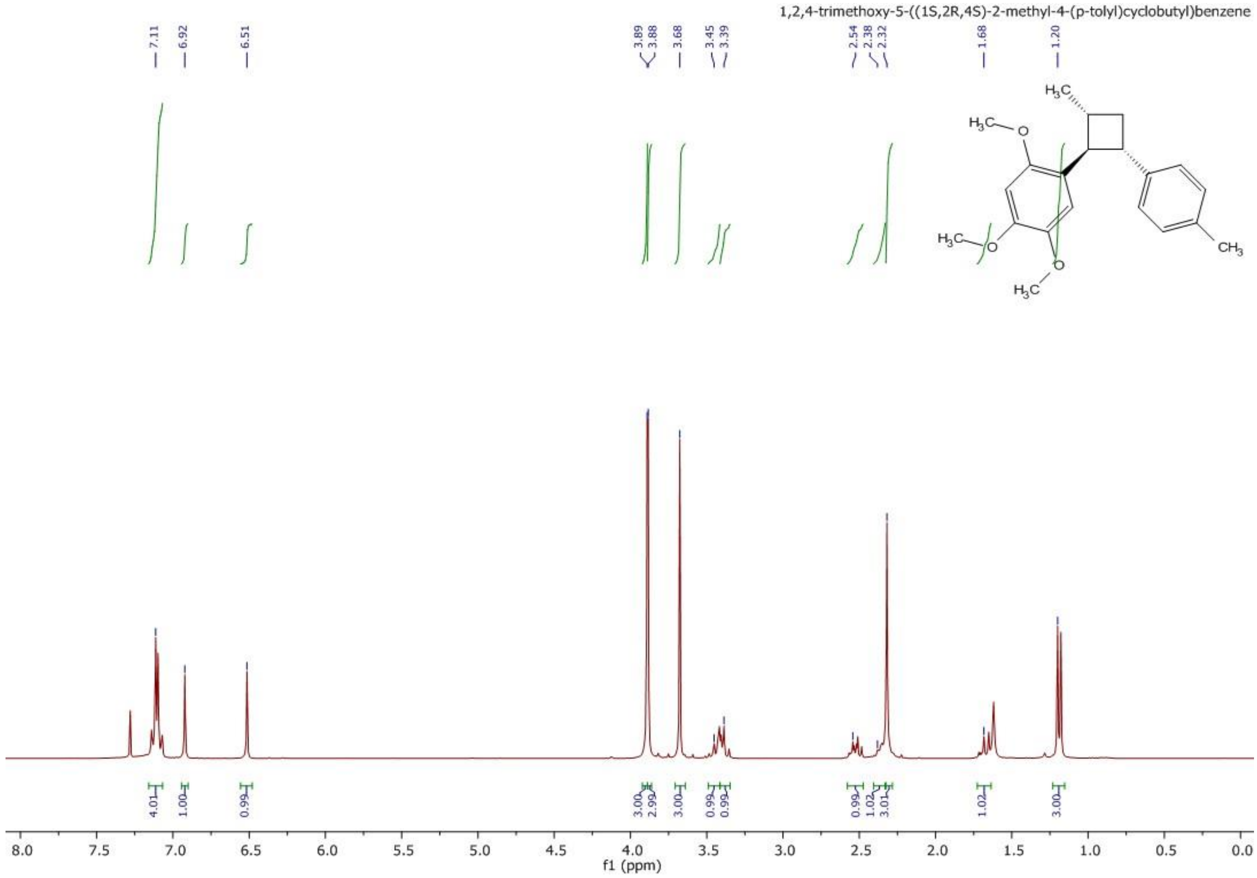

1,2,4-trimethoxy-5-((1S,2R,4S)-2-methyl-4-(p-tolyl)cyclobutyl)benzene
7.11
6.92
6.51
3.89
3.88
3.68
3.45
3.39
2.54
2.38
2.32
1.68
1.20
f1 (ppm)

1,2,4-trimethoxy-5-((1S,2R,4S)-2-methyl-4-(p-tolyl)cyclobutyl)benzene

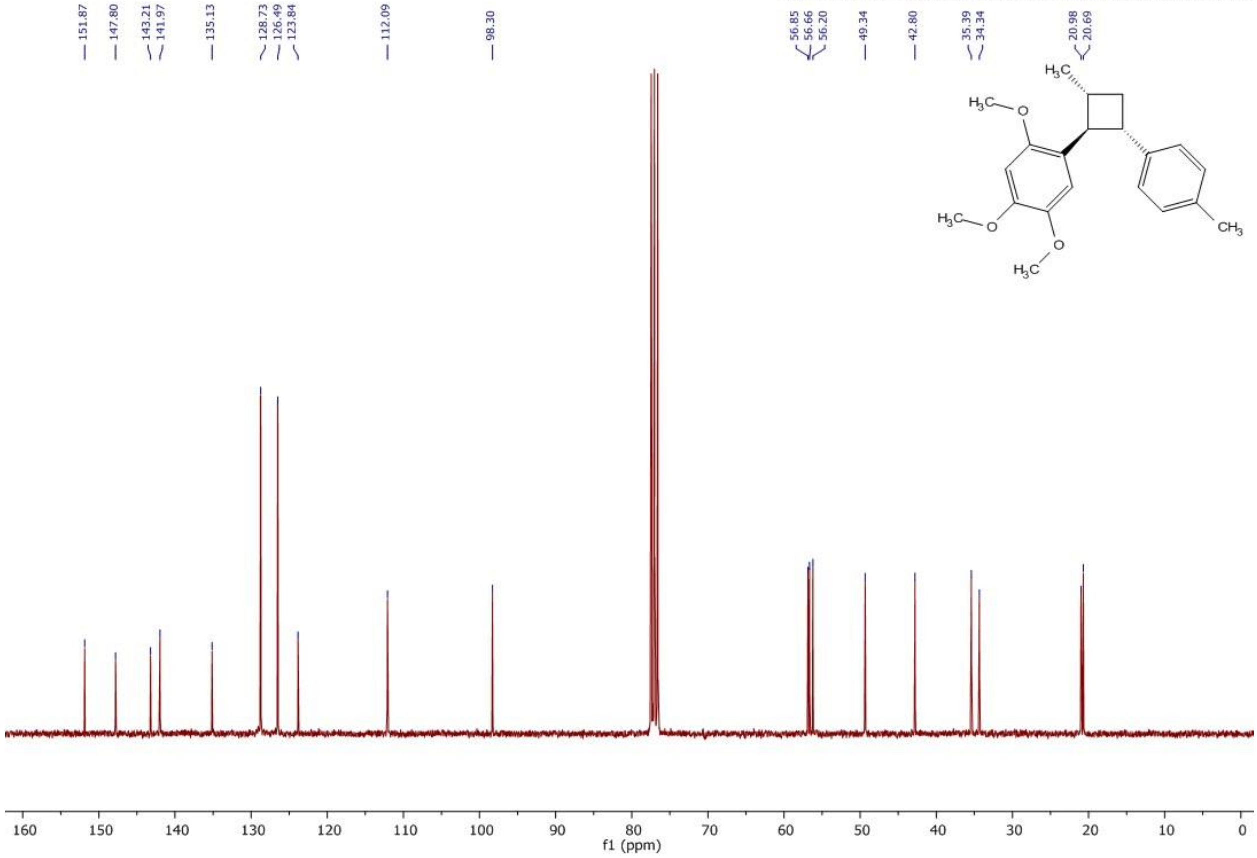

Supplement: Supplementary file 3 — Supplementary Data 1 [file 41467_2020_14983_MOESM3_ESM.pdf]
